# Differential gene expression in ADAM10 and mutant ADAM10 transgenic mice

**DOI:** 10.1186/1471-2164-10-66

**Published:** 2009-02-05

**Authors:** Claudia Prinzen, Dietrich Trümbach, Wolfgang Wurst, Kristina Endres, Rolf Postina, Falk Fahrenholz

**Affiliations:** 1 Johannes Gutenberg-University, Institute of Biochemistry, Mainz, Johann-Joachim-Becherweg 30, 55128 Mainz, Germany; 2 Helmholtz Zentrum München – German Research Center for Environmental Health, Institute for Developmental Genetics, Ingolstädter Landstraße 1, 85764 Neuherberg, Germany

## Abstract

**Background:**

In a transgenic mouse model of Alzheimer disease (AD), cleavage of the amyloid precursor protein (APP) by the α-secretase ADAM10 prevented amyloid plaque formation, and alleviated cognitive deficits. Furthermore, ADAM10 overexpression increased the cortical synaptogenesis. These results suggest that upregulation of ADAM10 in the brain has beneficial effects on AD pathology.

**Results:**

To assess the influence of ADAM10 on the gene expression profile in the brain, we performed a microarray analysis using RNA isolated from brains of five months old mice overexpressing either the α-secretase ADAM10, or a dominant-negative mutant (dn) of this enzyme. As compared to non-transgenic wild-type mice, in ADAM10 transgenic mice 355 genes, and in dnADAM10 mice 143 genes were found to be differentially expressed. A higher number of genes was differentially regulated in double-transgenic mouse strains additionally expressing the human APP_[V717I] _mutant.

Overexpression of proteolytically active ADAM10 affected several physiological pathways, such as cell communication, nervous system development, neuron projection as well as synaptic transmission. Although ADAM10 has been implicated in Notch and β-catenin signaling, no significant changes in the respective target genes were observed in adult ADAM10 transgenic mice.

Real-time RT-PCR confirmed a downregulation of genes coding for the inflammation-associated proteins S100a8 and S100a9 induced by moderate ADAM10 overexpression. Overexpression of the dominant-negative form dnADAM10 led to a significant increase in the expression of the fatty acid-binding protein Fabp7, which also has been found in higher amounts in brains of Down syndrome patients.

**Conclusion:**

In general, there was only a moderate alteration of gene expression in ADAM10 overexpressing mice. Genes coding for pro-inflammatory or pro-apoptotic proteins were not over-represented among differentially regulated genes. Even a decrease of inflammation markers was observed. These results are further supportive for the strategy to treat AD by increasing the α-secretase activity.

## Background

Accumulation of amyloid β-peptides (Aβ) in the brain is believed to contribute to the development of Alzheimer disease (AD). Soluble oligomeric forms of Aβ are neurotoxic [1-3]. Aβ, a 40–43 amino acid-comprising proteolytical fragment of the amyloid precursor protein (APP), is released from APP by sequential cleavages via β- and γ-secretases. However, the predominant route of APP processing consists of successive cleavages by α- and γ-secretases. Alpha-secretase attacks APP inside the Aβ sequence, and therefore prevents formation of neurotoxic Aβ. In addition, the soluble N-terminal domain of APP (APPsα) is released, which has neurotrophic and neuroprotective properties [4,5], and enhances LTP [6]. In behavioral paradigms, APPsα was demonstrated to improve memory in normal and amnesic mice [7]. Reduced amounts of APPsα were detected in the cerebrospinal fluid of AD patients [8,9].

Proteinases of the ADAM (adisintegrin and metalloproteinase) family are main candidates for physiologically relevant α-secretases, and we demonstrated that ADAM10 has α-secretase activity *in vitro *and in cultured cells [10]. ADAM10-deficient mice have been generated [11], but their early lethality at day E9.5 prevents a reliable analysis of ADAM10's α-secretase function *in vivo*, especially in neuronal cells.

To investigate whether an increase in activity of putative α-secretases *in vivo *prevents plaque formation and cognitive deficits, we generated transgenic mice overexpressing either the α-secretase ADAM10 (ADAM10 mice) or the catalytically inactive ADAM10_[E384A] _mutant (dnADAM10 mice) [12]. Neuronal overexpression of ADAM10 had no detrimental effects on ADAM10 single-transgenic mice: these animals exhibited normal behavioral abilities [13]. We found that a moderate neuronal overexpression of ADAM10 in mice carrying the human APP_[V717I] _mutation (ADAM10/APP_[V717I] _mice) increased the secretion of APPsα, reduced the formation of Aβ peptides, and prevented their deposition in plaques. Functionally, impaired long-term potentiation and cognitive deficits were alleviated. Expression of dominant-negative ADAM10_ [E384A] _in APP_[V717I] _mice (dnADAM10/APP_[V717I] _mice) led to reduction of APPsα and to enhancement of the number and size of amyloid plaques in the brain [12].

Histological analyses of mono-transgenic ADAM10 mice revealed an increase in cortical cholinergic, glutamatergic and GABAergic presynaptic bouton densities in 8 months old mice; the cholinergic presynaptic bouton density remained elevated even during aging in ADAM10 mice [14].

In addition to their metalloproteinase domain, ADAMs contain a disintegrin domain as a modulator of cell-cell and cell-matrix interactions [15]. As ADAM10 itself has been reported to be a substrate for ectodomain shedding by ADAM9 and subsequent cleavage by γ-secretase, the C-terminus of ADAM10 may represent a Notch-like signaling molecule [16]. Thus, independent of the catalytic activity of ADAM10, which has been implicated in Notch and β-catenin signaling, ADAM10 may also modulate gene expression via other domains.

To analyze the influence of ADAM10 and its dominant-negative form (dnADAM10) on the gene expression profile of the central nervous system (CNS), we investigated ADAM10 and dnADAM10 mice. We included in our study the double-transgenics ADAM10/APP_[V717I] _and dnADAM10/APP_[V717I]_. Since APP_[V717I] _mice show early phenotype changes (between months 4 and 7), we investigated the gene expression in 5 months old mice.

## Methods

### Animals

Animal husbandry was performed in accord with the guidelines of the German Council on Animal Care. All mouse strains (strain background FVB/N) analyzed in this study have been described previously [12]. The expression level of transgenic mature ADAM10 is 30% above endogenous levels and in dnADAM10 mice the expression of the catalytically inactive ADAM10 mutant is sevenfold above endogenous ADAM10 [12]. ADAM10 activity was determined in previous studies [12,17] by quantitation of the APP cleavage product APPsα. In ADAM10 overexpressing mice the catalytic activity of ADAM10 against its substrate APP_[V717I] _was increased to about 250%. In mice overexpressing dnADAM10, the endogenous APP_[V717I] _cleavage activity was reduced to about 25% as compared to APP_[V717I] _mice [12].

For the first experimental series of the present study, female ADAM10, dnADAM10 and FVB/N wild-type mice were investigated; for the second series, female and male ADAM10/APP_[V717I]_, dnADAM10/APP_[V717I] _and APP_[V717I] _mice were compared. In each case, brains of three 5 months old animals of each group were dissected and stored in RNA-later (Qiagen, Hilden, Germany) at -80°C to prevent RNA degradation.

### RNA preparation and microarray analyses

Total RNA from whole mouse brains was isolated by using the RNeasy Kit (Qiagen, Hilden, Germany), including on-column DNase I digestion according to the manufacturer's recommendations. The quality of isolated RNA was controlled by the Lab-on-Chip-System Bioanalyser 2100 (Agilent Technologies Inc., Palo Alto, CA, USA).

The expression-profiling analysis for mono-transgenic mice (ADAM10, dnADAM10 mice and non-transgenic FVB/N control animals) was carried out at RZPD (Berlin, Germany). Samples from double-transgenic mice (ADAM10/APP_[V717I_, dnADAM10/APP_[V717I] _and mono-transgenic APP_[V717I] _control mice) were analyzed at the Microarray Facility (Tübingen, Germany). In all cases, the Mouse Genome 430 2.0 Array (Affymetrix, Santa Clara, CA, USA) containing 45000 probe sets of 34000 genes was used for mRNA expression profiling.

### Statistical analysis and gene annotations

For the first series (mono-transgenic mice) 9 gene chip arrays and for the second series (double-transgenic mice) 18 gene chip arrays were analyzed. Data mining was performed by using the ChipInspector analysis software (Genomatix, Munich, Germany), which identifies significant changes based on single probes. The corresponding transcripts were then assigned after a user-defined number of significant probes. For all analyses, a transcript coverage greater than three probes was chosen. By this strategy, annotation errors and errors caused by the existence of alternative transcripts are reduced.

After total intensity normalization of each array, significantly changed genes were determined by significance analysis of microarrays (SAM) [18], using the exhaustive comparison mode at a false discovery rate (FDR) of 0.0% for double-transgenic, and 0.5% for mono-transgenic mice. For separate analysis of samples from double-transgenic female and male mice, a FDR of 1.3% was chosen. The resulting gene lists were restricted to the 600 most strongly regulated genes (up- as well as downregulated genes).

Regulated genes were then analyzed with the Bibliosphere software (Version 5.02; Genomatix, Munich, Germany) and mapped to Gene Ontology (GO) trees in order to identify their biological function. For identification of over-represented GO terms, the Bibliosphere software calculates a z-score for each term. The z-score represents the difference between observed and expected annotations, and is normalized to the standard deviation of a hypergeometric distribution. Only GO terms with a z-score > 1.96, which corresponds to a p-value of 0.05, have been considered.

To identify transcripts which are affected by ADAM10 and dnADAM10 overexpression in mono- and double-transgenic mice, we generated Venn diagrams with SAM-based gene lists. The expression profile of selected significantly regulated genes from microarrays was represented by heat maps using the R statistical software . Hierarchical clustering was applied to investigate whether expression values can be separated according to experimental groups. In this study, two heat maps were generated: one compared the expression profiles of genes in ADAM10 and dnADAM10 mono-transgenic mice, as well as in FVB/N non-transgenic control mice; a second one compared the expression profiles of double-transgenics and APP_[V717I] _mice.

Because the two series of expression arrays were measured in different laboratories, a global normalization procedure was needed to make them comparable. The default background noise adjustment, provided by the Affymetrix system, is based on the difference of perfect matching probes (PM) minus mismatching probes (MM). Due to unspecific binding, the global background adjustment method robust multi-array average (RMA) expression measure, which ignores the MM intensities, has been developed [19]. Because RMA adjustment does not completely remove unspecific intensities [20], an enhanced method denoted GeneChip RMA (GCRMA) has been designed [21] which considers the sequence of probes.

We performed background adjustment as well as quantile normalization for all data sets (raw format, cell files) with the GCRMA method (standard settings) by using the CARMAweb interface [22]. Subsequently, an unpaired two-tailed Student's t-test was applied for each respective gene to determine whether it is differentially expressed in the two sample groups. Since microarray analysis operates with large numbers of multiple comparisons, a false discovery rate-controlling method has to be applied. Therefore, by using the Benjamini-Hochberg (BH) method, adjusted p-values were calculated [23].

The GCRMA method is also appropriate for detection of minor changes in gene expression, and was required for comparative analysis of mono- and double-transgenic mice, due to the low intensities of the microarrays from the first series (mono-transgenic mice) as compared to those of the second series (double-transgenic mice). By comparing data derived from mono- and double-transgenic mice, we analyzed global biological trends of ADAM10 and dnADAM10 overexpression in FVB/N and FVB/N APP_[V717I] _strain backgrounds.

To identify transcripts which were commonly affected by APP_[V717I]_overexpression in all double-transgenic mice, we generated a Venn diagram with GCRMA-based gene lists (BH<0.005).

### Quantitative real-time RT-PCR

A two-step real-time reverse transcription (RT)-PCR was used to measure the expression of candidate genes. Isolated total RNA (1 μg) was used to synthesize cDNA in a 20 μl reaction with the QuantiTect Reverse Transcription Kit (Qiagen, Hilden, Germany) according to the manufacturer's manual. By adding water, the reaction volume was subsequently increased to 500 μl. Real-time RT-PCR was carried out in 96-well plates, using the 7000 ABI prism sequence detection system (Applied Biosystems, Darmstadt, Germany) and QuantiTect Primer Assays (Qiagen, Hilden, Germany). The primers for selected candidate genes are listed in table 1.

**Table 1:** QuantiTect Primer Assays (Qiagen, Hilden, Germany)

**Gene name**	**Gene ID**	***Assay***
ADAM10	11487	*Mm_Adam10_1_SG*
Fatty acid binding protein 7	12140	*Mm_Fabp7_1_SG*
Calcium binding protein S100a9	20202	*Mm_S100a9_1_SG*
Calcium binding protein S100a8	20201	*Mm_S100a8_1_SG*
Glutamate receptor, ionotropic, AMPA1	14799	*Mm_Gria_1_SG*
Glutamate receptor, ionotropic, AMPA2	14800	*Mm_Gria2_1_SG*
Low density lipoprotein receptor-related protein 1	16971	*Mm_Lrp1_1_SG*
Very low density lipoprotein receptor	22359	*Mm_Vldlr_1_SG*
Microtubule-associated protein tau	17762	*Mm_Mapt_1_SG*
Neuroligin 1	192167	*Mm_Nlgn1_1_SG*
GAPDH	14433	*Mm_Gapdh_2_SG*

Real-time RT-PCR reactions in a volume of 30 μl were performed in duplicate or triplicate under the following conditions: 5 μl of diluted cDNA (see above), 15 μl 2× QuantiTect PCR master mix (Qiagen, Hilden, Germany) and 300 nM of respective primer pair. After the initial denaturing and enzyme activation step (95°C for 15 min), 40 cycles (94°C for 15s, 55°C for 30s, and 72°C for 30s) were performed. A single DNA melting profile was observed in dissociation assay conditions demonstrating amplification of a unique product free of primer dimers.

For detection of Hes5 in 15 day old mice a one step Real-time RT-PCR was performed using the QuantiTect-SYBR-Green One-Step-RT PCR-Kit (Qiagen, Hilden, Germany) and 250 ng RNA in a reaction volume of 30 μl. The specific primer pair was as follows: Hes5RT_for 5'GAAAAACCGACTGCGGAAGCC3' and Hes5RT_rev 5'ACGGCCATCTCCAGGATGTC3'.

For data analysis, the threshold cycle (Ct) which indicates the relative abundance of a particular transcript, was calculated. Standard curves were generated by amplification of serially diluted cDNA. According to this method, the amount of all relevant genes was normalized to the amount of endogenous GAPDH present in the same sample. Measured values from control samples (non-transgenic FVB/N mice or mono-transgenic APP_[V717I] _mice) were set to 100%. Changes in gene expression are presented as the mean of alteration ± SD. The data were analyzed for statistical significance using one-way ANOVA (*, p < 0.05; **, p < 0.01; ***, p < 0.001).

### Western blotting

Mouse brain tissue was stored on dry ice immediately after dissection. Ice-cold TRIS buffer (20 mM Tris/HCl, pH 8.5) containing proteinase inhibitors (Inhibitor complete mini, Roche Diagnostics Corp., Mannheim, Germany) was added, and tissue was homogenized in a tissue lyser (Qiagen, Hilden, Germany). The supernatants resulting from centrifugation at 34000 rpm for 1.75 hours were separated on 14% SDS-gels and transferred to nitrocellulose membrane by tank blot system (40 μg protein per sample). For the detection and quantification of soluble FABP7 antibody AB9558 (Chemicon, Temecula, USA), and the appropriate horseradish peroxidase-coupled secondary antibody (Pierce, Rockford, USA) were used.

### ELISA

Hemispheres of mouse brain were weighed, proteins extracted and calprotectin (S100a8/a9) was quantified as recommended by the ELISA manufacturer (Immundiagnostik, Bensheim, Germany). In brief, tissue was homogenized in extraction buffer for 2 min at 20 Hz in a tissue lyzer and extraction was performed for 20 min at 4°C under agitation. After centrifugation (14000 rpm, 15 min) the supernatant and protein standards were added to microtiter plates in a total volume of 100 μl in duplicates. Incubation of the plate and measurement of optical densities at 405 nm were performed following the manufacturer's instructions. The relative amount of calprotectin was calculated by division of background-corrected values by wet tissue weight.

## Results

### Microarray analysis of gene regulation in ADAM10-transgenic mice

We performed microarray analysis with cDNA transcribed from total RNA of the brains of mice aged five months. Mono-transgenic ADAM10 as well as dnADAM10 mice were investigated in comparison to non-transgenic FVB/N wild-type mice (n = 3 females), to analyze the influence of the α-secretase ADAM10 or its catalytically inactive form (dnADAM10) on the gene expression profile of the CNS.

To elucidate the effect of ADAM10 and dnADAM10 on gene expression in an APP background, we compared samples derived from double-transgenic ADAM10/APP_[V717I] _and dnADAM10/APP_[V717I] _mice with those from mono-transgenic APP_[V717I] _mice. Because we wanted to test whether the modulation of ADAM10 activity might be a risk to the adult organism in respect to future therapeutic approaches, we chose 5 months old mice for our investigations. At this age, APP_[V717I] _animals show cognitive deficits, whereas amyloid plaque formation occurs several months later [24].

**Figure 1:**
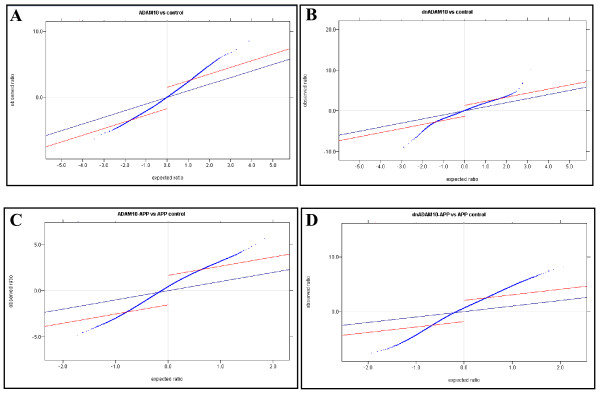
**Significance analysis of microarrays**. The SAM plots represent the differentially expressed genes of mono-transgenic (A and B with FDR 0.5), and double-transgenic mice (C and D with FDR 0.0). The Delta parameter, represented by red lines, defines the significance field (-1.72/+2.23 (A: ADAM10 versus FVB/N), -1.36/+1.43 (B: dnADAM10 versus FVB/N), -1.56/+2.06 (C: ADAM10/APP_[V717I] _versus APP_[V717I]_), -2.85/+3.29 (D: dnADAM10/APP_[V717I] _versus APP_[V717I]_)). Shown above the upper line are the genes upregulated significantly, and below the lower line the genes downregulated significantly.

The SAM plots in Fig. 1 represent the distribution of all probe signals on the microarray chip. Depending on the statistical stringency (FDR, delta) as represented by the red lines, significant probes are selected. Probe signals between the red lines are not significant, signals above the upper line correspond to significantly upregulated genes; signals below the lower line correspond to significantly downregulated genes. Tables 2 and 3 show the numbers of these differentially expressed genes.

**Table 2:** Numbers of significantly regulated genes in mono-transgenic mice (5 months, 3 females per group) restricted by the given d-values.

**Mouse Genome 430 2.0 Array (Affymetrix)**			
45 000 probe sets, 39 000 transcripts, 34 000 characterized			
ADAM10 versus FVB/N (wild-type) 355 genes, FDR = 0.5%		dnADAM10 versus FVB/N (wild-type) 143 genes, FDR = 0.5%	

300 upregulated (d-value > 2.23)	55 downregulated (d-value < -1.72)	50 upregulated (d-value > 1.43)	93 downregulated (d-value < -1.36)

**Table 3:** Numbers of significantly regulated genes in double-transgenic mice (5 months, 3 females and 3 males per group) restricted by the given d-values.

**Mouse Genome 430 2.0 Array (Affymetrix)**			
45 000 probe sets, 39 000 transcripts, 34 000 characterized			
ADAM10/APP_[V717I] _versus APP_[V717I]_ 592 genes, FDR = 0.0%		dnADAM10/APP_[V717I] _versus APP_[V717I]_ 600 genes, FDR = 0.0%	

295 upregulated (d-value > 2.06)	297 downregulated (d-value < -1.56)	300 upregulated (d-value > 3.29)	300 downregulated (d-value < -2.85)

The comparison of samples from ADAM10 and FVB/N mice revealed 355 differentially expressed genes: 300 genes were up- and 55 genes were downregulated. In dnADAM10 mice, the number of regulated genes was lower; as compared to FVB/N mice, 143 genes were differentially expressed. Among these, 50 genes were up- and 93 genes downregulated (Tab. 2).

Against the background of APP_[V717I] _overexpression, generally more genes were found to be differentially expressed. As compared to APP_[V717I] _mice, 592 genes (295 up- and 297 downregulated) were differentially expressed in ADAM10/APP_[V717I] _mice, and more than 600 genes in dnADAM10/APP_[V717I] _animals (Tab. 3). In the latter, the number of significantly regulated genes was restricted to 600, including the highest up- and downregulated genes. For the complete list of significantly regulated genes, see Additional file 1, Tables S1-S4. The data presented in this publication have been deposited in NCBI's Gene Expression Omnibus (GEO), and are accessible by the GEO Series accession numbers GSE10908 and GPL1261 .

**Figure 2:**
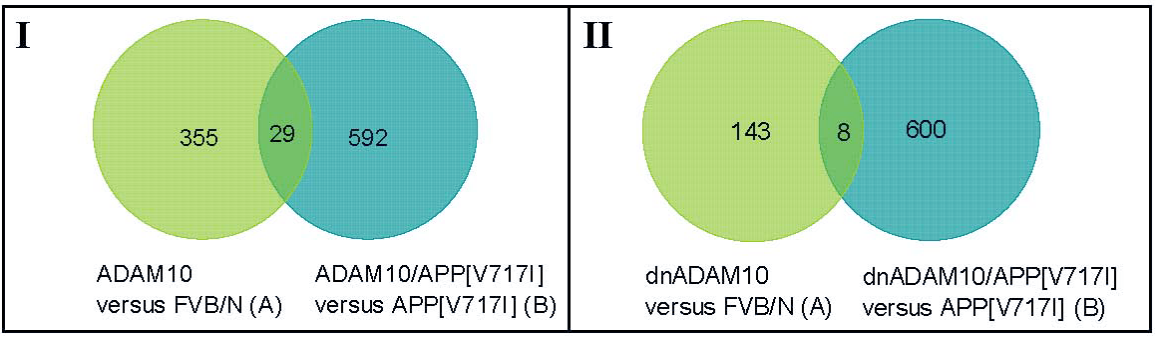
**Venn diagrams with SAM-based gene lists of mono- and double-transgenic mice**. Venn diagram I (ADAM10 versus FVB/N (A) is compared with ADAM10/APP_[V717I] _versus APP_[V717I] _(B)), and Venn diagram II (dnADAM10 versus FVB/N (A) is compared with dnADAM10/APP_[V717I] _versus APP_[V717I] _(B)), generated by a custom-written Perl-script, show the effects of the overexpression of ADAM10 and dnADAM10 in mono- and double-transgenic mice. The numbers in the space of overlapping circles represent the number of transcripts that were affected in both mouse lines.

For detection of transcripts that were commonly regulated by either ADAM10 or dnADAM10 overexpression in mono- and double-transgenic mice, Venn diagrams were generated with SAM-based gene lists (Fig. 2). The comparison of ADAM10 versus FVB/N (355 genes), and ADAM10/APP_[V717I] _versus APP_[V717I] _(592 genes) revealed 29 genes which were regulated by ADAM10 overexpression in either mono- or double-transgenic mice (Additional file 1, Tab. S5). When dnADAM10 versus FVB/N (143 genes) and dnADAM10/APP_[V717I] _versus APP_[V717I]_, were compared, only eight genes were identified to be commonly regulated by dnADAM10 overexpression (Additional file 1, Tab. S6). This result indicates that the genetic background strongly influences the effect of ADAM10 on gene expression.

### Common genetic profile in mono- and double-transgenic animals

**Figure 3:**
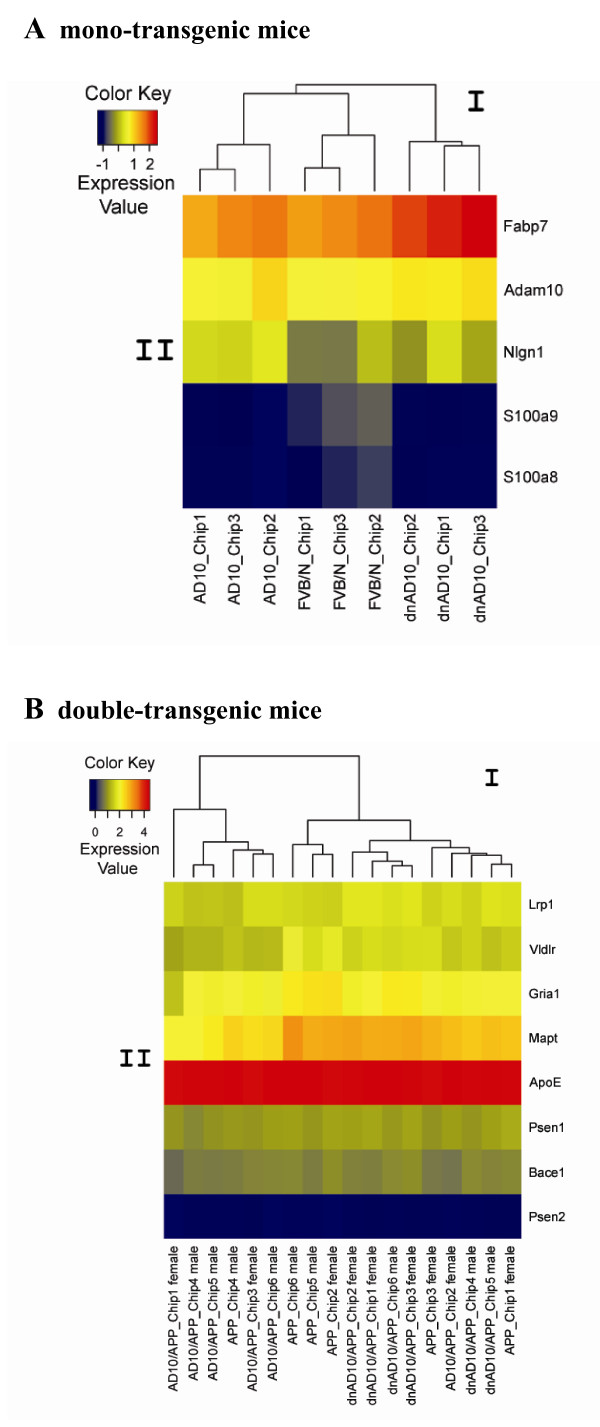
**Heat map representing the clustering of genes in mono- and double transgenic mice**. A) ADAM10, dnADAM10 and FVB/N mice; B) AD10/APP_[V717I]_, dnAD10/APP_[V717I]_, and APP_[V717I] _mice. Selected significantly regulated genes on individual chips are shown. The upper graph (I) represents the hierarchical clustering, the colored scales (II) the difference in gene expression. Unsupervised cluster analysis showed that the expression profiles of mono-transgenic mouse genes (A) cluster according to the experimental conditions. In case of double-transgenic mice (B), cluster analysis revealed a more rough agreement in the discrimination of gene expression with experimental groups. The blue (lower expression) to red (higher expression) color scale indicates 2-based logarithms of the mean expression values of the single probes after ChipInspector normalization (Genomatix, Munich, Germany).

Heat maps (Fig. 3) indicate that the chips of each series had their own characteristic genetic profile. For heat maps, genes of special interest were chosen (mono-transgenic mice: Adam10, Fabp7, S100a8, S100a9, Nlgn1; double-transgenic mice: Mapt, Gria1, Vldlr, Lrp1, Bace1, Psen1, Psen2, ApoE). The heat map in Fig. 3A reveals that in mice overexpressing bovine ADAM10, approximately the same amount of murine Adam10 is expressed as compared to wild-type mice (nearly all over yellow coloring). Fabp7 is distinctly higher expressed in all dnADAM10 mice (red color) in contrast to wild-type mice (orange color). The expression of Nlgn1 in ADAM10 and dnADAM10 mice is higher (yellow to green) than in FVB/N mice (green color)). Finally, S100a8 and S100a9 show lower expression in ADAM10 and dnADAM10 mice (blue color) in relation to FVB/N wild-type mice (yellow to blue). These results are in accordance with the observations made by the real-time RT-PCR as described below. Furthermore, hierarchical clustering showed that the expression profiles of the mono-transgenic mouse genes are separated to the original conditions. In the case of heat map in Fig. 3B, the small differences in the expression of Mapt, Gria1, Vldlr and Lrp1 are fitting to the results of real-time RT-PCR analyses as described below. Hierarchical clustering revealed that the expression profiles of double-transgenic mice genes are not clearly clustered according to the experimental settings, presumably due to the more complex conditions caused by APP overexpression. Also, a clear distinction between male and female mice could not be observed.

### ADAM10-regulated biological pathways

For pathway analysis, the complete gene lists were analyzed with the Bibliosphere software (Genomatix) and mapped to Gene Ontology (GO) trees.

Functional groups are only listed when their z-score of individual GO-categories is higher than 1.96. With respect to the known cellular function of disintegrin metalloproteases in general, and the α-secretase activity of ADAM10 in particular, we investigated biological processes including cell communication (GO:0007154), nervous system development (GO:0007399), cell adhesion (GO:0007155) and cell death (GO:0008219). Furthermore, we examined neuron projection (GO:0043005), synaptic junction (GO:0045202) and transmission (GO:0007268). At the molecular level, we focused on receptor binding (GO:0005102) and receptor activity (GO:0004872) (Tab. 4, 5, 6, 7).

**Table 4:** Significantly regulated genes in mono-transgenic ADAM10 mice in relation to FVB/N wild-type mice

**Gene ID**	**Gene symbol**	**Description**	**Fold change**	**log ratio**	**d-value**	**Functional groups**
27360	Add3	adducin 3 (gamma)	1.519	0.603	2.841	receptor binding and activity
68465	Adipor2	adiponectin receptor 2	1.365	0.449	2.294	cell communication
11658	Alcam	activated leukocyte cell adhesion molecule	1.471	0.557	2.298	nervous system development; neuron projection
211673	Arfgef1	ADP-ribosylation factor guanine nucleotide-exchange factor 1(brefeldin A-inhibited)	1.503	0.588	2.644	cell communication
11855	Arhgap5	Rho GTPase activating protein 5	1.621	0.697	2.526	cell communication
98660	Atp1a2	ATPase, Na+/K+ transporting, alpha 2 polypeptide	0.792	-0.337	-1.972	cell communication; synapse
11941	Atp2b2	ATPase, Ca++ transporting, plasma membrane 2	1.734	0.794	2.717	cell communication; nervous system development; receptor binding and activity; synapse
22589	Atrx	alpha thalassemia/mental retardation syndrome X-linked homolog (human)	1.507	0.592	2.876	nervous system development
30948	Bin1	bridging integrator 1	1.451	0.537	2.318	cell communication; synapse
12298	Cacnb4	calcium channel, voltage-dependent, beta 4 subunit	1.800	0.848	2.913	cell communication; synapse
12322	Camk2a	calcium/calmodulin-dependent protein kinase II alpha	1.779	0.831	2.547	cell communication; receptor binding and activity; synapse
16149	Cd74	CD74 antigen (invariant polypeptide of major histocompatibility complex, class II antigen-associated)	0.724	-0.466	-2.270	cell communication; cell death
212285	Centd1	centaurin, delta 1	1.569	0.650	2.530	cell communication
12633	Cflar	CASP8 and FADD-like apoptosis regulator	1.393	0.478	2.497	cell death
12704	Cit	citron	1.476	0.562	2.405	cell communication; nervous system development
12803	Cntf	ciliary neurotrophic factor	1.496	0.581	2.355	cell communication; nervous system development
70086	Cysltr2	cysteinyl leukotriene receptor 2	1.307	0.386	2.251	cell communication
13618	Ednrb	endothelin receptor type B	1.364	0.448	2.506	cell communication; nervous system development
13838	Epha4	Eph receptor A4	1.622	0.698	2.439	cell communication; nervous system development
67456	Ergic2	ERGIC and golgi 2	1.367	0.451	2.316	cell communication; synapse
14397	Gabra4	gamma-aminobutyric acid (GABA-A) receptor, subunit alpha 4	1.542	0.625	2.316	cell communication; synapse
14417	Gad2	glutamic acid decarboxylase 2	1.636	0.710	3.290	cell communication; neuron projection; synapse
14674	Gna13	guanine nucleotide binding protein, alpha 13	1.502	0.587	2.257	cell communication
14677	Gnai1	guanine nucleotide binding protein, alpha inhibiting 1	1.576	0.656	2.911	cell communication
14680	Gnal	guanine nucleotide binding protein, alpha stimulating, olfactory type	1.598	0.676	3.061	cell communication
53623	Gria3	glutamate receptor, ionotropic, AMPA3 (alpha 3)	1.480	0.566	2.388	synapse
56637	Gsk3b	glycogen synthase kinase 3 beta	1.540	0.623	2.382	cell communication; cell death
15208	Hes5	hairy and enhancer of split 5 (Drosophila)	0.749	-0.416	-1.800	nervous system development
16419	Itgb5	integrin beta 5	0.786	-0.347	-2.185	cell adhesion; cell communication
16510	Kcnh1	potassium voltage-gated channel, subfamily H (eag-related), member 1	1.398	0.483	2.291	cell communication; receptor binding and activity
16561	Kif1b	kinesin family member 1B	1.555	0.637	2.573	cell communication; synapse
16573	Kif5b	kinesin family member 5B	1.904	0.929	3.186	neuron projection; synapse
16574	Kif5c	kinesin family member 5C	1.656	0.728	2.463	nervous system development; neuron projection; synapse
110829	Lims1	LIM and senescent cell antigen-like domains 1	1.533	0.616	2.309	cell adhesion
108030	Lin7a	lin-7 homolog A (C. elegans)	1.346	0.429	2.260	cell communication; synapse
319387	Lphn3	latrophilin 3	1.295	0.373	2.344	cell communication
16971	Lrp1	low density lipoprotein receptor-related protein 1	0.707	-0.500	-2.139	cell communication
16998	Ltbp3	latent transforming growth factor beta binding protein 3	0.791	-0.338	-2.180	cell communication; receptor binding and activity
50791	Magi2	membrane associated guanylate kinase, WW and PDZ domain containing 2	1.426	0.512	2.399	cell communication
192167	Nlgn1	neuroligin 1	1.518	0.602	2.386	cell communication; nervous system development; synapse
18549	Pcsk2	proprotein convertase subtilisin/kexin type 2	1.492	0.577	2.393	nervous system development
18573	Pde1a	phosphodiesterase 1A, calmodulin-dependent	1.796	0.845	3.130	cell communication; receptor binding and activity
18596	Pdgfrb	platelet derived growth factor receptor, beta polypeptide	0.800	-0.322	-1.867	cell communication
18613	Pecam1	platelet/endothelial cell adhesion molecule 1	0.778	-0.363	-2.093	cell communication
18795	Plcb1	phospholipase C, beta 1	1.652	0.724	3.345	cell communication
18798	Plcb4	phospholipase C, beta 4	1.480	0.566	2.451	cell communication
242083	Ppm1l	protein phosphatase 1 (formerly 2C)-like	1.604	0.682	2.277	cell communication
26932	Ppp2r5e	protein phosphatase 2, regulatory subunit B (B56), epsilon isoform	1.578	0.658	2.298	cell communication
19281	Ptprt	protein tyrosine phosphatase, receptor type, T	1.409	0.495	2.354	cell communication
19328	Rab12	RAB12, member RAS oncogene family	1.250	0.322	2.259	cell communication
270192	Rab6b	RAB6B, member RAS oncogene family	1.696	0.762	2.837	cell communication; synapse
56044	Rala	v-ral simian leukemia viral oncogene homolog A (ras related)	1.582	0.662	2.544	cell communication
54409	Ramp2	receptor (calcitonin) activity modifying protein 2	1.693	0.760	2.640	cell communication
218397	Rasa1	RAS p21 protein activator 1	1.428	0.514	2.266	cell adhesion; cell communication; cell death
19737	Rgs5	regulator of G-protein signaling 5	1.677	0.746	2.390	cell communication
19894	Rph3a	rabphilin 3A	1.470	0.556	2.352	synapse
68585	Rtn4	reticulon 4	1.542	0.625	2.344	cell death; nervous system development
20202	S100a9	S100 calcium binding protein A9	0.668	-0.582	-2.146	cell communication
20377	Sfrp1	secreted frizzled-related sequence protein 1	0.793	-0.335	-1.730	cell communication
239250	Slitrk6	SLIT and NTRK-like family, member 6	1.324	0.405	2.293	nervous system development
93761	Smarca1	SWI/SNF related, matrix associated, actin dependent regulator of chromatin, subfamily a, member 1	1.309	0.388	2.351	nervous system development
66042	Sostdc1	sclerostin domain containing 1	0.725	-0.464	-1.768	cell communication
20742	Spnb2	spectrin beta 2	1.729	0.790	2.855	cell communication; receptor binding and activity
114716	Spred2	sprouty-related, EVH1 domain containing 2	1.514	0.598	2.653	cell communication
21961	Tns1	tensin 1	1.505	0.590	2.245	cell communication
22370	Vtn	vitronectin	0.778	-0.362	-1.925	cell adhesion
22371	Vwf	Von Willebrand factor homolog	0.715	-0.484	-2.099	cell adhesion
57750	Wdr12	WD repeat domain 12	1.391	0.476	2.446	cell communication
232341	Wnk1	WNK lysine deficient protein kinase 1	1.844	0.883	2.947	cell communication
22772	Zic2	Zinc finger protein of the cerebellum 2	1.941	0.957	3.062	nervous system development

**Table 5:** Significantly regulated genes in mono-transgenic dnADAM10 mice in relation to FVB/N wild-type mice

**Gene ID**	**Gene symbol**	**Description**	**Fold change**	**log ratio**	**d-value**	**Functional groups**
22589	Atrx	alpha thalassemia/mental retardation syndrome X-linked homolog (human)	1.392	0.477	1.533	nervous system development
109880	Braf	Braf transforming gene	0.568	-0.815	-5.060	cell communication
54598	Calcrl	calcitonin receptor-like	0.626	-0.675	-2.137	cell communication
12322	Camk2a	calcium/calmodulin-dependent protein kinase II alpha	0.686	-0.544	-2.523	cell communication; receptor binding and activity; synapse
12772	Ccr2	chemokine (C-C motif) receptor 2	0.724	-0.465	-1.783	cell communication
16149	Cd74	CD74 antigen (invariant polypeptide of major histocompatibility complex, class II antigen-associated)	0.737	-0.441	-2.329	cell communication; cell death
13175	Dcamkl1	doublecortin and calcium/calmodulin-dependent protein kinase-like 1	1.501	0.586	1.610	nervous system development
12140	Fabp7	fatty acid-binding protein 7	1.691	0.758	2.107	nervous system development
14281	Fos	FBJ osteosarcoma oncogene	0.669	-0.58	-1.805	nervous system development
14417	Gad2	glutamic acid decarboxylase 2	1.422	0.508	1.683	cell communication; neuron projection; synapse
14674	Gna13	guanine nucleotide binding protein, alpha 13	1.353	0.436	1.639	cell communication
14682	Gnaq	guanine nucleotide binding protein, alpha q polypeptide	1.323	0.404	1.621	cell communication; nervous system development; synapse
15557	Htr1f	5-hydroxytryptamine (serotonin) receptor 1F	1.363	0.447	1.539	cell communication
16594	Klc2	kinesin light chain 2	0.789	-0.341	-1.485	neuron projection
207911	Mchr1	melanin-concentrating hormone receptor 1	0.718	-0.477	-3.057	cell communication
17260	Mef2c	myocyte enhancer factor 2C	1.433	0.519	1.507	nervous system development
18823	Plp1	proteolipid protein (myelin) 1	1.358	0.442	1.565	cell communication; nervous system development;
19293	Pvalb	parvalbumin	0.847	-0.24	-1.493	neuron projection
19317	Qk	quaking	1.312	0.392	1.522	cell communication; nervous system development
54409	Ramp2	receptor (calcitonin) activity modifying protein 2	1.604	0.682	1.623	cell communication
19736	Rgs4	regulator of G-protein signaling 4	1.339	0.421	1.843	cell communication
20202	S100a9	S100 calcium binding protein A9	0.696	-0.522	-2.181	cell communication

**Table 6:** Significantly regulated genes in double-transgenic ADAM10/APP_[V717I] _mice in relation to mono-transgenic APP_[V717I] _mice

**Gene ID**	**Gene symbol**	**Description**	**Fold change**	**log ratio**	**d-value**	**Functional groups**
56215	Acin1	apoptotic chromatin condensation inducer 1	0.795	-0.331	-1.955	cell communication; cell death
329910	Acot11	acyl-CoA thioesterase 11	0.812	-0.301	-1.620	cell communication
432530	Adcy1	adenylate cyclase 1	0.810	-0.304	-1.880	cell communication; receptor binding and activity
68465	Adipor2	adiponectin receptor 2	0.781	-0.357	-1.868	cell communication
11540	Adora2a	adenosine A2a receptor	1.294	0.372	2.210	cell communication; synapse
11735	Ank3	ankyrin 3, epithelial	1.344	0.427	2.159	cell communication; nervous system development; synapse
11787	Apbb2	amyloid beta (A4) precursor protein-binding, family B, member 2	0.788	-0.344	-2.018	cell communication; cell death; nervous system development
226548	Aph1a	anterior pharynx defective 1a homolog (C. elegans)	0.807	-0.309	-1.879	cell communication
76117	Arhgap15	Rho GTPase activating protein 15	1.275	0.351	2.139	cell communication
76294	Asb5	ankyrin repeat and SOCs box-containing protein 5	1.254	0.327	2.079	cell communication
98660	Atp1a2	ATPase, Na+/K+ transporting, alpha 2 polypeptide	0.820	-0.287	-1.746	cell communication; synapse
12043	Bcl2	B-cell leukemia/lymphoma 2	0.815	-0.295	-1.741	cell communication; cell death
72567	Bclaf1	BCL2-associated transcription factor 1	1.326	0.407	2.183	cell death
12122	Bid	BH3 interacting domain death agonist	1.291	0.369	2.278	cell death
109880	Braf	Braf transforming gene	0.812	-0.301	-1.837	cell communication
12227	Btg2	B-cell translocation gene 2, anti-proliferative	1.342	0.424	2.357	cell death
12300	Cacng2	calcium channel, voltage-dependent, gamma subunit 2	0.803	-0.316	-1.872	cell communication
12325	Camk2g	calcium/calmodulin-dependent protein kinase II gamma	0.810	-0.304	-1.711	receptor binding and activity
12319	Car8	carbonic anhydrase 8	0.785	-0.349	-1.679	cell communication
12361	Cask	calcium/calmodulin-dependent serine protein kinase (MAGUK family)	0.832	-0.265	-1.689	receptor binding and activity; synapse
226751	Cdc42bpa	Cdc42 binding protein kinase alpha	1.288	0.365	2.130	cell communication
12575	Cdkn1a	cyclin-dependent kinase inhibitor 1A (P21)	0.779	-0.36	-2.004	cell death
235415	Cplx3	complexin 3	1.276	0.352	2.294	cell communication; synapse
12955	Cryab	crystallin, alpha B	0.744	-0.427	-2.161	cell communication
12977	Csf1	colony stimulating factor 1 (macrophage)	0.822	-0.282	-1.717	cell adhesion; cell communication; receptor binding and activity
27373	Csnk1e	casein kinase 1, epsilon	0.791	-0.338	-2.160	cell communication
13000	Csnk2a2	casein kinase 2, alpha prime polypeptide	0.801	-0.321	-1.909	cell communication
16007	Cyr61	cysteine rich protein 61	1.298	0.376	2.213	receptor binding and activity
54722	Dfna5h	deafness, autosomal dominant 5 homolog (human)	0.803	-0.316	-1.714	cell communication
330938	Dixdc1	DIX domain containing 1	0.825	-0.278	-1.770	cell communication
50768	Dlc1	deleted in liver cancer 1	0.794	-0.332	-1.894	cell communication
13430	Dnm2	dynamin 2	0.815	-0.295	-2.004	synapse
13527	Dtna	dystrobrevin alpha	1.271	0.346	2.240	synapse
13841	Epha7	Eph receptor A7	1.300	0.379	2.146	cell communication; nervous system development
14254	Flt1	FMS-like tyrosine kinase 1	0.787	-0.345	-2.064	cell communication
118446	Gje1	gap junction membrane channel protein epsilon 1	1.349	0.432	2.453	cell communication;
69367	Glrx2	glutaredoxin 2 (thioltransferase)	1.252	0.324	2.066	cell communication; cell death
14682	Gnaq	guanine nucleotide binding protein, alpha q polypeptide	0.813	-0.298	-1.776	cell communication; nervous system development;synapse
224792	Gpr116	G protein-coupled receptor 116	1.279	0.355	2.154	cell communication
14799	Gria1	glutamate receptor, ionotropic, AMPA1 (alpha 1)	0.776	-0.365	-1.975	synapse
14800	Gria2	glutamate receptor, ionotropic, AMPA2 (alpha 2)	0.740	-0.435	-1.907	cell communication; synapse
14804	Grid2	glutamate receptor, ionotropic, delta 2	0.806	-0.312	-1.793	synapse
14943	Gzmf	granzyme F	0.801	-0.321	-1.928	cell death
15258	Hipk2	homeodomain interacting protein kinase 2	0.761	-0.394	-2.283	cell communication; cell death
26557	Homer2	homer homolog 2 (Drosophila)	0.774	-0.37	-2.144	cell communication
14828	Hspa5	heat shock 70 kD protein 5 (glucose-regulated protein)	0.716	-0.481	-1.984	cell communication; cell death
56213	Htra1	HtrA serine peptidase 1	0.795	-0.331	-1.834	cell communication; receptor binding and activity
15951	Ifi204	interferon activated gene 204	1.268	0.343	2.104	cell death
16323	Inhba	inhibin beta-A	0.718	-0.477	-2.551	cell death; receptor binding and activity
241226	Itga8	integrin alpha 8	1.270	0.345	2.112	cell adhesion; cell communication
16419	Itgb5	integrin beta 5	0.832	-0.265	-1.649	cell adhesion; cell communication
16443	Itsn1	intersectin 1 (SH3 domain protein 1A)	0.826	-0.275	-1.839	cell communication
22343	Lin7c	lin-7 homolog C (C. elegans)	0.831	-0.267	-1.652	cell communication; synapse
330814	Lphn1	latrophilin 1	0.803	-0.316	-1.934	cell communication
16998	Ltbp3	latent transforming growth factor beta binding protein 3	1.291	0.368	2.202	cell communication; receptor binding and activity
17762	Mapt	microtubule-associated protein tau	0.727	-0.459	-2.312	nervous system development
17118	Marcks	myristoylated alanine rich protein kinase C substrate	0.799	-0.324	-1.815	receptor binding and activity
13728	Mark2	MAP/microtubule affinity-regulating kinase 2	0.817	-0.291	-1.922	cell communication
17193	Mbd4	methyl-CpG binding domain protein 4	0.818	-0.289	-1.753	cell death
52065	Mfhas1	malignant fibrous histiocytoma amplified sequence 1	0.759	-0.398	-2.297	cell communication
59030	Mkks	McKusick-Kaufman syndrome protein	0.749	-0.416	-2.427	cell communication
17346	Mknk1	MAP kinase-interacting serine/threonine kinase 1	1.273	0.348	2.113	cell communication
17748	Mt1	metallothionein 1	0.807	-0.31	-1.974	cell communication
17750	Mt2	metallothionein 2	0.780	-0.359	-2.141	cell communication
17909	Myo10	myosin X	0.824	-0.28	-1.751	cell communication
17918	Myo5a	myosin Va	1.309	0.389	2.105	cell communication; receptor binding and activity; synapse
17984	Ndn	necdin	1.315	0.395	2.209	cell communication; nervous system development
192167	Nlgn1	neuroligin 1	1.381	0.466	2.470	cell communication; nervous system development; synapse
18125	Nos1	nitric oxide synthase 1, neuronal	0.795	-0.331	-1.953	cell communication; receptor binding and activity; synapse
225872	Npas4	neuronal PAS domain protein 4	1.355	0.438	2.375	cell communication
18212	Ntrk2	neurotrophic tyrosine kinase, receptor, type 2	0.797	-0.327	-1.978	cell communication; synapse
18378	Omp	olfactory marker protein	0.790	-0.34	-1.907	cell communication
18389	Oprl1	opioid receptor-like 1	1.330	0.411	2.066	cell communication
18577	Pde4a	phosphodiesterase 4A, cAMP specific	0.812	-0.301	-1.903	cell communication
18578	Pde4b	phosphodiesterase 4B, cAMP specific	0.812	-0.301	-1.586	cell communication
18583	Pde7a	phosphodiesterase 7A	0.830	-0.268	-1.656	cell communication
14827	Pdia3	protein disulfide isomerase associated 3	0.812	-0.3	-1.829	cell death
74055	Plce1	phospholipase C, epsilon 1	0.807	-0.309	-1.923	cell communication
67916	Ppap2b	phosphatidic acid phosphatase type 2B	0.784	-0.351	-2.088	cell communication
170826	Ppargc1b	peroxisome proliferative activated receptor, gamma, coactivator 1 beta	0.749	-0.417	-2.262	cell communication
333654	Ppp1r13l	protein phosphatase 1, regulatory (inhibitor) subunit 13 like	0.820	-0.287	-1.903	cell death
73728	Psd	pleckstrin and Sec7 domain containing	1.291	0.368	2.130	cell communication
19246	Ptpn1	protein tyrosine phosphatase, non-receptor type 1	0.754	-0.407	-2.217	cell communication
19268	Ptprf	protein tyrosine phosphatase, receptor type, F	0.815	-0.296	-1.789	cell communication
19334	Rab22a	RAB22A, member RAS oncogene family	0.817	-0.292	-1.773	cell communication
19337	Rab33a	RAB33A, member of RAS oncogene family	1.276	0.352	2.165	cell communication
19340	Rab3d	RAB3D, member RAS oncogene family	0.792	-0.337	-1.964	cell communication
19415	Rasal1	RAS protein activator like 1 (GAP1 like)	1.312	0.392	2.203	cell communication
17252	Rdh11	retinol dehydrogenase 11	1.300	0.378	2.172	cell communication
56533	Rgs17	regulator of G-protein signaling 17	1.309	0.388	2.141	cell communication
56470	Rgs19	regulator of G-protein signaling 19	1.275	0.35	2.124	cell communication
19893	Rpgr	retinitis pigmentosa GTPase regulator	1.322	0.403	2.232	cell communication
77945	Rpgrip1	retinitis pigmentosa GTPase regulator interacting protein 1	0.784	-0.351	-1.946	cell communication
110876	Scn2a1	sodium channel, voltage-gated, type II, alpha 1	0.798	-0.325	-1.848	cell communication; cell death
58234	Shank3	SH3/ankyrin domain gene 3	0.779	-0.361	-2.189	cell communication; cell death
27401	Skp2	S-phase kinase-associated protein 2 (p45)	1.278	0.354	2.111	cell death
65962	Slc9a3r2	solute carrier family 9 (sodium/hydrogen exchanger), isoform 3 regulator 2	0.816	-0.293	-1.665	cell communication
17128	Smad4	MAD homolog 4 (Drosophila)	0.802	-0.318	-1.838	cell communication
20411	Sorbs1	sorbin and SH3 domain containing 1	0.779	-0.361	-1.938	cell adhesion; cell communication
20692	Sparc	secreted acidic cysteine rich glycoprotein	0.756	-0.403	-2.405	cell communication
114715	Spred1	sprouty protein with EVH-1 domain 1, related sequence	0.796	-0.329	-1.928	cell communication
114716	Spred2	sprouty-related, EVH1 domain containing 2	0.776	-0.365	-1.933	cell communication
14270	Srgap2	SLIT-ROBO Rho GTPase activating protein 2	0.825	-0.278	-1.778	cell communication
20848	Stat3	signal transducer and activator of transcription 3	0.808	-0.308	-1.710	cell communication
20913	Stxbp4	syntaxin binding protein 4	0.786	-0.347	-2.069	cell communication
240725	Sulf1	sulfatase 1	0.833	-0.264	-1.811	cell death
104015	Synj1	synaptojanin 1	1.366	0.45	2.207	cell communication; synapse
24071	Synj2bp	synaptojanin 2 binding protein	0.752	-0.411	-2.093	cell communication
21415	Tcf3	transcription factor 3	0.799	-0.323	-1.978	cell communication
21416	Tcf7l2	transcription factor 7-like 2, T-cell specific, HMG-box	1.352	0.435	2.231	cell communication
110595	Timp4	tissue inhibitor of metalloproteinase 4	0.797	-0.328	-1.646	cell communication; synapse
22031	Traf3	Tnf receptor-associated factor 3	0.812	-0.3	-1.800	cell communication; cell death
94090	Trim9	tripartite motif protein 9	0.759	-0.397	-1.905	cell communication; synapse
22421	Wnt7a	wingless-related MMTV integration site 7A	0.827	-0.274	-1.758	cell communication; nervous system development; synapse
78889	Wsb1	WD repeat and SOCS box-containing 1	1.288	0.365	2.069	cell communication
22627	Ywhae	tyrosine 3-monooxygenase/tryptophan 5-monooxygenase activation protein, epsilon polypeptide	1.319	0.399	2.098	cell communication
235320	Zbtb16	zinc finger and BTB domain containing 16	0.776	-0.366	-1.759	cell death

**Table 7:** Significantly regulated genes in double-transgenic dnADAM10/APP_[V717I] _mice in relation to mono-transgenic APP_[V717I] _mice

**Gene ID**	**Gene symbol**	**Description**	**Fold change**	**log ratio**	**d-value**	**Functional groups**
268860	Abat	4-aminobutyrate aminotransferase	1.503	0.588	3.791	cell communication; synapse
67269	Agtpbp1	ATP/GTP binding protein 1	1.397	0.482	3.311	cell communication; synapse
226548	Aph1a	anterior pharynx defective 1a homolog (C. elegans)	0.724	-0.466	-3.127	cell communication
11938	Atp2a2	ATPase, Ca++ transporting, cardiac muscle, slow twitch 2	1.456	0.542	3.900	cell communication
140494	Atp6v0a4	ATPase, H+ transporting, lysosomal V0 subunit A4	0.762	-0.392	-2.972	cell communication
12122	Bid	BH3 interacting domain death agonist	1.381	0.466	3.765	cell death
12293	Cacna2d1	calcium channel, voltage-dependent, alpha2/delta subunit 1	1.460	0.546	4.086	cell communication
20303	Ccl4	chemokine (C-C motif) ligand 4	0.755	-0.405	-2.995	receptor binding and activity
12772	Ccr2	chemokine (C-C motif) receptor 2	0.648	-0.625	-3.824	cell communication
12955	Cryab	crystallin, alpha B	0.711	-0.492	-3.095	cell communication
12977	Csf1	colony stimulating factor 1 (macrophage)	0.767	-0.383	-2.920	cell adhesion; cell communication; receptor binding and activity
56066	Cxcl11	chemokine (C-X-C motif) ligand 11	0.760	-0.395	-2.953	receptor binding and activity
20315	Cxcl12	chemokine (C-X-C motif) ligand 12	1.373	0.457	3.359	receptor binding and activity
224997	Dlgap1	discs, large (Drosophila) homolog-associated protein 1	1.417	0.503	3.720	cell communication; synapse
13527	Dtna	dystrobrevin alpha	1.338	0.42	3.294	synapse
23882	Gadd45g	growth arrest and DNA-damage-inducible 45 gamma	1.371	0.455	3.534	cell death
14943	Gzmf	granzyme F	0.745	-0.424	-3.031	cell death
215114	Hip1	huntingtin interacting protein 1	0.772	-0.373	-2.971	cell death
15257	Hipk1	homeodomain interacting protein kinase 1	0.726	-0.461	-3.122	cell death
15452	Hprt1	hypoxanthine guanine phosphoribosyl transferase 1	1.375	0.459	3.326	cell communication; cell death; synapse
215257	Il1f9	interleukin 1 family, member 9	0.706	-0.503	-3.808	receptor binding and activity
16323	Inhba	inhibin beta-A	0.744	-0.426	-2.892	cell death; receptor binding and activity
16325	Inhbc	inhibin beta-C	0.715	-0.483	-3.508	receptor binding and activity
16179	Irak1	interleukin-1 receptor-associated kinase 1	1.431	0.517	3.661	receptor binding and activity
80782	Klrb1d	killer cell lectin-like receptor subfamily B member 1D	0.735	-0.444	-3.340	cell death
16818	Lck	lymphocyte protein tyrosine kinase	0.698	-0.519	-3.204	cell communication; cell death
17248	Mdm4	transformed mouse 3T3 cell double minute 4	1.377	0.462	3.303	cell death
59030	Mkks	McKusick-Kaufman syndrome protein	0.723	-0.468	-3.503	cell communication
17910	Myo15	myosin XV	0.753	-0.41	-2.883	cell communication
18125	Nos1	nitric oxide synthase 1, neuronal	0.741	-0.432	-3.040	cell communication; receptor binding and activity; synapse
21907	Nr2e1	nuclear receptor subfamily 2, group E, member 1	0.702	-0.511	-3.426	cell communication; cell death
57270	Olfr1508	olfactory receptor 1508	0.735	-0.444	-3.162	cell communication
18378	Omp	olfactory marker protein	0.756	-0.403	-2.860	cell communication
170677	Pcdh21	protocadherin 21	0.614	-0.703	-3.902	cell communication
14827	Pdia3	protein disulfide isomerase associated 3	0.745	-0.424	-2.866	cell death
18821	Pln	phospholamban	0.741	-0.432	-3.181	cell communication
333654	Ppp1r13l	protein phosphatase 1, regulatory (inhibitor) subunit 13 like	0.733	-0.449	-3.361	cell death
54189	Rabep1	rabaptin, RAB GTPase binding effector protein 1	1.377	0.462	3.307	cell death; receptor binding and activity
17252	Rdh11	retinol dehydrogenase 11	1.374	0.458	3.317	cell communication
212541	Rho	rhodopsin	0.762	-0.393	-3.078	cell communication
19877	Rock1	Rho-associated coiled-coil containing protein kinase 1	1.402	0.487	3.841	cell death
19893	Rpgr	retinitis pigmentosa GTPase regulator	1.361	0.445	3.332	cell communication
110876	Scn2a1	sodium channel, voltage-gated, type II, alpha 1	1.393	0.478	3.500	cell communication; cell death
58234	Shank3	SH3/ankyrin domain gene 3	0.769	-0.379	-2.946	cell communication; cell death
22293	Slc45a2	solute carrier family 45, member 2	0.731	-0.453	-3.211	cell communication
20682	Sox9	SRY-box containing gene 9	0.754	-0.408	-3.089	cell death
20977	Syp	synaptophysin	1.448	0.534	3.608	cell communication; synapse
20979	Syt1	synaptotagmin I	1.397	0.482	3.587	cell communication; synapse
21823	Th	tyrosine hydroxylase	0.761	-0.394	-2.993	cell communication; synapse
94090	Trim9	tripartite motif protein 9	0.716	-0.481	-2.963	cell communication; synapse
59025	Usp14	ubiquitin specific peptidase 14	1.383	0.468	3.342	cell communication; synapse
16963	Xcl1	chemokine (C motif) ligand 1	0.749	-0.416	-3.003	receptor binding and activity

The highest number of regulated genes in ADAM10 mono-transgenic mice (Tab. 4) belonged to the category of cell communication (53 genes), followed by the categories of synaptic junction and transmission (16 genes), and of nervous system development (15 genes). In dnADAM10 mice, fewer genes were found especially in the category of cell communication (15 genes).

In mono-transgenic mice, genes in the functional groups of inflammation or cell death were not over-represented (z-score < 1.96). In contrast, the category of cell death was over-represented in both double-transgenic mouse lines (Tab. 6 and 7), probably due to APP_[V717I] _overexpression.

The major difference in the two double-transgenic lines was the 3-fold higher number of regulated genes in the category of cell communication in the ADAM10/APP_[V717I] _double-transgenic line (96 genes), as compared to dnADAM10/APP_[V717I] _mice.

The results show that overexpression of proteolytically active ADAM10 generally influences cellular communication in mice, independently of their genetic background. One example for a regulated gene of this category is the calcium/calmodulin-dependent protein kinase II alpha (Camk2α), which is upregulated in mono-transgenic ADAM10 mice (Tab. 4) and downregulated in dnADAM10 mice (Tab. 5). Other genes of this category are the LDL receptor-related protein (Lrp1, Tab. 4, Additional file 1, Table S1), neuroligin (Nlgn1, Tab. 4, 6) and the very low density lipoprotein receptor (Vldlr, Additional file 1, Table S3).

ADAM10 overexpression has been shown to increase cortical synaptogenesis as revealed by immunohistochemistry [14]. Accordingly, here we confirmed these results on the mRNA level for two neurotransmitter systems: the glutamate receptor Gria3 and the glutamic acid decarboxylase 2 (Gad2) as well as the GABA-A receptor subunit alpha 4 (Gabra4). These are examples of up-regulated genes within the category of synaptic junction and transmission (Tab. 4).

Because ADAM10 has proteolytical activity, we were also interested in gene expression of putative ADAM10 substrates like APP and Egfr (Tab. 8). Their expressions were not regulated in mono-transgenic mice, and therefore they are not listed in tables 4, 5, 6, 7 and tables S1-S4.

**Table 8:** Substrates of ADAM10 [43] which are not regulated in mono-transgenic mice within the parameters given in the Methods section

**Substrate groups**	**Symbol name of Mouse Gene**
CNS substrates of ADAM10	App, Aplp2, Prnp, Efna2, L1cam, Cdh2, Pcdhg, Dll1, Notch1

Substrates of ADAM10 in inflammation	Cx3cl1, Cxcl16, Cdh5, F11r (JAM-A), Il6r, Fasl, Tnfrsf8 (CD30), Cd44

Growth factors and receptors cleaved by ADAM10	Egfr, Egf, Btc, Erbb2

**Figure 4:**
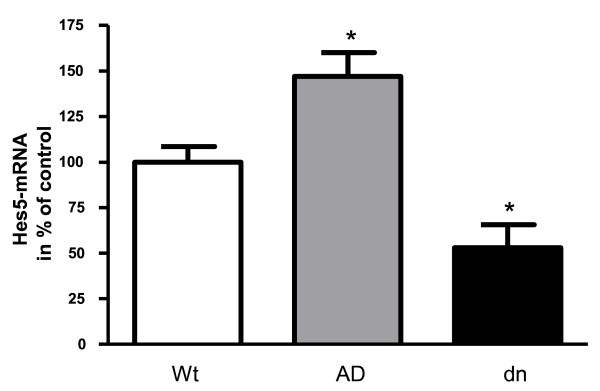
**Hes5 mRNA levels in 15 days old ADAM10 transgenic mice**. Brains of 15 days old FVB/N (Wt), ADAM10 (AD) or ADAM10 dominant negative mutant (dn) overexpressing mice were analyzed for the amount of the Notch-1 target gene Hes5 mRNA. Quantification was performed by real-time RT-PCR. Values represent means ± SEM of four mice per group normalized to GAPDH mRNA (one way ANOVA, Dunnett's Multiple Comparison Test; p < 0.05, *).

Notch-1 expression was not changed in mice aged 5 months and its target gene Hes5 was only slightly affected in ADAM10 mice (Tab. 4). However, it has been reported that the ADAM10 knock out leads to severely affected Notch signaling and embryonic lethality at day 9.5 [11]. As in our transgenic animals ADAM10 was under control of the postnatal active neuron-specific mouse Thy 1-promoter, ADAM10 has no effect during embryogenesis. To examine whether the lack of influence of ADAM10 on the Notch pathway in our transgenic mice is due to the relative late stage (5 months) of investigation, we analyzed the expression of the Notch-1 target gene Hes5 in transgenic mice aged 15 days (Fig. 4): about 40% induction was observed in the ADAM10 overexpressing mice and a reduction of about 50% in the dnADAM10 transgenic mice.

In addition, we found that overexpression of ADAM10 and dnADAM10 did not affect expression of either endogenous Adam10 or of other putative α-secretases like Adam9, Adam17 and Bace2 in adult mice. In general, the observed alteration of gene expression was low in all analyzed mouse lines (see the fold changes in Tab. 4, 5, 6, 7).

### Alzheimer disease-related genes regulated by ADAM10

The GeneCards database (Weizmann Institute of Science, Version 2.36), which contains 934 genes connected with AD (gene list see Additional file 1, Tab. S7), was used for identification of AD-related genes regulated by ADAM10.

**Figure 5:**
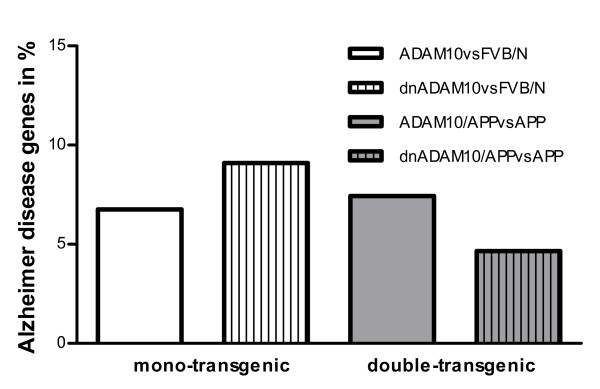
**Alzheimer disease genes in mono- and double-transgenic mice**. Differentially expressed genes of ADAM10 versus FVB/N with FDR 0.5 (355 genes), of dnADAM10 versus FVB/N with FDR 0.5 (143 genes), of ADAM10/APP_[V717I] _versus APP_[V717I] _with FDR 0.0 (592 genes) and of dnADAM10/APP_[V717I] _versus APP_[V717I] _with FDR 0.0, restricted to 300 up- and 300 downregulated genes (600 genes), were compared to AD genes (934 genes) from GeneCards (Weizmann Institute of Science, Version 2.36). In ADAM10 versus FVB/N 25 AD genes (7% of 355 genes), in dnADAM10 versus FVB/N 13 AD genes (9% of 143 genes), in ADAM10/APP_[V717I]_versus APP_[V717I] _43 AD genes (7% of 592 genes) and in dnADAM10/APP_[V717I] _versus APP_[V717I] _30 AD genes (5% of 600 genes) were found.

In ADAM10 mice, 25 AD genes (7% of 355 genes) were differently regulated, and in dnADAM10 mice 13 AD genes (9% of 143 genes) (Fig. 5) were altered including genes involved in cholesterol and lipid homeostasis, like Lrp1, Vldlr, and fatty acid-binding protein Fabp7. Other regulated genes code for inflammation-associated members of the S100 protein family (S100a8 and S100a9).

In ADAM10/APP_[V717I] _mice, 43 AD genes (7% of 592 genes), and in dnADAM10/APP_[V717I] _mice, 30 AD genes (5% of 600 genes) were altered in expression (Fig. 5). The relatively small number of ADAM10-regulated AD genes in double-transgenic mice probably reflects brain dissection at the age of five months, before plaque formation begins. In all transgenic lines, we did not detect differences in the expression of presenilins 1 and 2. Bace1 was slightly upregulated (25%) in dnADAM10/APP_[V717I] _mice. The Aβ-degrading enzymes neprilysin (Mme) and insulin-degrading enzyme (Ide) were also not regulated in mono-transgenic mice. Solely, in ADAM10/APP_[V717I] _mice, Ide was slightly down-regulated (Additional file 1, Table S3).

**Figure 6:**
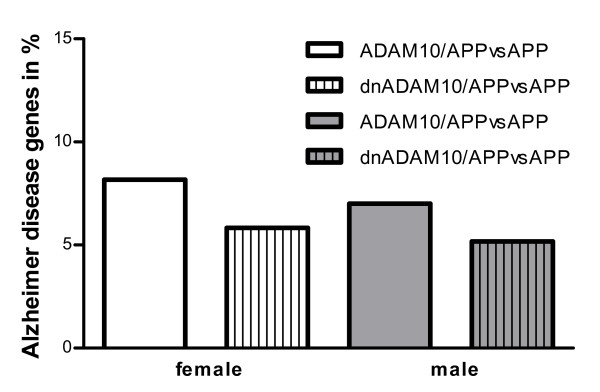
**Alzheimer disease genes in female and male double-transgenic mice**. Differentially expressed genes of female and male ADAM10/APP_[V717I] _versus APP_[V717I] _and of dnADAM10/APP_[V717I]_versus APP_[V717I] _with FDR 1.3, restricted to the 600 best up- and downregulated genes were analyzed for AD genes (934 genes) from GeneCards (Weizmann Institute of Science, Version 2.36). In female ADAM10/APP_[V717I] _versus APP_[V717I] _mice 49 AD genes (8% of 600 genes) and in female dnADAM10/APP_[V717I] _versus APP_[V717I] _animals 35 AD genes (6% of 600 genes) were found to be affected. In male ADAM10/APP_[V717I] _versus APP_[V717I] _mice 42 AD genes (7% of 600 genes) and in male dnADAM10/APP_[V717I] _versus APP_[V717I] _mice 31 AD genes (5% of 600 genes) were found to be affected. The corresponding d-values are listed separately for male (Tab. 9) and female mice (Tab. 10).

In order to examine an influence of sex, a separate ChipInspector analysis restricted to the 600 most up- and downregulated genes was performed with samples from both female and male double-transgenic mice (Tab. 9, 10). The gene lists of female and male double-transgenic mice were then compared to the GeneCards AD gene list. The percentages of altered AD-related genes in female double-transgenic ADAM10/APP_[V717I] _and dnADAM10/APP_[V717I]_female mice are similar to the numbers found in male ADAM10/APP_[V717I]_and dnADAM10/APP_[V717I] _mice (Fig. 6). Thus, sexual dimorphism does not cause severe differences in ADAM10-dependent expression of AD-related genes. One exception was the insulin-like growth factor (Igf1), which was downregulated in female dnADAM10/APP_[V717I] _mice (0.65; FDR = 1.3%), but not in the corresponding male animals (1.17; FDR = 1.8).

**Table 9:** Numbers of significantly regulated genes in male double-transgenic mice restricted by the given d-values.

**Mouse Genome 430 2.0 Array (Affymetrix)**			
45 000 probe sets, 39 000 transcripts, 34 000 characterized			
male ADAM10/APP_[V717I] _versus APP_[V717I]_ 600 genes, FDR = 1.3%		male dnADAM10/APP_[V717I] _versus APP_[V717I]_ 600 genes, FDR = 1.3%	

414 upregulated (d-value > 1.18)	186 downregulated (d-value < -0.71)	320 upregulated (d-value > 1.29)	280 downregulated (d-value < -0.75)

**Table 10:** Numbers of significantly regulated genes in female double-transgenic mice restricted by the given d-values

**Mouse Genome 430 2.0 Array (Affymetrix)**			
45 000 probe sets, 39 000 transcripts, 34 000 characterized			
female ADAM10/APP_[V717I] _versus APP_[V717I]_ 600 genes, FDR = 1.3%		female dnADAM10/APP_[V717I] _versus APP_[V717I]_ 600 genes, FDR = 1.3%	

184 upregulated (d-value > 0.61)	416 downregulated (d-value < -0.66)	300 upregulated (d-value > 1.44)	300 downregulated (d-value < -1.38)

### Genes regulated through APP_[V717I] _overexpression

**Figure 7:**
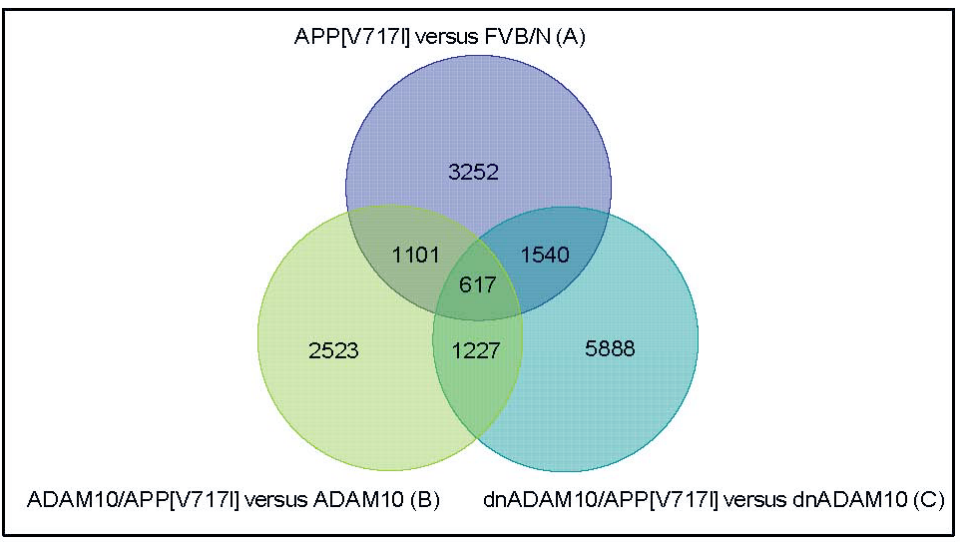
**Venn diagram of regulated genes in investigated mouse lines after CARMA-analysis (BH<0.005)**. Venn diagram of APP_[V717I]_versus FVB/N (A), ADAM10/APP_[V717I] _versus ADAM10 (B) und dnADAM10/APP_[V717I] _versus dnADAM10 (C), generated by a custom-written Perl-script showing the effect of APP_[V717I] _overexpression in double-transgenic mice. The numbers in the spaces of overlapping circles represent the number of transcripts that were affected in all mouse groups. The numbers in the outer portion of each circle represent the number of transcripts that were exclusively affected in two mouse groups.

To determine the effect of APP_[V717I] _overexpression on gene regulation in transgenic mice, we compared APP_[V717I] _mice with FVB/N mice, ADAM10/APP_[V717I] _mice with ADAM10 mice, and dnADAM10/APP_[V717I] _mice with dnADAM10 mice. After background adjustment and normalization with the GCRMA procedure, a Venn diagram of genes regulated in the transgenic mice was generated (Fig. 7). The overlap of the three groups represents 617 genes regulated by APP_[V717I] _overexpression, independent of the strain background. This high number of genes altered by APP_[V717I] _expression demonstrates the strong influence of human APP_[V717I] _overexpression in the AD mouse model used.

AD-related genes that were regulated in double-transgenic, but not in mono-transgenic mice include microtubule-associated protein tau (Mapt) (Tab. 6; Tab. S3) and the ionotropic glutamate receptors AMPA 1 (Gria 1) and AMPA 2 (Gria 2) (Tab. 6; Tab. S3).

### Confirmation of microarray data

**Figure 8:**
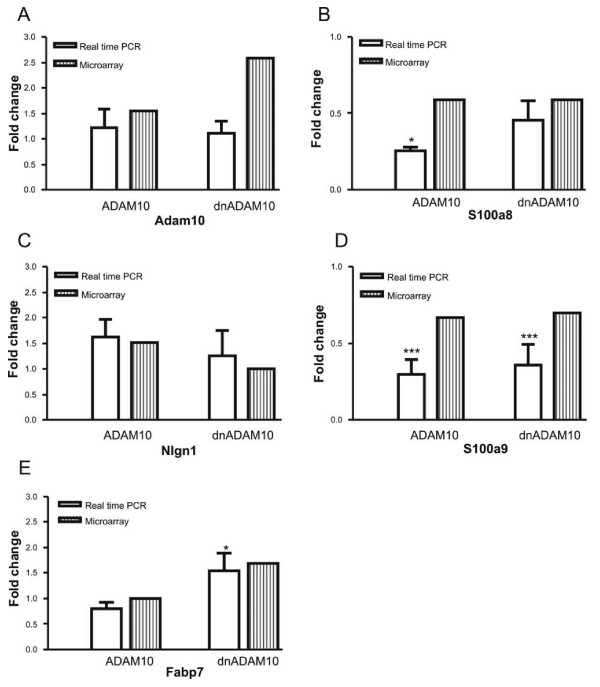
**Analyses of gene expression of selected candidate genes by real-time RT-PCR in mono-transgenic mice**. Expression levels of individual genes in mono-transgenic mice in relation to gene expression in FVB/N wild-type mice. Shown are the results from RT-PCR and microarray analyses. Values presented: mean of fold changes ± SD of three animals. A: ADAM10; B: S100a8; C: Nlgn1; D: S100a9; E: Fabp7. Statistical significance was determined by using ANOVA analysis, followed by Dunnett's post hoc comparison (*), p ≤ 0.05; (**), p ≤ 0.001; (***), p ≤ 0.001.

**Figure 9:**
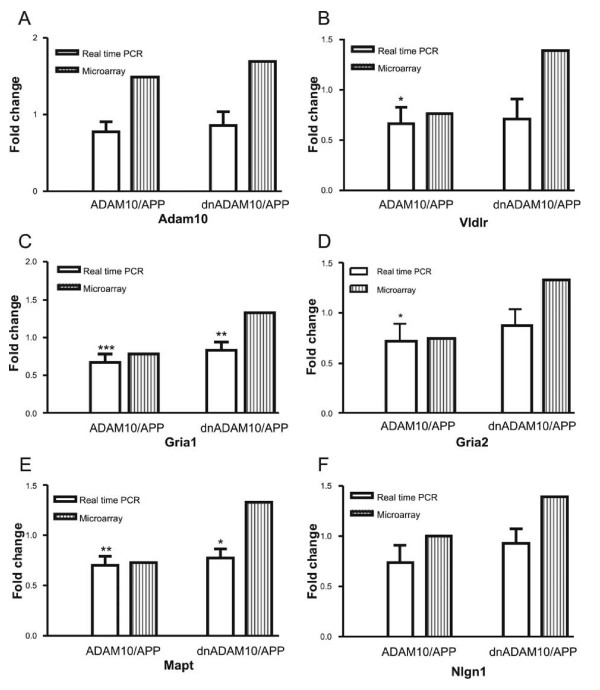
**Analyses of gene expression of selected candidate genes by real-time RT-PCR in double-transgenic mice**. Expression levels of individual genes in double-transgenic mice in relation to gene expression in APP_[V717I] _mono-transgenic mice. Shown are the results from RT-PCR and microarray analyses. Values presented: mean of fold changes ± SD of 4–6 animals. A: ADAM10; B: Vldlr; C: Gria1; D: Gria2; E: Mapt; F: Nlgn1. Statistical significance was determined by using ANOVA analysis, followed by Dunnett's post hoc comparison (*), p ≤ 0.05; (**), p ≤ 0.001; (***), p ≤ 0.001.

For validation of the results obtained by microarray analysis, real-time RT-PCR was applied on the original RNA samples (Fig. 8 and 9). Changes in gene expression levels of selected transcripts were normalized to the gene expression of GAPDH, which was not regulated in our transgenic mouse strains.

**Figure 10:**
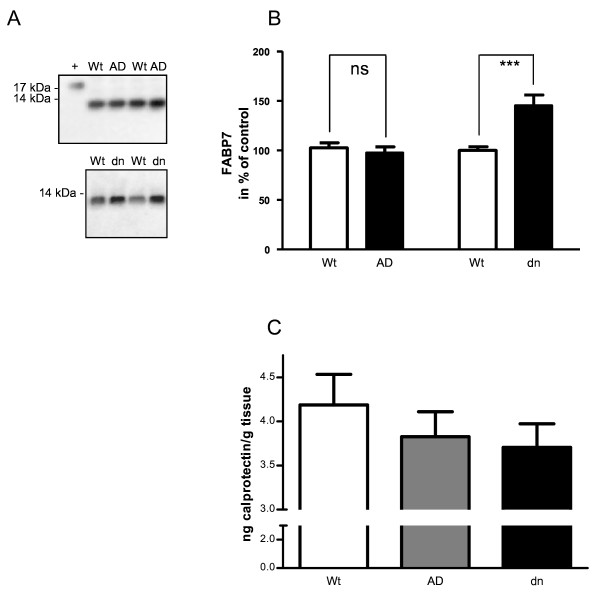
**Effect of Adam10 on Fabp7 and S100a8/a9 proteins in mouse brain**. A) Fabp7 protein expression was analyzed in fractions of soluble brain proteins of mono-transgenic mice by Western blotting (Wt: wild-type, AD: ADAM10, dn: dominant negative ADAM10). As a control for antibody specificity, a lysate of HEK293 cells overexpressing V5-tagged murine Fabp7 (19 kDa; +) was used. B) Quantification of Fabp7 was performed with at least 5 animals per group. Values represent mean ± SEM, and values obtained for wild-type animals (Wt) were set to 100% (one way ANOVA, Bonferroni post-test; ns, not significant; p < 0.001, ***). C) Expression of dimeric S100a8/a9 (calprotectin) was quantified by ELISA in mouse brain extracts. Measured absorptions at 405 nm were normalized to wet tissue weight (mean ± SEM; n = 4).

In the microarray analyses, the calcium-binding proteins (S100a8 and S100a9) were found to be downregulated in ADAM10 and dnADAM10 mice. Both genes are associated with various inflammatory processes including Alzheimer's disease [25]. By using real-time RT-PCR, significant downregulation of S100a8 and S100a9 was confirmed (Fig. 8B and 9D). Additionally, quantification of dimers of S100a8 and a9 (calprotectin) by ELISA revealed a slight reduction in both transgenic mouse lines (Fig. 10C) which is in accordance with the findings for mRNA levels. The decrease of about 10 to 15% of calprotectin as compared to wild-type mice was not statistically significant which might be due to ELISA-specific detection of heterodimers. We cannot exclude that changes concerning both monomeric proteins might be more substantial, but a detection of the monomeric form of S100a9 by Western blotting failed as a consequence of its low expression level.

Fatty acid-binding protein 7 (Fabp7), which is elevated in Down syndrome fetal brains [26], was found to be upregulated in dnADAM10 mice by microarray analysis. A significantly increased Fabp7 expression was confirmed in dnADAM10 mice by real-time RT-PCR. As observed by real-time RT-PCR, expression of Fabp7 was slightly reduced in ADAM10 mice, but this effect did not reach a significant level (Fig. 8E). Fabp7 protein expression was analyzed in the soluble protein fraction from brains of mono-transgenic mice by Western blotting. While ADAM10 had no significant effect on Fabp7 expression, the dominant-negative form dnADAM10 increased the amount of the Fabp7 protein (Fig. 10A/B), which is in accordance with the results obtained by microarray and PCR analysis.

Neuroligin 1, a postsynaptic cell-adhesion molecule of excitatory synapses, plays a role in neuronal differentiation and axogenesis [27]. In microarray analyses, neuroligin 1 gene expression was induced in ADAM10 mice. This tendency, although without reaching significance, was also confirmed in real-time RT-PCR (Fig. 8C).

Other proteins identified by gene profiling and associated with Alzheimer disease are the low density lipoprotein receptor-related protein 1 (Lrp1) [28], the very low density lipoprotein receptor (Vldlr) [29], the microtubule-associated protein tau (Mapt) [30] and the ionotropic glutamate receptors AMPA1 and AMPA2 (Gria1 and Gria2) [31]. Downregulation of Lrp1 by ADAM10, as observed in the chip analyses, was not confirmed by real-time RT-PCR (results not shown). For Vldlr, we found by real-time RT-PCR a significant downregulation in ADAM10/APP_[V717I] _mice, but its upregulation in dnADAM10/APP_[V717I] _mice, as detected with the microarray, could not be confirmed (Fig. 9B).

By real-time RT-PCR, the microtubule-associated protein tau was shown to be significantly downregulated in both double-transgenic mouse lines (Fig. 9E). Also in the case of the ionotropic glutamate receptors AMPA1 (Gria1) and AMPA2 (Gria 2), real-time RT-PCR confirmed the results of the microarray analyses: both genes are downregulated in ADAM10/APP_[V717I] _mice (Fig. 9C and 9D).

## Discussion

Increasing the α-secretase cleavage of APP represents a plausible strategy for the treatment of Alzheimer disease, because via this route it is possible to decrease the concentration of neurotoxic Aβ peptides and to increase the amount of neuroprotective APPsα simultaneously.

The aim of this study was to investigate the influence of increased amounts of ADAM10 proteins on gene expression in the mouse CNS. To this end, we analyzed transgenic mice either overexpressing catalytically active ADAM10, or a dominant-negative mutant of ADAM10 (dnADAM10) which is able to inhibit endogenous mouse enzymes with α-secretase activity [10,12]. An additional reason for investigation of dnADAM10 mice is determined by the multi-domain structure of ADAMs because specific biological functions have been assigned to protein domains outside the catalytic centre of ADAMs [15].

In ADAM10 mice, more genes were regulated than in dnADAM10 animals; this indicates that, due to the many substrates of ADAM10, an increase in their cleavage products might change the expression of genes involved in cell communication and synaptic transmission. No change, however, was detected in the expression of the substrates as a feedback reaction.

In all transgenic mice the endogenous ADAM10 level was not influenced through overexpression of ADAM10 or its inactive variant as revealed by real-time RT-PCR. Also the other ADAM family members Adam9 and Adam17/TACE were not regulated differentially in the investigated transgenic mice, thus indicating that a reduced α-secretase activity as observed in dnADAM10 mice [12] was not compensated by the induction of gene expression of other potential α-secretases.

Since ADAM10 has been implicated in Notch signaling [11,32], we investigated this pathway. On the RNA level, we found no regulation of Notch-1 in mono- and double-transgenic mice at the age of 5 months: expression of the Notch target gene Hes5 was only slightly changed in mono-transgenic ADAM10 mice. This is in accordance with earlier real-time RT-PCR experiments, where no significant difference was found in Hes5 transcription levels between adult mice overexpressing ADAM10 and non-transgenic mice [12]. This lack of influence on Notch signaling is probably due to the late stage of analysis, since we found small but significant effects of ADAM10 on Hes5 mRNA levels in transgenic mice aged 15 days.

ADAM10 has been reported to mediate cadherin shedding, β-catenin translocation and expression of β-catenin target genes [33,34]. In double-transgenic dnADAM10/APP_[V717I] _mice various cadherins (Cdh8, Cdh10 and Cdh13), β-catenin (Ctnnb1), several Wnts (Wnt4, Wnt7a and Wnt9a) and Jun kinase (Jun) were upregulated (about 30%). The upregulation of these genes might represent a compensatory mechanism to by-pass a reduced catalytic activity of ADAM10 and β-catenin signaling. In mice overexpressing active ADAM10, no significant changes of β-catenin target genes, for example c-myc and cyclin D1, were found.

Also for other ADAM10 substrates like L1cam, proteins involved in inflammation like Fasl, and for growth factor receptors like Egfr (see also table 8), we could not demonstrate any alteration.

Most genes in ADAM10 and ADAM10/APP_[V717I] _mice were found to be altered in the pathway of cell communication, followed by genes in categories of nervous system development and synaptic junction and transmission (Tab. 4, 5, 6, 7). One example for a regulated gene in the category of cell communication and synaptic function is the calcium/calmodulin-dependent protein kinase II alpha (Camk2α), one of the most abundant kinases in the brain, which is involved in long term potentiation. Camk2α was upregulated in ADAM10 mice, and downregulated in dnADAM10. Another gene of cell communication and synaptic function is neuroligin (Nlgn1), a brain-specific acetylcholinesterase homologous protein, which was upregulated in ADAM10/APP_[V717I] _mice (Fig. 9F). This component of excitatory synapses plays a role in neuronal differentiation and axogenesis [27]. An increase in cortical synaptogenesis as found by Bell et al. in ADAM10 mice [14], was confirmed through upregulation of the glutamate receptor Gria3 and the glutamic acid decarboxylase 2 (Gad2) as well as the GABA-A receptor subunit alpha 4 (Gabra4).

Downregulation of the ionotropic glutamate receptors AMPA1 (Gria1) and AMPA2 (Gria2) as observed in our microarray study was confirmed by real-time RT-PCR: reduced mRNA levels of Gria1 and Gria2 were detected in ADAM10/APP_[V717I] _mice. The downregulation of these two genes possibly depends on overexpression of APP_[V717I] _as described before [35,36].

The number of regulated genes involved in the development of AD was relatively small in the brains of double-transgenic ADAM10/APP_[V717I] _and dnADAM10/APP_[V717I] _mice, and almost equivalent to mono-transgenic ADAM10 or dnADAM10 mice (Fig. 5). We did not detect differences in most genes directly involved in APP processing; but reduction of α-secretase activity induced a slight upregulation of Bace1 in dnADAM10/APP_[V717I] _mice.

Comparative GCRMA analysis demonstrated the strong influence of human APP_[V717I] _overexpression on gene expression in double-transgenic mice. Tau (Mapt) was directly downregulated through APP_[V717I] _overexpression in ADAM10/APP_[V717I] _versus ADAM10 mice. Altered expression of AD-related genes was independent of sex, with one exception: insulin-like growth factor 1 (Igf-1), which has been implicated in Alzheimer pathology [37,38], was downregulated in double-transgenic female dnADAM10/APP_[V717I] _mice.

By microarray analysis, we observed in mono-transgenic mice a downregulation of members of the S100 protein family, small calcium-binding proteins responsible for a wide range of intra- and extracellular functions [39]. S100a8 and S100a9 were expressed to a lower extent in ADAM10 and dnADAM10 mice. PCR analysis and ELISA confirmed this effect (Fig. 8B and 8D, Figure 10C). S100a8 and S100a9 form the dimer calprotectin which is a marker for inflammation [40]. Immunohistochemical analysis recently showed S100A9 in association with the neuropathological hallmarks of sporadic and familiar AD: it was found in senile plaques, in activated glia cells and in neurons with neurofibrillary tangle morphology [25]. The downregulation of S100a9 by both ADAM10 and dnADAM10 overexpression is probably mediated by their common domains (the disintegrin and cystein-rich domain as well as the C-terminus).

A member of the fatty acid-binding proteins (Fabp7) was regulated by ADAM10 in mono-transgenic mice. Fabp7, also named brain lipid-binding protein (B-Fabp), is localized in the cytoplasm and in the nucleus, and is involved in the uptake, storage and/or delivery of fatty acids and retinoids into the nucleus [41]. Fabp7 is mainly expressed in radial glial cells, and is necessary for proper migration of immature neurons to cortical layers. Increased amounts of Fabp7 in the brains of individuals with Down syndrome suggest that higher concentrations of Fabp7 contribute to brain abnormalities and mental retardation [26]. We observed a significant upregulation of Fabp7 mRNA and protein in dnADAM10 mice. Since in Down syndrome patients α-secretase activity significantly decreases with age [42], our results provide a connection between inhibition of α-secretase (in our study by dnADAM10) and upregulation of Fabp7.

## Conclusion

This study shows that overexpression of ADAM10 or dnADAM10 in the brain of adult mice does not lead to drastic alteration of gene expression. In particular, ADAM10 or dnADAM10 overexpression does not result in an increased expression of genes coding for pro-inflammatory or pro-apoptotic proteins. On the contrary, overexpression of ADAM10 and its mutant even leads to a decreased amount of the inflammation marker calprotectin (the dimer of S100a8 and S100a9).

The relatively low number of genes affected by the ADAM10 modulation and the mild characteristic of altered expression levels might be related to the age of the mice we investigated. Since expression in the whole brain was analyzed, a higher change of gene expression may occur in single areas like the hippocampus. From other reports it is evident that manipulation of ADAM10 in embryonic or early ontogenic stages could have severe side effects but therapeutic approaches concerning Alzheimer's disease always will focus on adult patients. Our results in sum therefore provide evidence that, due to its effect on inflammation markers and on Fabp7 expression, ADAM10 might have beneficial effects in addition to those that are due to its α-secretase activity. These results further support the strategy of ADAM10 upregulation as a therapeutic approach for the treatment of AD.

## Abbreviations

(AD): Alzheimer disease; (APP): amyloid precursor protein; (Aβ peptides): Amyloid β-peptides; (ADAM10): a disintegrin and metalloproteinase 10.

## Authors' contributions

CP has carried out all molecular genetic experiments and has drafted the manuscript. DT has performed the analyses of biological pathways and contributed to the manuscript draft; WW has coordinated the bioinformatic analysis. KE has performed the Western blot analysis, the ELISA, quantification of Hes5-mRNA and co-drafted the manuscript; RP has participated in the design of the study and co-drafted the manuscript. FF has conceived and coordinated the study, drafted the final version of the manuscript and given approval for its publication.

## Supplementary Material

Additional file 1 **Additional tables including differentially regulated genes in ADAM10 and mutant ADAM10 transgenic mice.** Table S1 Complete list of significantly regulated genes in mono-transgenic ADAM10 mice (three females 5 months old ADAM10 mice as well as FVB/N wild-type mice). Table S2 Complete list of significantly regulated genes in mono-transgenic dnADAM10 mice (three females 5 months old, dnADAM10 mice as well as FVB/N wild-type mice). Table S3 Complete list of significantly regulated genes in double-transgenic ADAM10/APP[V717I] mice (three female and three male 5 months old, ADAM10/APP[V717I] mice as well as APP[V717I] mice). Table S4 Complete list of significantly regulated genes in double-transgenic dnADAM10/APP[V717I] mice (three female and three male 5 months old, dnADAM10/APP[V717I] mice as well as APP[V717I] mice). Table S5 Commonly regulated genes through ADAM10 overexpression in mono- and double transgenic mice (ADAM10 versus FVB/N (355 genes) compared to ADAM10/APP[V717I] versus APP[V717I] (592 genes). Table S6 Commonly regulated genes through dnADAM10 overexpression in mono- and double transgenic mice (dnADAM10 versus FVB/N (143 genes) compared to dnADAM10/APP[V717I] versus APP[V717I] (600 genes)). Table S7 934 Alzheimer disease genes by GeneCards (Weizmann Institute of Science, Version 2.36) Click here for file
