# Supplementary material for: Differential gene expression in ADAM10 and mutant ADAM10 transgenic mice
Source: BMC Genomics. 2009 Feb 5;10:66. doi: 10.1186/1471-2164-10-66 (PMC2647556; doi:10.1186/1471-2164-10-66)
Supplement: Additional file 1 — Additional tables including differentially regulated genes in ADAM10 and mutant ADAM10 transgenic mice. Table S1 Complete list of significantly regulated genes in mono-transgenic ADAM10 mice (three females 5 months old ADAM10 mice as well as FVB/N wild-type mice). Table S2 Complete list of significantly regulated genes in mono-transgenic dnADAM10 mice (three females 5 months old, dnADAM10 mice as well as FVB/N wild-type mice). Table S3 Complete list of significantly regulated genes in double-transgenic ADAM10/APP[V717I] mice (three female and three male 5 months old, ADAM10/APP[V717I] mice as well as APP[V717I] mice). Table S4 Complete list of significantly regulated genes in double-transgenic dnADAM10/APP[V717I] mice (three female and three male 5 months old, dnADAM10/APP[V717I] mice as well as APP[V717I] mice). Table S5 Commonly regulated genes through ADAM10 overexpression in mono- and double transgenic mice (ADAM10 versus FVB/N (355 genes) compared to ADAM10/APP[V717I] versus APP[V717I] (592 genes). Table S6 Commonly regulated genes through dnADAM10 overexpression in mono- and double transgenic mice (dnADAM10 versus FVB/N (143 genes) compared to dnADAM10/APP[V717I] versus APP[V717I] (600 genes)). Table S7 934 Alzheimer disease genes by GeneCards (Weizmann Institute of Science, Version 2.36) [file 1471-2164-10-66-S1.doc]

**Additional file 1**

Table S1 Complete list of significantly regulated genes in mono-transgenic ADAM10 mice (three females 5 months old ADAM10 mice as well as FVB/N wild-type mice)

Table S2 Complete list of significantly regulated genes in mono-transgenic dnADAM10 mice (three females 5 months old, dnADAM10 mice as well as FVB/N wild-type mice)

Table S3 Complete list of significantly regulated genes in double-transgenic ADAM10/APP_[V717I]_ mice (three female and three male 5 months old, ADAM10/APP_[V717I]_ mice as well as APP_[V717I]_ mice)

Table S4 Complete list of significantly regulated genes in double-transgenic dnADAM10/APP_[V717I]_ mice (three female and three male 5 months old, dnADAM10/APP_[V717I]_ mice as well as APP_[V717I]_ mice)

Table S5 Commonly regulated genes through ADAM10 overexpression in mono- and double transgenic mice (ADAM10 versus FVB/N (355 genes) compared to ADAM10/APP_[V717I]_ versus APP_[V717I]_ (592 genes)

Table S6 Commonly regulated genes through dnADAM10 overexpression in mono- and double transgenic mice (dnADAM10 versus FVB/N (143 genes) compared to dnADAM10/APP_[V717I]_ versus APP_[V717I]_ (600 genes))

Table S7 934 Alzheimer disease genes by GeneCards (Weizmann Institute of Science, Version 2.36)

Table S1 Complete list of significantly regulated genes in mono-transgenic ADAM10 mice (three females 5 months old ADAM10 mice as well as FVB/N wild-type mice)

| **Table S1**  **Gene ID** | **Gene Symbol** | **Fold change** | **Log Ratio** | ***d-value*** |
| --- | --- | --- | --- | --- |
| 66074 | 0610041E09Rik | 1.273 | 0.348 | *2.274* |
| 68493 | 1110007M04Rik | 1.365 | 0.449 | *2.264* |
| 68832 | 1110057K04Rik | 1.327 | 0.408 | *2.362* |
| 72014 | 1500005I02Rik | 0.739 | -0.437 | *-2.071* |
| 78327 | 1500012D20Rik | 0.695 | -0.524 | *-2.032* |
| 78896 | 1500015O10Rik | 0.705 | -0.505 | *-1.937* |
| 74213 | 1700009P03Rik | 1.507 | 0.592 | *2.630* |
| 66602 | 1700020I14Rik | 1.595 | 0.674 | *2.377* |
| 67039 | 2600011C06Rik | 1.698 | 0.764 | *2.276* |
| 67048 | 2610030H06Rik | 1.427 | 0.513 | *2.440* |
| 66580 | 2610101J03Rik | 1.532 | 0.615 | *2.278* |
| 69082 | 2610312B22Rik | 1.502 | 0.587 | *2.605* |
| 381820 | 2700089E24Rik | 1.382 | 0.467 | *2.302* |
| 72758 | 2810449C10Rik | 1.432 | 0.518 | *2.324* |
| 67246 | 2810474O19Rik | 1.506 | 0.591 | *2.329* |
| 73100 | 2900092D14Rik | 1.386 | 0.471 | *2.239* |
| 67302 | 3110050K21Rik | 1.613 | 0.690 | *2.524* |
| 67684 | 3300001P08Rik | 1.577 | 0.657 | *2.308* |
| 218734 | 3830406C13Rik | 1.282 | 0.358 | *2.616* |
| 78244 | 4930461P20Rik | 1.515 | 0.599 | *2.442* |
| 75051 | 4930578N16Rik | 1.320 | 0.400 | *2.345* |
| 68144 | 5031426D15Rik | 0.757 | -0.402 | *-2.120* |
| 213582 | 5330427D05Rik | 1.493 | 0.578 | *2.311* |
| 70591 | 5730455P16Rik | 1.451 | 0.537 | *2.365* |
| 234797 | 6430548M08Rik | 1.507 | 0.592 | *2.475* |
| 320037 | 6430604M11Rik | 1.616 | 0.692 | *2.347* |
| 77551 | 8030493P09Rik | 1.319 | 0.399 | *2.256* |
| 74549 | 9130404D08Rik | 1.439 | 0.525 | *2.252* |
| 212448 | 9330159F19Rik | 1.278 | 0.354 | *2.343* |
| 80515 | A030009H04Rik | 1.460 | 0.546 | *2.619* |
| 320026 | A330076H08Rik | 1.444 | 0.530 | *2.620* |
| 216742 | A730024A03Rik | 1.362 | 0.446 | *2.322* |
| 231570 | A830010M20Rik | 1.523 | 0.607 | *2.801* |
| 58238 | A830059I20Rik | 0.639 | -0.647 | *-3.102* |
| 77938 | A930008G19Rik | 0.695 | -0.525 | *-2.212* |
| 319317 | A930034L06Rik | 1.571 | 0.652 | *2.390* |
| 320271 | A930041I02Rik | 1.622 | 0.698 | *2.354* |
| 320024 | Aadacl1 | 1.508 | 0.593 | *2.423* |
| 11421 | Ace | 0.777 | -0.364 | *-1.926* |
| 11487 | Adam10 | 1.543 | 0.626 | *2.802* |
| 27360 | Add3 | 1.519 | 0.603 | *2.841* |
| 68465 | Adipor2 | 1.365 | 0.449 | *2.294* |
| 77559 | Agl | 1.324 | 0.405 | *2.270* |
|  | AK031991 | 1.247 | 0.319 | *2.325* |
|  | AK034213 | 1.469 | 0.555 | *2.428* |
|  | AK039168 | 1.613 | 0.690 | *2.442* |
|  | AK046230 | 1.909 | 0.933 | *3.242* |
|  | AK048573 | 1.911 | 0.934 | *3.276* |
|  | AK051827 | 1.323 | 0.404 | *2.489* |
|  | AK077428 | 0.757 | -0.401 | *-2.051* |
|  | AK084292 | 1.270 | 0.345 | *2.248* |
|  | AK084875 | 1.330 | 0.411 | *2.301* |
| 11658 | Alcam | 1.471 | 0.557 | *2.298* |
| 67667 | Alkbh8 | 1.756 | 0.812 | *2.368* |
| 11736 | Ankfy1 | 1.474 | 0.560 | *2.491* |
| 77087 | Ankrd11 | 1.530 | 0.614 | *2.683* |
| 106585 | Ankrd12 | 1.518 | 0.602 | *2.303* |
| 56503 | Ankrd49 | 1.359 | 0.443 | *2.301* |
| 211673 | Arfgef1 | 1.503 | 0.588 | *2.644* |
| 11855 | Arhgap5 | 1.621 | 0.697 | *2.526* |
| 54208 | Arl6ip1 | 1.267 | 0.341 | *2.553* |
| 74100 | Arpp21 | 1.399 | 0.484 | *2.727* |
| 66403 | Asf1a | 1.390 | 0.475 | *2.607* |
| 192195 | Ash1l | 1.474 | 0.560 | *2.308* |
| 11920 | Atm | 1.508 | 0.593 | *2.603* |
| 98660 | Atp1a2 | 0.792 | -0.337 | *-1.972* |
| 11941 | Atp2b2 | 1.734 | 0.794 | *2.717* |
| 66043 | Atp5d | 0.820 | -0.287 | *-1.760* |
| 21871 | Atp6v0a2 | 1.365 | 0.449 | *2.626* |
| 11980 | Atp8a1 | 1.543 | 0.626 | *2.558* |
| 22589 | Atrx | 1.507 | 0.592 | *2.876* |
| 104570 | AW011752 | 1.395 | 0.480 | *2.303* |
| 235461 | B230380D07Rik | 1.544 | 0.627 | *2.291* |
| 118452 | Baalc | 1.410 | 0.496 | *2.463* |
| 12013 | Bach1 | 1.318 | 0.398 | *2.360* |
| 67384 | Bag4 | 1.305 | 0.384 | *2.261* |
| 233812 | BC030336 | 1.534 | 0.617 | *2.665* |
| 231128 | BC037112 | 1.290 | 0.367 | *2.250* |
| 230676 | BC059842 | 0.798 | -0.325 | *-2.052* |
| 72567 | Bclaf1 | 1.416 | 0.502 | *2.346* |
| 12121 | Bicd1 | 1.233 | 0.302 | *2.315* |
| 30948 | Bin1 | 1.451 | 0.537 | *2.318* |
| 66689 | Btbd5 | 1.453 | 0.539 | *2.454* |
| 78533 | C030002O17Rik | 1.320 | 0.400 | *2.492* |
| 215708 | C030011O14Rik | 1.568 | 0.649 | *3.215* |
| 77607 | C030036D22Rik | 1.437 | 0.523 | *2.292* |
| 223332 | C130037N17Rik | 0.687 | -0.542 | *-2.030* |
| 319340 | C130065N10Rik | 1.419 | 0.505 | *2.353* |
| 217378 | C330021A05Rik | 1.370 | 0.454 | *2.481* |
| 320358 | C430003N24Rik | 1.375 | 0.459 | *2.497* |
| 241514 | C630007C17Rik | 1.648 | 0.721 | *2.809* |
| 245555 | C77370 | 1.505 | 0.590 | *2.368* |
| 12298 | Cacnb4 | 1.800 | 0.848 | *2.913* |
| 12322 | Camk2a | 1.779 | 0.831 | *2.547* |
| 213819 | Casd1 | 1.211 | 0.276 | *2.251* |
| 67500 | Ccar1 | 1.597 | 0.675 | *2.473* |
| 52713 | Ccdc59 | 1.455 | 0.541 | *2.665* |
| 16149 | Cd74 | 0.724 | -0.466 | *-2.270* |
| 212285 | Centd1 | 1.569 | 0.650 | *2.530* |
| 12633 | Cflar | 1.393 | 0.478 | *2.497* |
| 212862 | Chpt1 | 1.453 | 0.539 | *2.602* |
| 12704 | Cit | 1.476 | 0.562 | *2.405* |
| 12741 | Cldn5 | 0.791 | -0.338 | *-1.794* |
| 12803 | Cntf | 1.496 | 0.581 | *2.355* |
| 12831 | Col5a1 | 0.770 | -0.378 | *-1.878* |
| 208922 | Cpeb3 | 1.570 | 0.651 | *2.493* |
| 432508 | Cpsf6 | 1.470 | 0.556 | *2.437* |
| 66586 | Crls1 | 1.337 | 0.419 | *2.850* |
| 229663 | Csde1 | 1.348 | 0.431 | *2.263* |
| 13018 | Ctcf | 1.664 | 0.735 | *2.783* |
| 75717 | Cul5 | 1.701 | 0.766 | *2.582* |
| 52463 | Cxxc6 | 1.340 | 0.422 | *2.288* |
| 70086 | Cysltr2 | 1.307 | 0.386 | *2.251* |
| 238988 | D14Ertd171e | 1.254 | 0.326 | *2.278* |
| 27877 | D1Ertd471e | 1.519 | 0.603 | *2.813* |
| 241062 | D230012E17Rik | 1.388 | 0.473 | *2.300* |
| 320609 | D330017J20Rik | 1.307 | 0.386 | *2.345* |
| 320847 | D330040H18Rik | 1.470 | 0.556 | *2.280* |
| 56790 | D3Ertd300e | 0.801 | -0.321 | *-1.734* |
| 28006 | D6Wsu116e | 1.442 | 0.528 | *2.600* |
| 72108 | Ddhd2 | 1.594 | 0.673 | *2.483* |
| 68817 | Ddi2 | 1.470 | 0.556 | *2.295* |
| 212880 | Ddx46 | 1.254 | 0.327 | *2.473* |
| 13209 | Ddx6 | 1.679 | 0.748 | *2.588* |
| 110524 | Dgkq | 1.340 | 0.422 | *2.310* |
| 74754 | Dhcr24 | 1.575 | 0.655 | *2.459* |
| 72162 | Dhx36 | 1.443 | 0.529 | *2.530* |
| 233335 | Dmn | 1.741 | 0.800 | *2.672* |
| 70604 | Dnajb14 | 1.861 | 0.896 | *2.742* |
| 22791 | Dnajc2 | 1.571 | 0.652 | *2.900* |
| 103967 | Dnm3 | 1.634 | 0.708 | *2.545* |
| 99480 | Dnttip2 | 1.305 | 0.384 | *2.297* |
| 67117 | Dynlt3 | 1.353 | 0.436 | *2.325* |
| 78000 | E130108L08Rik | 1.768 | 0.822 | *2.639* |
| 13618 | Ednrb | 1.364 | 0.448 | *2.506* |
| 13669 | Eif3s10 | 1.520 | 0.604 | *2.901* |
| 56347 | Eif3s8 | 1.699 | 0.765 | *2.429* |
| 223527 | Eny2 | 1.645 | 0.718 | *2.897* |
| 13822 | Epb4.1l2 | 1.689 | 0.756 | *2.481* |
| 13838 | Epha4 | 1.622 | 0.698 | *2.439* |
| 13855 | Epn2 | 1.460 | 0.546 | *2.443* |
| 67456 | Ergic2 | 1.367 | 0.451 | *2.316* |
| 14020 | Evi5 | 1.649 | 0.722 | *2.528* |
| 67731 | Fbxo32 | 1.365 | 0.449 | *2.265* |
| 246179 | Fcmd | 1.481 | 0.567 | *2.450* |
| 14205 | Figf | 1.237 | 0.307 | *2.238* |
| 54418 | Fmn2 | 1.340 | 0.422 | *2.316* |
| 14264 | Fmod | 0.743 | -0.428 | *-1.856* |
| 14348 | Fut9 | 1.804 | 0.851 | *3.244* |
| 69823 | Fyttd1 | 1.385 | 0.470 | *2.283* |
| 14397 | Gabra4 | 1.542 | 0.625 | *2.316* |
| 14417 | Gad2 | 1.636 | 0.710 | *3.290* |
| 271786 | Galnt13 | 1.334 | 0.416 | *2.349* |
| 14580 | Gfap | 0.783 | -0.353 | *-1.919* |
| 14660 | Gls | 1.472 | 0.558 | *2.418* |
| 229363 | Gmps | 1.708 | 0.772 | *2.255* |
| 14674 | Gna13 | 1.502 | 0.587 | *2.257* |
| 14677 | Gnai1 | 1.576 | 0.656 | *2.911* |
| 14680 | Gnal | 1.598 | 0.676 | *3.061* |
| 14696 | Gnb4 | 1.536 | 0.619 | *2.967* |
| 52389 | Gpr123 | 1.556 | 0.638 | *2.301* |
| 53623 | Gria3 | 1.480 | 0.566 | *2.388* |
| 56637 | Gsk3b | 1.540 | 0.623 | *2.382* |
| 14960 | H2-Aa | 0.728 | -0.457 | *-2.033* |
| 14961 | H2-Ab1 | 0.690 | -0.536 | *-2.311* |
| 29877 | Hdgfrp3 | 1.387 | 0.472 | *2.417* |
| 207304 | Hectd1 | 1.714 | 0.777 | *2.519* |
| 67768 | Hemk2 | 1.424 | 0.510 | *2.784* |
| 15208 | Hes5 | 0.749 | -0.416 | *-1.800* |
| 217082 | Hlf | 1.401 | 0.486 | *2.318* |
| 74326 | Hnrpr | 1.466 | 0.552 | *2.484* |
| 15516 | Hsp90ab1 | 1.627 | 0.702 | *2.511* |
| 22027 | Hsp90b1 | 1.775 | 0.828 | *3.006* |
| 18415 | Hspa4l | 1.424 | 0.510 | *2.678* |
| 15903 | Id3 | 0.809 | -0.305 | *-1.958* |
| 70110 | Ifi35 | 0.716 | -0.482 | *-1.950* |
| 66141 | Ifitm3 | 0.583 | -0.778 | *-1.889* |
| 16002 | Igf2 | 0.677 | -0.563 | *-2.138* |
| 16008 | Igfbp2 | 0.766 | -0.384 | *-2.002* |
| 16017 | Igh-4 | 0.747 | -0.421 | *-2.085* |
| 16186 | Il2rg | 0.745 | -0.424 | *-1.742* |
| 242291 | Impad1 | 1.676 | 0.745 | *2.308* |
| 101490 | Inpp5f | 1.467 | 0.553 | *2.335* |
| 72999 | Insig2 | 1.306 | 0.385 | *2.284* |
| 15944 | Irgm | 0.755 | -0.405 | *-1.836* |
| 16419 | Itgb5 | 0.786 | -0.347 | *-2.185* |
| 214899 | Jarid1a | 1.450 | 0.536 | *2.383* |
| 16502 | Kcnc1 | 1.590 | 0.669 | *2.482* |
| 16508 | Kcnd2 | 1.466 | 0.552 | *2.416* |
| 16510 | Kcnh1 | 1.398 | 0.483 | *2.291* |
| 16561 | Kif1b | 1.555 | 0.637 | *2.573* |
| 16568 | Kif3a | 1.653 | 0.725 | *2.546* |
| 16573 | Kif5b | 1.904 | 0.929 | *3.186* |
| 16574 | Kif5c | 1.656 | 0.728 | *2.463* |
| 16597 | Klf12 | 1.488 | 0.573 | *2.283* |
| 79264 | Krit1 | 1.492 | 0.577 | *2.399* |
| 16803 | Lbp | 0.739 | -0.436 | *-1.822* |
| 269629 | Lhfpl3 | 1.346 | 0.429 | *2.320* |
| 110829 | Lims1 | 1.533 | 0.616 | *2.309* |
| 108030 | Lin7a | 1.346 | 0.429 | *2.260* |
| 16889 | Lip1 | 1.494 | 0.579 | *2.675* |
| 16905 | Lmna | 0.722 | -0.470 | *-2.054* |
| 240028 | Lnpep | 1.444 | 0.530 | *2.549* |
| 434128 | LOC434128 | 1.544 | 0.627 | *2.333* |
| 226856 | Lpgat1 | 1.757 | 0.813 | *2.749* |
| 319387 | Lphn3 | 1.295 | 0.373 | *2.344* |
| 16971 | Lrp1 | 0.707 | -0.500 | *-2.139* |
| 16981 | Lrrn3 | 1.273 | 0.348 | *2.308* |
| 107065 | Lrrtm2 | 1.397 | 0.482 | *2.853* |
| 16998 | Ltbp3 | 0.791 | -0.338 | *-2.180* |
| 233271 | Luzp2 | 1.757 | 0.813 | *2.631* |
| 50791 | Magi2 | 1.426 | 0.512 | *2.399* |
| 17153 | Mal | 1.261 | 0.334 | *2.287* |
| 13589 | Mapre1 | 1.432 | 0.518 | *2.330* |
| 57438 | March7 | 1.406 | 0.492 | *2.485* |
| 232087 | Mat2a | 1.359 | 0.443 | *2.370* |
| 78771 | Mctp1 | 1.413 | 0.499 | *2.808* |
| 17289 | Mertk | 1.354 | 0.437 | *2.498* |
| 56217 | Mpp5 | 1.498 | 0.583 | *2.400* |
| 17765 | Mtf2 | 1.462 | 0.548 | *2.427* |
| 320713 | Mysm1 | 1.452 | 0.538 | *2.252* |
| 74838 | Narg1 | 1.439 | 0.525 | *2.402* |
| 66897 | Narg1l | 1.382 | 0.467 | *2.480* |
| 269198 | Nbeal1 | 1.499 | 0.584 | *2.662* |
| 71175 | Nipbl | 1.594 | 0.673 | *2.470* |
| 192167 | Nlgn1 | 1.518 | 0.602 | *2.386* |
| 70930 | Nol8 | 1.389 | 0.474 | *2.325* |
| 50490 | Nox4 | 1.692 | 0.759 | *3.540* |
| 353187 | Nr1d2 | 1.290 | 0.367 | *2.404* |
| 22026 | Nr2c2 | 1.473 | 0.559 | *2.283* |
| 23962 | Oasl2 | 0.646 | -0.630 | *-1.844* |
| 23965 | Odz3 | 1.432 | 0.518 | *2.262* |
| 18813 | Pa2g4 | 1.423 | 0.509 | *2.485* |
| 18514 | Pbx1 | 1.482 | 0.568 | *2.345* |
| 18526 | Pcdh10 | 1.398 | 0.483 | *2.568* |
| 18549 | Pcsk2 | 1.492 | 0.577 | *2.393* |
| 231887 | Pdap1 | 1.576 | 0.656 | *2.337* |
| 56426 | Pdcd10 | 1.434 | 0.520 | *2.761* |
| 18573 | Pde1a | 1.796 | 0.845 | *3.130* |
| 18596 | Pdgfrb | 0.800 | -0.322 | *-1.867* |
| 27273 | Pdk4 | 1.297 | 0.375 | *2.420* |
| 56376 | Pdlim5 | 1.376 | 0.461 | *2.303* |
| 18613 | Pecam1 | 0.778 | -0.363 | *-2.093* |
| 70974 | Pgm2l1 | 1.420 | 0.506 | *2.348* |
| 54383 | Phc2 | 0.794 | -0.333 | *-1.924* |
| 228829 | Phf20 | 1.365 | 0.449 | *2.481* |
| 224938 | Pja2 | 1.518 | 0.602 | *2.346* |
| 18795 | Plcb1 | 1.652 | 0.724 | *3.345* |
| 18798 | Plcb4 | 1.480 | 0.566 | *2.451* |
| 67448 | Plxdc2 | 1.390 | 0.475 | *2.750* |
| 228005 | Ppig | 1.636 | 0.710 | *2.577* |
| 243382 | Ppm1k | 1.620 | 0.696 | *2.576* |
| 242083 | Ppm1l | 1.604 | 0.682 | *2.277* |
| 26932 | Ppp2r5e | 1.578 | 0.658 | *2.298* |
| 108079 | Prkaa2 | 1.408 | 0.494 | *2.729* |
| 56194 | Prpf40a | 1.540 | 0.623 | *3.101* |
| 19215 | Ptgds | 0.766 | -0.384 | *-2.092* |
| 19243 | Ptp4a1 | 1.242 | 0.313 | *2.327* |
| 19281 | Ptprt | 1.409 | 0.495 | *2.354* |
| 19291 | Purb | 1.737 | 0.797 | *2.858* |
| 226412 | R3hdm1 | 1.382 | 0.467 | *2.430* |
| 19328 | Rab12 | 1.250 | 0.322 | *2.259* |
| 67790 | Rab39b | 1.728 | 0.789 | *2.532* |
| 19346 | Rab6 | 1.638 | 0.712 | *2.810* |
| 270192 | Rab6b | 1.696 | 0.762 | *2.837* |
| 56044 | Rala | 1.582 | 0.662 | *2.544* |
| 54409 | Ramp2 | 1.693 | 0.760 | *2.640* |
| 218397 | Rasa1 | 1.428 | 0.514 | *2.266* |
| 12421 | Rb1cc1 | 1.860 | 0.895 | *3.100* |
| 83486 | Rbm5 | 1.627 | 0.702 | *2.290* |
| 19682 | Rdh5 | 0.775 | -0.368 | *-1.844* |
| 19687 | Recc1 | 1.501 | 0.586 | *2.488* |
| 19737 | Rgs5 | 1.677 | 0.746 | *2.390* |
| 51869 | Rif1 | 1.401 | 0.486 | *2.364* |
| 170791 | Rnpc2 | 1.666 | 0.736 | *2.314* |
| 230257 | Rod1 | 1.281 | 0.357 | *2.546* |
| 19883 | Rora | 1.483 | 0.569 | *2.461* |
| 19894 | Rph3a | 1.470 | 0.556 | *2.352* |
| 20019 | Rpo1-4 | 1.378 | 0.463 | *2.428* |
| 382985 | Rrm2b | 1.675 | 0.744 | *2.798* |
| 233532 | Rsf1 | 1.610 | 0.687 | *2.474* |
| 67610 | Rspry1 | 1.383 | 0.468 | *2.334* |
| 68585 | Rtn4 | 1.542 | 0.625 | *2.344* |
| 67775 | Rtp4 | 0.591 | -0.759 | *-2.088* |
| 70432 | Rufy2 | 1.490 | 0.575 | *2.749* |
| 52822 | Rufy3 | 1.218 | 0.285 | *2.241* |
| 20201 | S100a8 | 0.587 | -0.768 | *-2.327* |
| 20202 | S100a9 | 0.668 | -0.582 | *-2.146* |
| 20265 | Scn1a | 1.518 | 0.602 | *2.617* |
| 66244 | Sdccag1 | 1.669 | 0.739 | *2.709* |
| 319322 | Sf3b2 | 1.695 | 0.761 | *2.580* |
| 20377 | Sfrp1 | 0.793 | -0.335 | *-1.730* |
| 73094 | Sgip1 | 0.755 | -0.405 | *-1.882* |
| 71781 | Slc16a14 | 1.396 | 0.481 | *2.269* |
| 214663 | Slc25a29 | 0.826 | -0.276 | *-1.821* |
| 212980 | Slc45a3 | 0.737 | -0.441 | *-1.774* |
| 239250 | Slitrk6 | 1.324 | 0.405 | *2.293* |
| 66660 | Sltm | 1.710 | 0.774 | *2.596* |
| 93761 | Smarca1 | 1.309 | 0.388 | *2.351* |
| 67155 | Smarca2 | 1.405 | 0.491 | *2.298* |
| 20587 | Smarcb1 | 0.803 | -0.317 | *-1.995* |
| 24061 | Smc1l1 | 1.481 | 0.567 | *2.563* |
| 226026 | Smc5l1 | 1.330 | 0.411 | *2.415* |
| 67241 | Smc6l1 | 1.608 | 0.685 | *2.898* |
| 20637 | Snrp70 | 1.536 | 0.619 | *2.286* |
| 66042 | Sostdc1 | 0.725 | -0.464 | *-1.768* |
| 20728 | Spic | 0.721 | -0.471 | *-1.851* |
| 20742 | Spnb2 | 1.729 | 0.790 | *2.855* |
| 114716 | Spred2 | 1.514 | 0.598 | *2.653* |
| 51796 | Srrm1 | 1.598 | 0.676 | *2.303* |
| 20823 | Ssb | 1.441 | 0.527 | *2.356* |
| 240690 | St18 | 1.283 | 0.360 | *2.267* |
| 110920 | Stch | 1.346 | 0.429 | *2.353* |
| 331401 | Thoc2 | 1.584 | 0.664 | *2.273* |
| 21838 | Thy1 | 1.366 | 0.450 | *3.014* |
| 99887 | Tmem56 | 1.490 | 0.575 | *2.248* |
| 21961 | Tns1 | 1.505 | 0.590 | *2.245* |
| 21969 | Top1 | 1.308 | 0.387 | *2.366* |
| 21976 | Top3b | 0.803 | -0.316 | *-1.804* |
| 30935 | Tor3a | 1.310 | 0.390 | *2.357* |
| 108989 | Tpr | 1.763 | 0.818 | *3.083* |
| 217069 | Trim25 | 0.760 | -0.395 | *-2.193* |
| 20822 | Trove2 | 1.250 | 0.322 | *2.308* |
| 22139 | Ttr | 0.763 | -0.390 | *-1.880* |
| 72736 | Txndc1 | 1.266 | 0.340 | *2.382* |
| 52837 | Txndc13 | 1.640 | 0.714 | *2.819* |
| 22215 | Ube3a | 1.469 | 0.555 | *2.293* |
| 170644 | Ubn1 | 1.611 | 0.688 | *2.491* |
| 67812 | Ubxd2 | 1.538 | 0.621 | *2.592* |
| 170707 | Usp48 | 1.519 | 0.603 | *2.350* |
| 70675 | Vcpip1 | 1.293 | 0.371 | *2.360* |
| 22344 | Vezf1 | 1.287 | 0.364 | *2.254* |
| 22370 | Vtn | 0.778 | -0.362 | *-1.925* |
| 22371 | Vwf | 0.715 | -0.484 | *-2.099* |
| 225131 | Wac | 1.372 | 0.456 | *2.244* |
| 73178 | Wasl | 1.799 | 0.847 | *3.252* |
| 57750 | Wdr12 | 1.391 | 0.476 | *2.446* |
| 232341 | Wnk1 | 1.844 | 0.883 | *2.947* |
| 24128 | Xrn2 | 1.410 | 0.496 | *2.385* |
| 67864 | Yipf4 | 1.327 | 0.408 | *2.245* |
| 22631 | Ywhaz | 1.455 | 0.541 | *2.257* |
| 16969 | Zbtb7a | 1.556 | 0.638 | *2.841* |
| 70579 | Zc3h11a | 1.476 | 0.562 | *2.294* |
| 70546 | Zdhhc2 | 1.313 | 0.393 | *2.264* |
| 18139 | Zfml | 1.201 | 0.264 | *2.310* |
| 59057 | Zfp191 | 1.301 | 0.380 | *2.280* |
| 22688 | Zfp26 | 1.282 | 0.358 | *2.336* |
| 244891 | Zfp291 | 1.488 | 0.573 | *2.687* |
| 77652 | Zfp422-rs1 | 1.538 | 0.621 | *2.537* |
| 235682 | Zfp445 | 1.607 | 0.684 | *2.665* |
| 238690 | Zfp458 | 1.467 | 0.553 | *2.254* |
| 52397 | Zfp644 | 1.530 | 0.614 | *2.786* |
| 72611 | Zfp655 | 1.256 | 0.329 | *2.238* |
| 22772 | Zic2 | 1.941 | 0.957 | *3.062* |
| *67263* | *Zswim6* | *1.380* | *0.465* | *2.472* |

Table S2 Complete list of significantly regulated genes in mono-transgenic dnADAM10 mice (three females 5 months old, dnADAM10 mice as well as FVB/N wild-type mice)

| **Table S2**  **Gene ID** | **Gene Symbol** | **Fold change** | **Log Ratio** | **d-value** |
| --- | --- | --- | --- | --- |
| 65971 | 1700021K02Rik | 0.762 | -0.393 | -1.854 |
| 75667 | 1700030E15Rik | 0.746 | -0.423 | -1.861 |
| 72493 | 2610202C22Rik | 0.789 | -0.342 | -1.814 |
| 66494 | 2610524G07Rik | 0.754 | -0.407 | -1.575 |
| 76795 | 2700008N14Rik | 0.824 | -0.280 | -1.574 |
| 217700 | 4632408A20Rik | 0.783 | -0.352 | -1.440 |
| 75015 | 4930503B20Rik | 0.750 | -0.415 | -2.001 |
| 75090 | 4930519D14Rik | 0.660 | -0.599 | -2.700 |
| 66625 | 5730406M06Rik | 1.350 | 0.433 | 1.607 |
| 74541 | 8430437L04Rik | 0.668 | -0.583 | -2.388 |
| 319810 | 9530001P21Rik | 0.718 | -0.477 | -2.363 |
| 219065 | A630038E17Rik | 0.772 | -0.373 | -1.669 |
| 11487 | Adam10 | 2.579 | 1.367 | 9.101 |
| 229709 | Ahcyl1 | 1.381 | 0.466 | 1.548 |
| 233208 | AI480556 | 0.764 | -0.389 | -1.728 |
|  | AK139137 | 1.459 | 0.545 | 1.805 |
| 218214 | Aof1 | 0.700 | -0.514 | -2.111 |
| 11771 | Ap2a1 | 0.783 | -0.352 | -1.430 |
| 74100 | Arpp21 | 1.313 | 0.393 | 1.655 |
| 232975 | Atp1a3 | 0.774 | -0.369 | -1.781 |
| 22589 | Atrx | 1.392 | 0.477 | 1.533 |
| 232023 | AW146242 | 0.764 | -0.388 | -1.820 |
| 12010 | B2m | 0.791 | -0.338 | -1.418 |
| 231803 | Bcdin3 | 0.806 | -0.311 | -1.719 |
| 72567 | Bclaf1 | 1.274 | 0.349 | 1.692 |
| 109880 | Braf | 0.568 | -0.815 | -5.060 |
| 77389 | C030032F19Rik | 1.406 | 0.492 | 1.571 |
| 241514 | C630007C17Rik | 1.455 | 0.541 | 1.550 |
| 54598 | Calcrl | 0.626 | -0.675 | -2.137 |
| 12322 | Camk2a | 0.686 | -0.544 | -2.523 |
| 235604 | Camkv | 0.830 | -0.269 | -1.434 |
| 12772 | Ccr2 | 0.724 | -0.465 | -1.783 |
| 16149 | Cd74 | 0.737 | -0.441 | -2.329 |
| 12615 | Cenpa | 0.760 | -0.395 | -2.017 |
| 53621 | Cnot4 | 0.751 | -0.414 | -2.218 |
| 12834 | Col6a2 | 0.807 | -0.309 | -1.540 |
| 226139 | Cox15 | 1.236 | 0.306 | 1.548 |
| 12944 | Crp | 0.694 | -0.526 | -2.146 |
| 228714 | Csrp2bp | 0.702 | -0.510 | -3.173 |
| 57349 | Cxcl7 | 0.663 | -0.593 | -1.843 |
| 13058 | Cybb | 0.790 | -0.340 | -1.452 |
| 13175 | Dcamkl1 | 1.501 | 0.586 | 1.610 |
| 72108 | Ddhd2 | 1.524 | 0.608 | 1.649 |
| 103551 | E130012A19Rik | 0.760 | -0.395 | -1.809 |
| 319670 | Eml5 | 1.288 | 0.365 | 1.654 |
| 12140 | Fabp7 | 1.691 | 0.758 | 2.107 |
| 66930 | Fank1 | 0.683 | -0.549 | -2.411 |
| 14130 | Fcgr2b | 0.792 | -0.336 | -1.434 |
| 14281 | Fos | 0.669 | -0.580 | -1.805 |
| 14417 | Gad2 | 1.422 | 0.508 | 1.683 |
| 235952 | Gm189 | 0.655 | -0.610 | -2.405 |
| 14674 | Gna13 | 1.353 | 0.436 | 1.639 |
| 14682 | Gnaq | 1.323 | 0.404 | 1.621 |
| 66964 | Golt1b | 1.271 | 0.346 | 1.488 |
| 17444 | Grap2 | 0.644 | -0.634 | -2.161 |
| 14960 | H2-Aa | 0.732 | -0.451 | -2.230 |
| 14961 | H2-Ab1 | 0.692 | -0.531 | -2.841 |
| 14964 | H2-D1 | 0.819 | -0.288 | -1.565 |
| 15000 | H2-DMb2 | 0.751 | -0.414 | -1.843 |
| 99296 | Hrh3 | 0.772 | -0.373 | -1.646 |
| 15525 | Hspa4 | 1.306 | 0.385 | 1.578 |
| 15557 | Htr1f | 1.363 | 0.447 | 1.539 |
| 15937 | Ier3 | 0.775 | -0.367 | -1.803 |
| 15953 | Ifi47 | 0.751 | -0.413 | -1.388 |
| 15957 | Ifit1 | 0.671 | -0.576 | -1.671 |
| 66141 | Ifitm3 | 0.629 | -0.670 | -1.605 |
| 16002 | Igf2 | 0.711 | -0.492 | -1.976 |
| 16017 | Igh-4 | 0.795 | -0.331 | -1.576 |
| 16069 | Igj | 0.737 | -0.440 | -1.636 |
| 78908 | Igsf3 | 1.311 | 0.391 | 1.777 |
| 16210 | Impact | 1.329 | 0.410 | 1.617 |
| 15944 | Irgm | 0.694 | -0.527 | -2.277 |
| 16391 | Isgf3g | 0.745 | -0.424 | -1.818 |
| 16533 | Kcnmb1 | 0.788 | -0.344 | -1.939 |
| 16560 | Kif1a | 0.798 | -0.326 | -1.633 |
| 16594 | Klc2 | 0.789 | -0.341 | -1.485 |
| 19039 | Lgals3bp | 0.774 | -0.369 | -1.362 |
| 16859 | Lgals9 | 0.763 | -0.390 | -1.662 |
| 668580 | LOC668580 | 0.714 | -0.485 | -2.134 |
| 226856 | Lpgat1 | 1.426 | 0.512 | 1.436 |
| 17105 | Lyzs | 0.757 | -0.401 | -2.036 |
| 17116 | Mab21l1 | 0.741 | -0.433 | -1.886 |
| 17153 | Mal | 1.286 | 0.363 | 2.023 |
| 26416 | Mapk14 | 1.331 | 0.413 | 1.665 |
| 207911 | Mchr1 | 0.718 | -0.477 | -3.057 |
| 71567 | Mcmdc1 | 0.721 | -0.471 | -1.925 |
| 17260 | Mef2c | 1.433 | 0.519 | 1.507 |
| 230082 | Nol6 | 0.793 | -0.335 | -1.828 |
| 78593 | Nrip3 | 1.425 | 0.511 | 1.661 |
| 23962 | Oasl2 | 0.660 | -0.600 | -1.770 |
| 244723 | Olfm2 | 0.773 | -0.372 | -1.953 |
| 66246 | Osgep | 0.830 | -0.268 | -1.582 |
| 243771 | Parp12 | 0.753 | -0.409 | -2.069 |
| 53357 | Pla2g6 | 0.736 | -0.442 | -2.154 |
| 231507 | Plac8 | 0.636 | -0.652 | -2.086 |
| 18823 | Plp1 | 1.358 | 0.442 | 1.565 |
| 18845 | Plxna2 | 1.378 | 0.463 | 1.431 |
| 66979 | Pole4 | 0.740 | -0.435 | -2.135 |
| 20020 | Polr2a | 0.624 | -0.680 | -3.889 |
| 18993 | Pou3f3 | 1.294 | 0.372 | 1.608 |
| 319468 | Ppm1h | 0.832 | -0.265 | -1.473 |
| 26931 | Ppp2r5c | 1.253 | 0.325 | 1.445 |
| 19113 | Prlpe | 0.758 | -0.400 | -1.512 |
| 72242 | Psg21 | 0.783 | -0.352 | -1.773 |
| 27388 | Ptdss2 | 0.744 | -0.427 | -2.787 |
| 19293 | Pvalb | 0.847 | -0.240 | -1.493 |
| 52118 | Pvr | 0.666 | -0.587 | -2.473 |
| 19317 | Qk | 1.312 | 0.392 | 1.522 |
| 19341 | Rab4a | 0.799 | -0.323 | -1.553 |
| 54409 | Ramp2 | 1.604 | 0.682 | 1.623 |
| 83486 | Rbm5 | 1.524 | 0.608 | 1.859 |
| 19736 | Rgs4 | 1.339 | 0.421 | 1.843 |
| 75745 | Rian | 1.374 | 0.458 | 1.435 |
| 56736 | Rnf14 | 1.446 | 0.532 | 1.526 |
| 78785 | Rsnl2 | 1.290 | 0.367 | 1.558 |
| 67775 | Rtp4 | 0.653 | -0.614 | -1.678 |
| 20201 | S100a8 | 0.592 | -0.757 | -2.780 |
| 20202 | S100a9 | 0.696 | -0.522 | -2.181 |
| 20265 | Scn1a | 1.477 | 0.563 | 1.543 |
| 170729 | Scrt1 | 0.782 | -0.354 | -1.384 |
| 71514 | Sfpq | 1.221 | 0.288 | 1.461 |
| 212980 | Slc45a3 | 0.572 | -0.807 | -3.469 |
| 229706 | Slc6a17 | 0.840 | -0.251 | -1.536 |
| 20538 | Slc6a2 | 0.650 | -0.621 | -2.316 |
| 20677 | Sox4 | 1.407 | 0.493 | 1.801 |
| 54141 | Spag5 | 0.797 | -0.327 | -1.693 |
| 73991 | Spg3a | 0.786 | -0.348 | -1.851 |
| 114715 | Spred1 | 0.710 | -0.495 | -2.968 |
| 70358 | Steap1 | 0.681 | -0.554 | -2.312 |
| 320165 | Tacc1 | 1.375 | 0.459 | 1.588 |
| 331401 | Thoc2 | 1.479 | 0.565 | 1.638 |
| 21838 | Thy1 | 2.034 | 1.024 | 6.888 |
| 21984 | Tpbpa | 0.590 | -0.761 | -3.160 |
| 22129 | Ttc3 | 1.475 | 0.561 | 1.618 |
| 21429 | Ubtf | 1.380 | 0.465 | 1.759 |
| 72094 | Ugt2a3 | 0.748 | -0.419 | -2.296 |
| 76654 | Upp2 | 0.776 | -0.366 | -1.864 |
| 24110 | Usp18 | 0.664 | -0.591 | -2.095 |
| 22290 | Uty | 0.781 | -0.356 | -1.552 |
| 22682 | Za20d2 | 1.357 | 0.440 | 1.587 |
| 56490 | Zbtb20 | 1.323 | 0.404 | 1.600 |
| 70579 | Zc3h11a | 1.368 | 0.452 | 2.082 |
| 24136 | Zfhx1b | 1.386 | 0.471 | 1.680 |

Table S3 Complete list of significantly regulated genes in double-transgenic ADAM10/APP_[V717I]_ mice (three female and three male 5 months old, ADAM10/APP_[V717I]_ mice as well as APP_[V717I]_ mice)

| **Table S3**  **Gene ID** | **Gene Symbol** | **Fold change** | **Log Ratio** | **d-value** |
| --- | --- | --- | --- | --- |
| 24050 | Sept3 | 0.754 | -0.407 | -2.079 |
| 103080 | Sept10 | 1.262 | 0.336 | 2.130 |
| 68327 | 0610007P22Rik | 1.241 | 0.312 | 2.060 |
| 66058 | 0610011I04Rik | 0.809 | -0.306 | -1.806 |
| 67672 | 0610040B10Rik | 1.312 | 0.392 | 2.284 |
| 223601 | 0910001A06Rik | 1.285 | 0.362 | 2.195 |
| 68466 | 1110003F02Rik | 0.789 | -0.341 | -1.937 |
| 68725 | 1110032F04Rik | 1.243 | 0.314 | 2.092 |
| 68795 | 1110059H15Rik | 1.339 | 0.421 | 2.388 |
| 268390 | 1110064P04Rik | 0.791 | -0.339 | -2.064 |
| 71706 | 1200006F02Rik | 0.836 | -0.259 | -1.652 |
| 68971 | 1500001M20Rik | 1.278 | 0.354 | 2.151 |
| 68965 | 1500010G04Rik | 0.774 | -0.370 | -1.873 |
| 66236 | 1500011B03Rik | 1.257 | 0.330 | 2.158 |
| 66931 | 1700010I14Rik | 1.274 | 0.349 | 2.128 |
| 73327 | 1700040I03Rik | 1.281 | 0.357 | 2.293 |
| 380773 | 1810035L17Rik | 1.258 | 0.331 | 2.064 |
| 546336 | 2010007L08Rik | 0.786 | -0.347 | -1.954 |
| 67098 | 2210403K04Rik | 1.337 | 0.419 | 2.498 |
| 71881 | 2310001A20Rik | 1.279 | 0.355 | 2.064 |
| 67859 | 2310002J15Rik | 0.827 | -0.274 | -1.772 |
| 70088 | 2310005N01Rik | 0.826 | -0.276 | -1.728 |
| 72315 | 2310015A05Rik | 1.255 | 0.328 | 2.074 |
| 75683 | 2310035P21Rik | 1.370 | 0.454 | 2.364 |
| 71918 | 2310047A01Rik | 0.816 | -0.293 | -1.821 |
| 66965 | 2310061F22Rik | 1.283 | 0.360 | 2.095 |
| 76585 | 2310069N01Rik | 0.799 | -0.324 | -1.997 |
| 76788 | 2410127E18Rik | 0.811 | -0.302 | -1.964 |
| 66299 | 2610019N06Rik | 1.281 | 0.357 | 2.176 |
| 72139 | 2610044O15Rik | 1.289 | 0.366 | 2.134 |
| 70434 | 2610201A13Rik | 1.275 | 0.350 | 2.196 |
| 72514 | 2610306H15Rik | 1.277 | 0.353 | 2.067 |
| 72505 | 2610319H10Rik | 0.803 | -0.316 | -1.821 |
| 72677 | 2810049E08Rik | 1.315 | 0.395 | 2.175 |
| 67059 | 2810409H07Rik | 1.283 | 0.360 | 2.074 |
| 76917 | 2810417J12Rik | 1.261 | 0.335 | 2.112 |
| 72828 | 2810457I06Rik | 0.796 | -0.330 | -2.223 |
| 67246 | 2810474O19Rik | 1.320 | 0.400 | 2.081 |
| 72931 | 2900010J23Rik | 1.344 | 0.426 | 2.094 |
| 72852 | 2900024O10Rik | 1.347 | 0.430 | 2.309 |
| 73005 | 2900072G11Rik | 1.329 | 0.410 | 2.199 |
| 66540 | 3110001A13Rik | 0.775 | -0.368 | -1.856 |
| 67289 | 3110021A11Rik | 0.790 | -0.340 | -1.985 |
| 68038 | 3110023E09Rik | 1.319 | 0.399 | 2.568 |
| 73168 | 3110027N22Rik | 1.317 | 0.397 | 2.386 |
| 73235 | 3110082D06Rik | 0.821 | -0.285 | -1.740 |
| 235542 | 3222402P14Rik | 0.796 | -0.330 | -2.013 |
| 67684 | 3300001P08Rik | 1.417 | 0.503 | 2.441 |
| 74026 | 4121402D02Rik | 0.776 | -0.365 | -2.260 |
| 320612 | 4832420A03Rik | 0.788 | -0.343 | -2.097 |
| 70925 | 4921511I16Rik | 1.281 | 0.357 | 2.204 |
| 232664 | 4921511K06Rik | 1.260 | 0.333 | 2.106 |
| 70887 | 4921520P21Rik | 1.262 | 0.336 | 2.062 |
| 68187 | 4921533L14Rik | 1.271 | 0.346 | 2.086 |
| 78108 | 4930414L22Rik | 1.267 | 0.341 | 2.068 |
| 74706 | 4930507D05Rik | 0.812 | -0.300 | -1.849 |
| 67735 | 4930528A17Rik | 1.291 | 0.369 | 2.173 |
| 75202 | 4930546H06Rik | 1.335 | 0.417 | 2.356 |
| 75924 | 4930566N20Rik | 0.816 | -0.293 | -1.814 |
| 329782 | 4930570G19Rik | 1.340 | 0.422 | 2.468 |
| 67753 | 4930579C15Rik | 1.268 | 0.343 | 2.135 |
| 71086 | 4933412E12Rik | 1.253 | 0.325 | 2.094 |
| 66771 | 4933439F18Rik | 0.799 | -0.324 | -2.014 |
| 68144 | 5031426D15Rik | 1.310 | 0.390 | 2.126 |
| 76679 | 5330417H12Rik | 0.824 | -0.279 | -1.665 |
| 237943 | 5430405G24Rik | 1.257 | 0.330 | 2.092 |
| 71431 | 5530401A10Rik | 0.796 | -0.329 | -2.022 |
| 67974 | 5730405I09Rik | 1.298 | 0.376 | 2.291 |
| 66641 | 5730470L24Rik | 1.264 | 0.338 | 2.067 |
| 76107 | 5830467E07Rik | 1.291 | 0.368 | 2.142 |
| 67786 | 6230424C14Rik | 1.267 | 0.341 | 2.150 |
| 331475 | 6430511F03 | 0.776 | -0.366 | -2.134 |
| 76886 | 6430514L14Rik | 0.763 | -0.390 | -1.821 |
| 212390 | 6430524H05Rik | 0.806 | -0.311 | -1.848 |
| 103012 | 6720401G13Rik | 1.334 | 0.416 | 2.198 |
| 77730 | 6720464I07Rik | 0.768 | -0.381 | -1.994 |
| 208292 | 9030612M13Rik | 0.757 | -0.402 | -2.149 |
| 231440 | 9130213B05Rik | 0.807 | -0.310 | -1.886 |
| 320227 | 9330121K16Rik | 1.263 | 0.337 | 2.142 |
| 226844 | 9630055N22Rik | 1.295 | 0.373 | 2.072 |
| 328563 | A330102K04Rik | 1.285 | 0.362 | 2.098 |
| 77773 | A330103N21Rik | 0.779 | -0.361 | -2.111 |
| 328309 | A530089L17 | 1.283 | 0.359 | 2.223 |
| 320317 | A830010M09Rik | 1.291 | 0.368 | 2.104 |
| 58238 | A830059I20Rik | 0.734 | -0.446 | -2.836 |
| 414093 | A830082N09Rik | 1.306 | 0.385 | 2.326 |
| 213006 | A930031D07Rik | 1.344 | 0.426 | 2.489 |
| 110446 | Acat1 | 1.274 | 0.349 | 2.196 |
| 56215 | Acin1 | 0.795 | -0.331 | -1.955 |
| 329910 | Acot11 | 0.812 | -0.301 | -1.620 |
| 171210 | Acot2 | 1.256 | 0.329 | 2.122 |
| 50790 | Acsl4 | 1.282 | 0.358 | 2.061 |
| 433256 | Acsl5 | 1.292 | 0.370 | 2.412 |
| 11487 | Adam10 | 1.483 | 0.569 | 3.048 |
| 432530 | Adcy1 | 0.810 | -0.304 | -1.880 |
| 68465 | Adipor2 | 0.781 | -0.357 | -1.868 |
| 11540 | Adora2a | 1.294 | 0.372 | 2.210 |
| 69597 | Afg3l2 | 1.257 | 0.330 | 2.131 |
| 106589 | AI449023 | 0.788 | -0.343 | -1.677 |
| 330941 | AI593442 | 1.278 | 0.354 | 2.111 |
| 101694 | AI854517 | 1.283 | 0.360 | 2.153 |
| 101197 | AI894139 | 1.288 | 0.365 | 2.169 |
| 56248 | Ak3 | 0.823 | -0.281 | -1.729 |
| 105387 | Akr1c14 | 1.262 | 0.336 | 2.080 |
| 11656 | Alas2 | 0.744 | -0.427 | -1.966 |
| 56454 | Aldh18a1 | 1.318 | 0.398 | 2.321 |
| 107747 | Aldh1l1 | 0.792 | -0.336 | -2.087 |
| 93835 | Amn | 1.353 | 0.436 | 2.527 |
| 27494 | Amot | 0.790 | -0.340 | -1.965 |
| 11735 | Ank3 | 1.344 | 0.427 | 2.159 |
| 106585 | Ankrd12 | 1.328 | 0.409 | 2.090 |
| 12306 | Anxa2 | 0.792 | -0.337 | -1.924 |
| 11787 | Apbb2 | 0.788 | -0.344 | -2.018 |
| 226548 | Aph1a | 0.807 | -0.309 | -1.879 |
| 11829 | Aqp4 | 0.735 | -0.445 | -2.240 |
| 228998 | Arfgap1 | 1.258 | 0.331 | 2.108 |
| 76117 | Arhgap15 | 1.275 | 0.351 | 2.139 |
| 71371 | Arid5b | 1.272 | 0.347 | 2.090 |
| 74840 | Armet | 0.748 | -0.418 | -1.945 |
| 109979 | Art3 | 1.255 | 0.328 | 2.131 |
| 76294 | Asb5 | 1.254 | 0.327 | 2.079 |
| 11484 | Aspa | 1.270 | 0.345 | 2.093 |
| 108888 | Atad3a | 1.257 | 0.330 | 2.109 |
| 74244 | Atg7 | 1.284 | 0.361 | 2.088 |
| 98660 | Atp1a2 | 0.820 | -0.287 | -1.746 |
| 11932 | Atp1b2 | 0.793 | -0.334 | -1.693 |
| 242341 | Atp6v0d2 | 0.809 | -0.305 | -1.962 |
| 20239 | Atxn2 | 0.821 | -0.285 | -1.834 |
| 99169 | AU015228 | 1.260 | 0.333 | 2.074 |
| 319974 | Auts2 | 0.803 | -0.316 | -1.981 |
| 78603 | B230216N24Rik | 1.280 | 0.356 | 2.161 |
| 235461 | B230380D07Rik | 0.759 | -0.398 | -1.996 |
| 26877 | B3galt1 | 1.291 | 0.368 | 2.125 |
| 329739 | B430201A12Rik | 0.761 | -0.394 | -2.000 |
| 320085 | B830012L14Rik | 1.292 | 0.370 | 2.083 |
| 319604 | B930006L02Rik | 0.813 | -0.299 | -1.892 |
| 320622 | B930068K11Rik | 0.816 | -0.294 | -1.733 |
| 208768 | BC031781 | 0.807 | -0.310 | -2.059 |
| 213056 | BC049806 | 0.766 | -0.384 | -2.251 |
| 268709 | BC055107 | 0.763 | -0.391 | -2.174 |
| 12043 | Bcl2 | 0.815 | -0.295 | -1.741 |
| 72567 | Bclaf1 | 1.326 | 0.407 | 2.183 |
| 503859 | BF642829 | 0.771 | -0.375 | -1.777 |
| 76895 | Bicd2 | 0.810 | -0.304 | -1.681 |
| 12122 | Bid | 1.291 | 0.369 | 2.278 |
| 109880 | Braf | 0.812 | -0.301 | -1.837 |
| 12227 | Btg2 | 1.342 | 0.424 | 2.357 |
| 77389 | C030032F19Rik | 1.316 | 0.396 | 2.182 |
| 319340 | C130065N10Rik | 0.751 | -0.414 | -2.099 |
| 12260 | C1qb | 0.778 | -0.363 | -1.785 |
| 12262 | C1qc | 0.780 | -0.359 | -1.777 |
| 67389 | C1qdc2 | 1.272 | 0.347 | 2.176 |
| 328322 | C230069N13 | 1.364 | 0.448 | 2.182 |
| 77625 | C530007A02Rik | 1.268 | 0.343 | 2.077 |
| 29867 | Cabp1 | 1.277 | 0.353 | 2.125 |
| 12300 | Cacng2 | 0.803 | -0.316 | -1.872 |
| 12317 | Calr | 0.779 | -0.360 | -1.930 |
| 12325 | Camk2g | 0.810 | -0.304 | -1.711 |
| 12319 | Car8 | 0.785 | -0.349 | -1.679 |
| 216285 | Cart1 | 1.265 | 0.339 | 2.082 |
| 12361 | Cask | 0.832 | -0.265 | -1.689 |
| 12398 | Cbfa2t3h | 0.838 | -0.255 | -1.607 |
| 12411 | Cbs | 0.837 | -0.256 | -1.616 |
| 12509 | Cd59a | 0.804 | -0.314 | -1.956 |
| 226751 | Cdc42bpa | 1.288 | 0.365 | 2.130 |
| 12575 | Cdkn1a | 0.779 | -0.360 | -2.004 |
| 214552 | Cep164 | 1.242 | 0.313 | 2.120 |
| 12626 | Cetn3 | 1.315 | 0.395 | 2.197 |
| 68567 | Cgref1 | 1.240 | 0.310 | 2.075 |
| 66917 | Chordc1 | 0.736 | -0.443 | -2.116 |
| 98388 | Chst10 | 0.839 | -0.253 | -1.578 |
| 12696 | Cirbp | 1.776 | 0.829 | 3.725 |
| 54124 | Cks1b | 1.254 | 0.327 | 2.119 |
| 12725 | Clcn3 | 0.798 | -0.325 | -1.761 |
| 104625 | Cnot6 | 0.773 | -0.371 | -2.037 |
| 231464 | Cnot6l | 0.796 | -0.329 | -1.898 |
| 66797 | Cntnap2 | 1.305 | 0.384 | 2.073 |
| 233824 | Cog7 | 1.277 | 0.353 | 2.160 |
| 12830 | Col4a5 | 0.807 | -0.310 | -2.025 |
| 54158 | Copg2as2 | 1.263 | 0.337 | 2.114 |
| 12870 | Cp | 0.807 | -0.310 | -1.866 |
| 235415 | Cplx3 | 1.276 | 0.352 | 2.294 |
| 76737 | Creld2 | 0.796 | -0.330 | -1.735 |
| 70625 | Crsp7 | 0.842 | -0.248 | -1.567 |
| 12955 | Cryab | 0.744 | -0.427 | -2.161 |
| 68631 | Cryl1 | 1.261 | 0.335 | 2.117 |
| 66609 | Cryzl1 | 0.801 | -0.320 | -2.081 |
| 12977 | Csf1 | 0.822 | -0.282 | -1.717 |
| 27373 | Csnk1e | 0.791 | -0.338 | -2.160 |
| 13000 | Csnk2a2 | 0.801 | -0.321 | -1.909 |
| 13024 | Ctla2a | 0.733 | -0.448 | -2.114 |
| 13046 | Cugbp1 | 0.821 | -0.285 | -1.814 |
| 244672 | Cwf19l2 | 1.287 | 0.364 | 2.064 |
| 67393 | Cxxc5 | 0.790 | -0.340 | -1.986 |
| 54151 | Cyhr1 | 0.818 | -0.289 | -1.794 |
| 16007 | Cyr61 | 1.298 | 0.376 | 2.213 |
| 28193 | D10Ucla1 | 0.739 | -0.436 | -2.133 |
| 52882 | D13Bwg1146e | 0.757 | -0.402 | -2.108 |
| 224019 | D16Bwg1494e | 1.251 | 0.323 | 2.083 |
| 67102 | D16Ertd472e | 0.828 | -0.272 | -1.607 |
| 52120 | D8Ertd354e | 1.247 | 0.318 | 2.107 |
| 13170 | Dbp | 1.486 | 0.571 | 2.843 |
| 67665 | Dctn4 | 1.281 | 0.357 | 2.117 |
| 69219 | Ddah1 | 0.757 | -0.401 | -2.233 |
| 54722 | Dfna5h | 0.803 | -0.316 | -1.714 |
| 209692 | Dhtkd1 | 1.321 | 0.402 | 2.266 |
| 13371 | Dio2 | 0.796 | -0.330 | -1.592 |
| 330938 | Dixdc1 | 0.825 | -0.278 | -1.770 |
| 50768 | Dlc1 | 0.794 | -0.332 | -1.894 |
| 56323 | Dnajb5 | 0.804 | -0.315 | -1.719 |
| 13430 | Dnm2 | 0.815 | -0.295 | -2.004 |
| 13434 | Dnmt2 | 1.279 | 0.355 | 2.158 |
| 208266 | Dot1l | 0.798 | -0.325 | -1.992 |
| 26757 | Dpysl4 | 1.264 | 0.338 | 2.142 |
| 13527 | Dtna | 1.271 | 0.346 | 2.240 |
| 21915 | Dtymk | 1.309 | 0.389 | 2.060 |
| 19252 | Dusp1 | 1.309 | 0.388 | 2.093 |
| 18218 | Dusp8 | 1.305 | 0.384 | 2.179 |
| 211948 | E430028B21Rik | 1.242 | 0.313 | 2.061 |
| 414123 | E530001K10Rik | 0.806 | -0.312 | -1.760 |
| 230857 | Ece1 | 0.801 | -0.320 | -1.853 |
| 77683 | Ehmt1 | 0.827 | -0.274 | -1.733 |
| 13664 | Eif1a | 1.283 | 0.359 | 2.452 |
| 66892 | Eif4e3 | 1.269 | 0.344 | 2.083 |
| 170757 | Eltd1 | 0.812 | -0.301 | -1.849 |
| 56205 | Ensa | 1.278 | 0.354 | 2.120 |
| 12499 | Entpd5 | 0.833 | -0.264 | -1.703 |
| 13841 | Epha7 | 1.300 | 0.379 | 2.146 |
| 20336 | Exoc4 | 0.829 | -0.271 | -1.730 |
| 56219 | Extl1 | 1.290 | 0.367 | 2.171 |
| 329628 | Fat4 | 1.255 | 0.328 | 2.065 |
| 269514 | Fbxl4 | 1.245 | 0.316 | 2.117 |
| 57443 | Fbxo3 | 0.812 | -0.300 | -1.801 |
| 14228 | Fkbp4 | 0.814 | -0.297 | -1.758 |
| 14254 | Flt1 | 0.787 | -0.345 | -2.064 |
| 55990 | Fmo2 | 0.795 | -0.331 | -1.758 |
| 72007 | Fndc3b | 0.760 | -0.395 | -2.497 |
| 14284 | Fosl2 | 0.787 | -0.345 | -1.875 |
| 14339 | Fts | 1.291 | 0.368 | 2.092 |
| 233908 | Fus | 1.375 | 0.459 | 2.419 |
| 14105 | Fusip1 | 0.809 | -0.305 | -1.900 |
| 56188 | Fxyd1 | 0.812 | -0.300 | -1.791 |
| 108150 | Galnt7 | 0.781 | -0.357 | -2.115 |
| 14455 | Gas5 | 1.283 | 0.360 | 2.069 |
| 14467 | Gbas | 1.270 | 0.345 | 2.133 |
| 14544 | Gda | 1.300 | 0.379 | 2.229 |
| 14580 | Gfap | 0.698 | -0.519 | -2.154 |
| 118446 | Gje1 | 1.349 | 0.432 | 2.453 |
| 170772 | Glcci1 | 1.304 | 0.383 | 2.325 |
| 69367 | Glrx2 | 1.252 | 0.324 | 2.066 |
| 229599 | Gm129 | 1.391 | 0.476 | 2.682 |
| 268491 | Gm1564 | 1.285 | 0.362 | 2.202 |
| 195209 | Gm22 | 0.807 | -0.310 | -1.890 |
| 210573 | Gm323 | 0.780 | -0.359 | -1.839 |
| 225743 | Gm96 | 0.810 | -0.304 | -2.001 |
| 14682 | Gnaq | 0.813 | -0.298 | -1.776 |
| 230737 | Gnl2 | 1.298 | 0.376 | 2.280 |
| 14571 | Gpd2 | 0.778 | -0.363 | -1.961 |
| 53872 | Gpiap1 | 0.813 | -0.299 | -1.832 |
| 14758 | Gpm6b | 0.769 | -0.379 | -2.219 |
| 224792 | Gpr116 | 1.279 | 0.355 | 2.154 |
| 67298 | Gprasp1 | 1.366 | 0.450 | 2.466 |
| 107022 | Gramd3 | 0.809 | -0.306 | -1.870 |
| 14799 | Gria1 | 0.776 | -0.365 | -1.975 |
| 14800 | Gria2 | 0.740 | -0.435 | -1.907 |
| 14804 | Grid2 | 0.806 | -0.312 | -1.793 |
| 382034 | Gse1 | 0.811 | -0.303 | -1.666 |
| 227835 | Gtdc1 | 1.278 | 0.354 | 2.112 |
| 29870 | Gtse1 | 0.821 | -0.285 | -1.733 |
| 14943 | Gzmf | 0.801 | -0.321 | -1.928 |
| 67552 | H2afy3 | 0.786 | -0.347 | -2.077 |
| 15000 | H2-DMb2 | 0.816 | -0.294 | -1.899 |
| 66044 | Hars2 | 1.275 | 0.351 | 2.093 |
| 15184 | Hdac5 | 0.822 | -0.282 | -1.820 |
| 78455 | Helz | 0.752 | -0.412 | -2.346 |
| 15258 | Hipk2 | 0.761 | -0.394 | -2.283 |
| 227399 | Hisppd1 | 1.334 | 0.416 | 2.290 |
| 15077 | Hist2h3c1 | 0.749 | -0.417 | -2.385 |
| 66867 | Hmg20a | 0.835 | -0.261 | -1.829 |
| 50926 | Hnrpdl | 1.312 | 0.392 | 2.169 |
| 15388 | Hnrpl | 1.308 | 0.387 | 2.305 |
| 74318 | Hod | 0.782 | -0.355 | -1.826 |
| 26557 | Homer2 | 0.774 | -0.370 | -2.144 |
| 15505 | Hsp110 | 0.753 | -0.409 | -1.940 |
| 14828 | Hspa5 | 0.716 | -0.481 | -1.984 |
| 56213 | Htra1 | 0.795 | -0.331 | -1.834 |
| 218215 | Ibrdc2 | 1.260 | 0.333 | 2.173 |
| 15925 | Ide | 0.778 | -0.362 | -2.163 |
| 15951 | Ifi204 | 1.268 | 0.343 | 2.104 |
| 76500 | Ihpk2 | 1.267 | 0.341 | 2.103 |
| 16170 | Il16 | 0.806 | -0.312 | -1.652 |
| 226180 | Ina | 0.782 | -0.354 | -2.048 |
| 16323 | Inhba | 0.718 | -0.477 | -2.551 |
| 231070 | Insig1 | 1.353 | 0.436 | 2.442 |
| 241226 | Itga8 | 1.270 | 0.345 | 2.112 |
| 16419 | Itgb5 | 0.832 | -0.265 | -1.649 |
| 16443 | Itsn1 | 0.826 | -0.275 | -1.839 |
| 117198 | Ivns1abp | 1.271 | 0.346 | 2.240 |
| 76071 | Jakmip1 | 1.256 | 0.329 | 2.082 |
| 76217 | Jakmip2 | 1.318 | 0.398 | 2.191 |
| 216850 | Jmjd3 | 0.740 | -0.435 | -2.319 |
| 16477 | Junb | 1.350 | 0.433 | 2.404 |
| 545156 | Kalrn | 1.323 | 0.404 | 2.532 |
| 72330 | Kbtbd5 | 1.286 | 0.363 | 2.143 |
| 16485 | Kcna1 | 0.773 | -0.371 | -1.836 |
| 268345 | Kcnc2 | 1.281 | 0.357 | 2.237 |
| 16531 | Kcnma1 | 1.278 | 0.354 | 2.138 |
| 231855 | KIAA0415 | 0.820 | -0.286 | -1.871 |
| 16551 | Kif11 | 1.267 | 0.341 | 2.118 |
| 110033 | Kif22 | 1.369 | 0.453 | 2.380 |
| 16573 | Kif5b | 0.792 | -0.336 | -1.687 |
| 16581 | Kifc2 | 1.277 | 0.353 | 2.067 |
| 50794 | Klf13 | 0.798 | -0.326 | -1.776 |
| 16635 | Klra4 | 0.782 | -0.355 | -1.953 |
| 16646 | Kpna1 | 0.837 | -0.256 | -1.660 |
| 268482 | Krt1-12 | 1.294 | 0.372 | 2.192 |
| 71369 | Krtap16-10 | 1.291 | 0.369 | 2.102 |
| 67260 | Lass4 | 1.266 | 0.340 | 2.073 |
| 246316 | Lgi2 | 1.320 | 0.400 | 2.210 |
| 22343 | Lin7c | 0.831 | -0.267 | -1.652 |
| 622976 | LOC622976 | 1.285 | 0.362 | 2.126 |
| 637127 | LOC637127 | 1.260 | 0.333 | 2.181 |
| 330814 | Lphn1 | 0.803 | -0.316 | -1.934 |
| 210126 | Lpp | 0.771 | -0.375 | -2.072 |
| 272589 | Lrrc35 | 0.801 | -0.320 | -1.832 |
| 54562 | Lrrc6 | 1.274 | 0.349 | 2.153 |
| 16998 | Ltbp3 | 1.291 | 0.368 | 2.202 |
| 110454 | Ly6a | 0.821 | -0.285 | -1.697 |
| 23934 | Ly6h | 1.287 | 0.364 | 2.102 |
| 105853 | Mal2 | 1.344 | 0.426 | 2.231 |
| 67729 | Mansc1 | 1.289 | 0.366 | 2.201 |
| 23938 | Map2k5 | 1.262 | 0.336 | 2.135 |
| 17762 | Mapt | 0.727 | -0.459 | -2.312 |
| 17118 | Marcks | 0.799 | -0.324 | -1.815 |
| 13728 | Mark2 | 0.817 | -0.291 | -1.922 |
| 108645 | Mat2b | 1.261 | 0.335 | 2.101 |
| 17193 | Mbd4 | 0.818 | -0.289 | -1.753 |
| 105559 | Mbnl2 | 0.796 | -0.330 | -1.904 |
| 71567 | Mcmdc1 | 1.254 | 0.326 | 2.161 |
| 52065 | Mfhas1 | 0.759 | -0.398 | -2.297 |
| 76574 | Mfsd2 | 0.760 | -0.395 | -2.105 |
| 59030 | Mkks | 0.749 | -0.416 | -2.427 |
| 17346 | Mknk1 | 1.273 | 0.348 | 2.113 |
| 214162 | Mll1 | 0.805 | -0.313 | -1.976 |
| 17354 | Mllt10 | 1.284 | 0.361 | 2.149 |
| 319817 | Mnab | 1.291 | 0.368 | 2.197 |
| 232157 | Mobk1b | 0.801 | -0.321 | -1.916 |
| 77853 | Msl2l1 | 1.270 | 0.345 | 2.212 |
| 17748 | Mt1 | 0.807 | -0.310 | -1.974 |
| 17750 | Mt2 | 0.780 | -0.359 | -2.141 |
| 17756 | Mtap2 | 0.786 | -0.347 | -1.935 |
| 67154 | Mtdh | 1.273 | 0.348 | 2.117 |
| 17764 | Mtf1 | 0.815 | -0.296 | -1.984 |
| 270685 | Mthfd1l | 1.332 | 0.414 | 2.305 |
| 17883 | Myh3 | 1.299 | 0.377 | 2.198 |
| 17909 | Myo10 | 0.824 | -0.280 | -1.751 |
| 17918 | Myo5a | 1.309 | 0.389 | 2.105 |
| 17926 | Myoc | 1.333 | 0.415 | 2.365 |
| 17933 | Myt1l | 1.308 | 0.387 | 2.127 |
| 20185 | Ncor1 | 0.799 | -0.323 | -1.967 |
| 17984 | Ndn | 1.315 | 0.395 | 2.209 |
| 74103 | Nebl | 0.807 | -0.309 | -1.889 |
| 338352 | Nell1 | 1.355 | 0.438 | 2.324 |
| 29861 | Neud4 | 0.802 | -0.319 | -2.042 |
| 18027 | Nfia | 0.799 | -0.323 | -1.864 |
| 18029 | Nfic | 0.807 | -0.310 | -1.842 |
| 18045 | Nfyb | 1.263 | 0.337 | 2.235 |
| 75570 | Nhej1 | 0.788 | -0.343 | -2.029 |
| 18082 | Nipsnap1 | 1.237 | 0.307 | 2.063 |
| 72293 | Nkd2 | 0.809 | -0.305 | -2.060 |
| 192167 | Nlgn1 | 1.381 | 0.466 | 2.470 |
| 18125 | Nos1 | 0.795 | -0.331 | -1.953 |
| 50490 | Nox4 | 1.319 | 0.399 | 2.224 |
| 225872 | Npas4 | 1.355 | 0.438 | 2.375 |
| 217166 | Nr1d1 | 1.365 | 0.449 | 2.636 |
| 18212 | Ntrk2 | 0.797 | -0.327 | -1.978 |
| 75686 | Nudt16 | 1.300 | 0.379 | 2.171 |
| 23964 | Odz2 | 1.274 | 0.349 | 2.083 |
| 23965 | Odz3 | 1.257 | 0.330 | 2.089 |
| 56177 | Olfm1 | 1.257 | 0.330 | 2.087 |
| 18378 | Omp | 0.790 | -0.340 | -1.907 |
| 18389 | Oprl1 | 1.330 | 0.411 | 2.066 |
| 71648 | Optn | 1.305 | 0.384 | 2.285 |
| 66844 | Ormdl2 | 0.817 | -0.292 | -1.657 |
| 72085 | Osgepl1 | 1.257 | 0.330 | 2.104 |
| 18451 | P4ha1 | 0.758 | -0.399 | -2.166 |
| 18481 | Pak3 | 0.773 | -0.371 | -1.947 |
| 74229 | Paqr8 | 0.809 | -0.306 | -1.920 |
| 235587 | Parp3 | 1.273 | 0.348 | 2.157 |
| 18536 | Pcm1 | 1.323 | 0.404 | 2.246 |
| 18577 | Pde4a | 0.812 | -0.301 | -1.903 |
| 18578 | Pde4b | 0.812 | -0.301 | -1.586 |
| 18583 | Pde7a | 0.830 | -0.268 | -1.656 |
| 14827 | Pdia3 | 0.812 | -0.300 | -1.829 |
| 71853 | Pdia6 | 0.781 | -0.356 | -1.732 |
| 230809 | Pdik1l | 0.820 | -0.286 | -1.901 |
| 56376 | Pdlim5 | 0.815 | -0.295 | -1.896 |
| 102693 | Phldb1 | 1.241 | 0.311 | 2.093 |
| 18719 | Pip5k1a | 0.827 | -0.274 | -1.646 |
| 74055 | Plce1 | 0.807 | -0.309 | -1.923 |
| 403178 | Plcxd1 | 1.251 | 0.323 | 2.101 |
| 239318 | Plcxd3 | 0.782 | -0.354 | -1.963 |
| 213556 | Plekhh2 | 0.832 | -0.266 | -1.654 |
| 20873 | Plk4 | 1.278 | 0.354 | 2.215 |
| 67801 | Pllp | 1.289 | 0.366 | 2.130 |
| 70310 | Plscr3 | 1.266 | 0.340 | 2.085 |
| 107939 | Pom121 | 0.817 | -0.291 | -1.719 |
| 18986 | Pou2f1 | 0.810 | -0.304 | -1.791 |
| 67916 | Ppap2b | 0.784 | -0.351 | -2.088 |
| 170826 | Ppargc1b | 0.749 | -0.417 | -2.262 |
| 76497 | Ppp1r11 | 0.816 | -0.294 | -1.845 |
| 333654 | Ppp1r13l | 0.820 | -0.287 | -1.903 |
| 53412 | Ppp1r3c | 0.780 | -0.359 | -1.912 |
| 26931 | Ppp2r5c | 1.262 | 0.336 | 2.126 |
| 213760 | Prepl | 1.298 | 0.376 | 2.078 |
| 108079 | Prkaa2 | 1.293 | 0.371 | 2.069 |
| 19088 | Prkar2b | 1.283 | 0.359 | 2.070 |
| 73728 | Psd | 1.291 | 0.368 | 2.130 |
| 69077 | Psmd11 | 0.775 | -0.367 | -2.234 |
| 23997 | Psmd13 | 1.254 | 0.326 | 2.082 |
| 66645 | Pspc1 | 1.303 | 0.382 | 2.142 |
| 19201 | Pstpip2 | 1.269 | 0.344 | 2.177 |
| 19207 | Ptch2 | 0.799 | -0.323 | -1.833 |
| 19246 | Ptpn1 | 0.754 | -0.407 | -2.217 |
| 19268 | Ptprf | 0.815 | -0.296 | -1.789 |
| 67369 | Qpctl | 1.296 | 0.374 | 2.348 |
| 96938 | R74740 | 1.242 | 0.313 | 2.133 |
| 19334 | Rab22a | 0.817 | -0.292 | -1.773 |
| 19337 | Rab33a | 1.276 | 0.352 | 2.165 |
| 19340 | Rab3d | 0.792 | -0.337 | -1.964 |
| 19359 | Rad23b | 1.291 | 0.368 | 2.193 |
| 19415 | Rasal1 | 1.312 | 0.392 | 2.203 |
| 70727 | Rasgef1a | 1.387 | 0.472 | 2.220 |
| 320292 | Rasgef1b | 1.285 | 0.362 | 2.216 |
| 192678 | Rassf3 | 1.291 | 0.368 | 2.136 |
| 93686 | Rbm9 | 0.744 | -0.427 | -1.976 |
| 17252 | Rdh11 | 1.300 | 0.378 | 2.172 |
| 56533 | Rgs17 | 1.309 | 0.388 | 2.141 |
| 56470 | Rgs19 | 1.275 | 0.350 | 2.124 |
| 320360 | Ric3 | 1.309 | 0.388 | 2.298 |
| 67045 | Riok2 | 1.313 | 0.393 | 2.258 |
| 66878 | Riok3 | 1.261 | 0.335 | 2.063 |
| 328234 | Rnf182 | 0.766 | -0.384 | -2.189 |
| 230257 | Rod1 | 1.294 | 0.372 | 2.206 |
| 19893 | Rpgr | 1.322 | 0.403 | 2.232 |
| 77945 | Rpgrip1 | 0.784 | -0.351 | -1.946 |
| 382985 | Rrm2b | 1.326 | 0.407 | 2.140 |
| 20168 | Rtn3 | 1.297 | 0.375 | 2.117 |
| 70432 | Rufy2 | 1.315 | 0.395 | 2.190 |
| 12395 | Runx1t1 | 0.794 | -0.333 | -1.836 |
| 69519 | Rwdd2 | 1.262 | 0.336 | 2.117 |
| 20199 | S100a5 | 0.664 | -0.591 | -1.957 |
| 104175 | Sbk1 | 0.752 | -0.411 | -1.741 |
| 110876 | Scn2a1 | 0.798 | -0.325 | -1.848 |
| 72821 | Scn2b | 1.304 | 0.383 | 2.225 |
| 20284 | Scrg1 | 0.788 | -0.344 | -1.912 |
| 240880 | Scyl3 | 1.293 | 0.371 | 2.218 |
| 64136 | Sdf2l1 | 0.786 | -0.348 | -1.974 |
| 20338 | Sel1h | 0.789 | -0.341 | -2.011 |
| 20349 | Sema3e | 0.797 | -0.327 | -1.992 |
| 378702 | Serf2 | 0.822 | -0.283 | -1.804 |
| 12406 | Serpinh1 | 0.787 | -0.346 | -1.950 |
| 58172 | Sertad2 | 0.827 | -0.274 | -1.702 |
| 72895 | Setd5 | 1.287 | 0.364 | 2.238 |
| 58234 | Shank3 | 0.779 | -0.361 | -2.189 |
| 20425 | Shmt1 | 1.269 | 0.344 | 2.104 |
| 20467 | Sin3b | 1.291 | 0.369 | 2.207 |
| 68346 | Sirt5 | 1.254 | 0.326 | 2.062 |
| 21402 | Skp1a | 1.385 | 0.470 | 2.432 |
| 27401 | Skp2 | 1.278 | 0.354 | 2.111 |
| 56643 | Slc15a1 | 1.292 | 0.370 | 2.202 |
| 20501 | Slc16a1 | 1.271 | 0.346 | 2.085 |
| 20512 | Slc1a3 | 0.802 | -0.318 | -1.940 |
| 214663 | Slc25a29 | 0.830 | -0.268 | -1.734 |
| 192287 | Slc25a36 | 1.309 | 0.388 | 2.240 |
| 20528 | Slc2a4 | 0.799 | -0.324 | -1.907 |
| 277468 | Slc39a12 | 0.787 | -0.345 | -2.132 |
| 215113 | Slc43a2 | 1.318 | 0.398 | 2.287 |
| 21366 | Slc6a6 | 1.363 | 0.447 | 2.460 |
| 50934 | Slc7a8 | 1.297 | 0.375 | 2.231 |
| 65962 | Slc9a3r2 | 0.816 | -0.293 | -1.665 |
| 28250 | Slco1a4 | 1.252 | 0.324 | 2.131 |
| 17128 | Smad4 | 0.802 | -0.318 | -1.838 |
| 12180 | Smyd1 | 0.800 | -0.322 | -1.982 |
| 20656 | Sod2 | 1.341 | 0.423 | 2.459 |
| 50817 | Solh | 0.798 | -0.325 | -1.917 |
| 20411 | Sorbs1 | 0.779 | -0.361 | -1.938 |
| 58178 | Sorcs1 | 1.305 | 0.384 | 2.303 |
| 20666 | Sox11 | 0.778 | -0.363 | -2.162 |
| 20672 | Sox18 | 0.789 | -0.341 | -2.122 |
| 20688 | Sp4 | 1.296 | 0.374 | 2.066 |
| 20692 | Sparc | 0.756 | -0.403 | -2.405 |
| 57815 | Spata5 | 0.819 | -0.288 | -1.785 |
| 20745 | Spock1 | 1.291 | 0.368 | 2.151 |
| 114715 | Spred1 | 0.796 | -0.329 | -1.928 |
| 114716 | Spred2 | 0.776 | -0.365 | -1.933 |
| 20787 | Srebf1 | 1.246 | 0.317 | 2.147 |
| 14270 | Srgap2 | 0.825 | -0.278 | -1.778 |
| 66661 | Srp72 | 1.315 | 0.395 | 2.144 |
| 20823 | Ssb | 1.258 | 0.331 | 2.096 |
| 70599 | Ssfa2 | 0.824 | -0.280 | -1.770 |
| 20604 | Sst | 1.268 | 0.342 | 2.088 |
| 20848 | Stat3 | 0.808 | -0.308 | -1.710 |
| 20867 | Stip1 | 0.786 | -0.347 | -1.961 |
| 59041 | Stk25 | 1.314 | 0.394 | 2.109 |
| 69106 | Stoml1 | 1.263 | 0.337 | 2.115 |
| 71069 | Stox2 | 0.791 | -0.339 | -1.995 |
| 20913 | Stxbp4 | 0.786 | -0.347 | -2.069 |
| 240725 | Sulf1 | 0.833 | -0.264 | -1.811 |
| 104015 | Synj1 | 1.366 | 0.450 | 2.207 |
| 24071 | Synj2bp | 0.752 | -0.411 | -2.093 |
| 238266 | Syt16 | 1.308 | 0.387 | 2.179 |
| 83671 | Sytl2 | 1.289 | 0.366 | 2.237 |
| 216965 | Taok1 | 0.773 | -0.372 | -1.915 |
| 71807 | Tarsl1 | 1.323 | 0.404 | 2.379 |
| 75812 | Tasp1 | 1.283 | 0.359 | 2.112 |
| 67046 | Tbc1d7 | 1.269 | 0.344 | 2.174 |
| 70571 | Tcerg1l | 0.803 | -0.316 | -1.973 |
| 21415 | Tcf3 | 0.799 | -0.323 | -1.978 |
| 21416 | Tcf7l2 | 1.352 | 0.435 | 2.231 |
| 21580 | Tcrb-J | 1.280 | 0.356 | 2.063 |
| 102791 | Tcta | 1.276 | 0.352 | 2.200 |
| 21678 | Tead3 | 0.808 | -0.308 | -1.839 |
| 21766 | Tex261 | 0.818 | -0.290 | -1.809 |
| 22134 | Tgoln1 | 0.828 | -0.273 | -1.795 |
| 327987 | Thrap1 | 0.802 | -0.319 | -1.922 |
| 76199 | Thrap2 | 0.770 | -0.377 | -2.163 |
| 105663 | Thtpa | 1.269 | 0.344 | 2.128 |
| 21838 | Thy1 | 1.295 | 0.373 | 2.192 |
| 194655 | Tieg3 | 1.284 | 0.361 | 2.252 |
| 110595 | Timp4 | 0.797 | -0.328 | -1.646 |
| 21881 | Tkt | 1.249 | 0.321 | 2.070 |
| 319880 | Tmcc3 | 0.747 | -0.421 | -2.323 |
| 67356 | Tmco5 | 1.277 | 0.353 | 2.087 |
| 56363 | Tmeff2 | 1.262 | 0.336 | 2.065 |
| 226115 | Tmem10 | 1.504 | 0.589 | 2.635 |
| 380967 | Tmem106c | 1.301 | 0.380 | 2.125 |
| 211986 | Tmem18 | 1.270 | 0.345 | 2.208 |
| 67878 | Tmem33 | 0.809 | -0.305 | -1.805 |
| 68777 | Tmem53 | 1.256 | 0.329 | 2.124 |
| 319939 | Tns3 | 0.801 | -0.320 | -1.857 |
| 252838 | Tox | 1.327 | 0.408 | 2.470 |
| 22017 | Tpmt | 0.815 | -0.296 | -1.930 |
| 22031 | Traf3 | 0.812 | -0.300 | -1.800 |
| 217410 | Trib2 | 0.808 | -0.307 | -1.857 |
| 56631 | Trim17 | 0.811 | -0.303 | -1.875 |
| 94090 | Trim9 | 0.759 | -0.397 | -1.905 |
| 83925 | Trps1 | 0.781 | -0.357 | -1.921 |
| 70747 | Tspan2 | 1.341 | 0.423 | 2.386 |
| 380752 | Tssc1 | 1.300 | 0.379 | 2.306 |
| 234875 | Ttc13 | 1.353 | 0.436 | 2.163 |
| 22130 | Ttf1 | 0.815 | -0.295 | -2.018 |
| 319953 | Ttll1 | 1.294 | 0.372 | 2.204 |
| 53857 | Tuba8 | 0.807 | -0.310 | -1.914 |
| 80286 | Tusc3 | 1.247 | 0.319 | 2.125 |
| 28071 | Twistnb | 1.277 | 0.353 | 2.176 |
| 107652 | Uap1 | 1.271 | 0.346 | 2.064 |
| 76577 | Ubxd8 | 0.818 | -0.290 | -1.766 |
| 109113 | Uhrf2 | 0.832 | -0.265 | -1.657 |
| 243537 | Uroc1 | 0.822 | -0.283 | -1.753 |
| 53376 | Usp2 | 1.275 | 0.351 | 2.094 |
| 105372 | Utp15 | 1.294 | 0.372 | 2.120 |
| 22320 | Vamp8 | 0.823 | -0.281 | -1.688 |
| 22329 | Vcam1 | 0.847 | -0.240 | -1.609 |
| 22350 | Vil2 | 0.815 | -0.296 | -1.773 |
| 22352 | Vim | 0.760 | -0.395 | -1.951 |
| 22359 | Vldlr | 0.756 | -0.404 | -2.280 |
| 71732 | Vps11 | 1.297 | 0.375 | 2.358 |
| 83669 | Wdr6 | 1.374 | 0.458 | 2.549 |
| 71856 | Wfdc3 | 0.829 | -0.270 | -1.689 |
| 52639 | Wipi1 | 1.315 | 0.395 | 2.169 |
| 22421 | Wnt7a | 0.827 | -0.274 | -1.758 |
| 78889 | Wsb1 | 1.288 | 0.365 | 2.069 |
| 22433 | Xbp1 | 0.780 | -0.359 | -1.758 |
| 22627 | Ywhae | 1.319 | 0.399 | 2.098 |
| 229603 | Za20d1 | 0.795 | -0.331 | -1.863 |
| 235320 | Zbtb16 | 0.776 | -0.366 | -1.759 |
| 56490 | Zbtb20 | 0.768 | -0.381 | -1.944 |
| 109929 | Zbtb25 | 1.261 | 0.335 | 2.074 |
| 70579 | Zc3h11a | 0.792 | -0.337 | -1.978 |
| 319885 | Zcchc7 | 0.764 | -0.388 | -1.993 |
| 22673 | Zfp185 | 0.801 | -0.321 | -1.908 |
| 59057 | Zfp191 | 0.798 | -0.326 | -2.049 |
| 81018 | Zfp313 | 1.315 | 0.395 | 2.177 |
| 218100 | Zfp322a | 0.815 | -0.296 | -1.673 |
| 94187 | Zfp423 | 1.255 | 0.328 | 2.224 |
| 242466 | Zfp462 | 0.804 | -0.315 | -1.876 |
| 67370 | Zfp606 | 1.286 | 0.363 | 2.169 |
| 270210 | Zfp651 | 0.788 | -0.343 | -2.193 |
| 22781 | Zfpn1a4 | 1.283 | 0.359 | 2.207 |
| 170737 | Znrf1 | 0.812 | -0.300 | -1.781 |
| 360216 | Zranb1 | 1.273 | 0.348 | 2.145 |

Table S4 Complete list of significantly regulated genes in double-transgenic dnADAM10/APP_[V717I]_ mice (three female and three male 5 months old, dnADAM10/APP_[V717I]_ mice as well as APP_[V717I]_ mice)

| **Table S4 Gene ID** | **Gene Symbol** | **Fold change** | **Log Ratio** | **d-value** |
| --- | --- | --- | --- | --- |
| 71678 | 0610010K06Rik | 1.349 | 0.432 | 3.536 |
| 66060 | 0610010O12Rik | 0.735 | -0.444 | -3.730 |
| 71691 | 0710005I19Rik | 0.711 | -0.493 | -3.204 |
| 75404 | 1100001E04Rik | 0.699 | -0.517 | -3.153 |
| 68497 | 1110018G07Rik | 1.361 | 0.445 | 3.321 |
| 68625 | 1110020C03Rik | 0.742 | -0.431 | -3.146 |
| 66152 | 1110020P15Rik | 0.770 | -0.378 | -2.957 |
| 68796 | 1110039B18Rik | 0.762 | -0.393 | -2.958 |
| 68795 | 1110059H15Rik | 1.394 | 0.479 | 3.408 |
| 268390 | 1110064P04Rik | 0.743 | -0.429 | -3.204 |
| 71798 | 1110069O07Rik | 0.745 | -0.424 | -3.174 |
| 67458 | 1200007D18Rik | 0.724 | -0.466 | -2.955 |
| 74152 | 1300002K09Rik | 0.680 | -0.557 | -3.461 |
| 72019 | 1500005C15Rik | 0.722 | -0.469 | -3.147 |
| 76566 | 1500005K14Rik | 0.724 | -0.466 | -3.268 |
| 66498 | 1500034J01Rik | 0.763 | -0.390 | -2.871 |
| 71995 | 1600014E20Rik | 0.715 | -0.484 | -3.253 |
| 66323 | 1700001K19Rik | 0.669 | -0.580 | -4.001 |
| 71837 | 1700003E16Rik | 0.747 | -0.421 | -3.011 |
| 74213 | 1700009P03Rik | 1.382 | 0.467 | 3.390 |
| 69341 | 1700010B09Rik | 0.652 | -0.618 | -4.138 |
| 67078 | 1700012G19Rik | 0.770 | -0.378 | -2.984 |
| 73281 | 1700023G09Rik | 0.722 | -0.469 | -3.255 |
| 66337 | 1700025K23Rik | 1.344 | 0.427 | 3.333 |
| 74266 | 1700039M15Rik | 0.744 | -0.427 | -3.486 |
| 73327 | 1700040I03Rik | 1.382 | 0.467 | 3.616 |
| 68221 | 1700049M11Rik | 0.739 | -0.436 | -2.997 |
| 73367 | 1700052M09Rik | 0.750 | -0.415 | -3.219 |
| 67925 | 1700066D14Rik | 0.655 | -0.610 | -3.541 |
| 73520 | 1700084K02Rik | 0.676 | -0.564 | -3.936 |
| 78469 | 1700090G07Rik | 0.760 | -0.396 | -2.894 |
| 74288 | 1700095G12Rik | 0.731 | -0.453 | -2.971 |
| 73589 | 1700101I19Rik | 0.752 | -0.412 | -3.039 |
| 66274 | 1810012P15Rik | 1.379 | 0.464 | 3.383 |
| 69119 | 1810021M19Rik | 0.727 | -0.460 | -3.273 |
| 380773 | 1810035L17Rik | 1.354 | 0.437 | 3.290 |
| 72123 | 2010109K11Rik | 0.746 | -0.423 | -3.007 |
| 68029 | 2010203O03Rik | 1.375 | 0.459 | 3.308 |
| 67098 | 2210403K04Rik | 1.433 | 0.519 | 3.653 |
| 381845 | 2310014L17Rik | 0.756 | -0.404 | -3.097 |
| 69698 | 2310046K01Rik | 0.747 | -0.420 | -2.927 |
| 66965 | 2310061F22Rik | 1.369 | 0.453 | 3.434 |
| 74504 | 2410018C17Rik | 0.766 | -0.385 | -2.855 |
| 70229 | 2410024N18Rik | 1.365 | 0.449 | 3.298 |
| 76788 | 2410127E18Rik | 0.728 | -0.457 | -3.583 |
| 67968 | 2410146L05Rik | 0.649 | -0.623 | -4.238 |
| 72190 | 2510009E07Rik | 1.458 | 0.544 | 3.652 |
| 70292 | 2600003E23Rik | 1.350 | 0.433 | 3.398 |
| 72386 | 2610035D17Rik | 0.709 | -0.496 | -3.670 |
| 67163 | 2610204L23Rik | 1.393 | 0.478 | 3.337 |
| 72514 | 2610306H15Rik | 1.383 | 0.468 | 3.372 |
| 72543 | 2610528K11Rik | 1.378 | 0.463 | 3.538 |
| 108897 | 2810003C17Rik | 0.667 | -0.584 | -4.256 |
| 72677 | 2810049E08Rik | 1.445 | 0.531 | 3.510 |
| 77994 | 2810055G20Rik | 1.441 | 0.527 | 3.317 |
| 67607 | 2810426N06Rik | 1.417 | 0.503 | 3.377 |
| 72883 | 2900035I09Rik | 0.759 | -0.398 | -2.904 |
| 70354 | 3110001I20Rik | 1.437 | 0.523 | 3.431 |
| 68038 | 3110023E09Rik | 1.381 | 0.466 | 3.553 |
| 73168 | 3110027N22Rik | 1.400 | 0.485 | 3.630 |
| 73219 | 3110080E11Rik | 1.476 | 0.562 | 3.295 |
| 103861 | 4632423N09Rik | 1.372 | 0.456 | 3.313 |
| 70887 | 4921520P21Rik | 1.386 | 0.471 | 3.669 |
| 68187 | 4921533L14Rik | 1.390 | 0.475 | 3.450 |
| 75782 | 4930431B11Rik | 1.414 | 0.500 | 3.498 |
| 74692 | 4930442P07Rik | 0.759 | -0.397 | -2.995 |
| 74652 | 4930453J04Rik | 0.702 | -0.510 | -4.145 |
| 74906 | 4930478M09Rik | 0.742 | -0.431 | -3.610 |
| 74706 | 4930507D05Rik | 0.755 | -0.406 | -3.044 |
| 74722 | 4930517J16Rik | 0.676 | -0.564 | -4.401 |
| 74716 | 4930521I23Rik | 0.741 | -0.432 | -3.234 |
| 406209 | 4930524O07Rik | 1.375 | 0.459 | 3.406 |
| 78774 | 4930529M08Rik | 0.755 | -0.405 | -2.966 |
| 68306 | 4930565N06Rik | 0.736 | -0.442 | -3.010 |
| 75924 | 4930566N20Rik | 0.744 | -0.426 | -3.255 |
| 329782 | 4930570G19Rik | 1.446 | 0.532 | 3.723 |
| 75892 | 4930578I07Rik | 0.760 | -0.396 | -2.860 |
| 67749 | 4930583H14Rik | 0.733 | -0.449 | -3.370 |
| 67723 | 4932415G12Rik | 1.384 | 0.469 | 3.400 |
| 74374 | 4932416N17Rik | 0.751 | -0.414 | -3.089 |
| 74072 | 4933407I05Rik | 0.717 | -0.480 | -3.478 |
| 71088 | 4933412E24Rik | 0.731 | -0.453 | -3.318 |
| 74477 | 4933427D14Rik | 1.405 | 0.491 | 3.296 |
| 71291 | 4933435E02Rik | 0.773 | -0.371 | -2.876 |
| 68144 | 5031426D15Rik | 1.501 | 0.586 | 3.531 |
| 76679 | 5330417H12Rik | 0.733 | -0.449 | -3.534 |
| 73183 | 5430402O13Rik | 0.751 | -0.414 | -2.954 |
| 231214 | 5730509K17Rik | 1.378 | 0.463 | 3.538 |
| 70713 | 6330416L11Rik | 1.408 | 0.494 | 3.425 |
| 236790 | 6330505F04Rik | 1.412 | 0.498 | 3.327 |
| 108667 | 6330549H03Rik | 1.358 | 0.442 | 3.384 |
| 103012 | 6720401G13Rik | 1.554 | 0.636 | 3.955 |
| 399579 | 6720462K09Rik | 1.459 | 0.545 | 3.314 |
| 320339 | 6720469N11Rik | 0.758 | -0.399 | -2.951 |
| 109272 | 8030451F13Rik | 0.743 | -0.428 | -3.078 |
| 213393 | 8430408G22Rik | 0.748 | -0.418 | -3.041 |
| 71523 | 8430429K09Rik | 0.676 | -0.565 | -3.712 |
| 78103 | 8430431K14Rik | 0.731 | -0.453 | -3.154 |
| 77909 | 9230119C12Rik | 0.744 | -0.427 | -3.329 |
| 320227 | 9330121K16Rik | 1.367 | 0.451 | 3.298 |
| 320377 | 9330175E14Rik | 0.763 | -0.391 | -2.949 |
| 77301 | 9430031J08Rik | 0.756 | -0.404 | -3.038 |
| 102493 | 9630007J19Rik | 1.433 | 0.519 | 3.389 |
| 330166 | A230057G18Rik | 1.487 | 0.572 | 3.353 |
| 320270 | A230058F20Rik | 1.468 | 0.554 | 3.377 |
| 225583 | A730017C20Rik | 1.380 | 0.465 | 3.408 |
| 319738 | A730020M07Rik | 0.750 | -0.415 | -3.020 |
| 320317 | A830010M09Rik | 1.419 | 0.505 | 3.386 |
| 414093 | A830082N09Rik | 1.414 | 0.500 | 3.517 |
| 77796 | A930009E05Rik | 1.369 | 0.453 | 3.377 |
| 213006 | A930031D07Rik | 1.460 | 0.546 | 3.719 |
| 268860 | Abat | 1.503 | 0.588 | 3.791 |
| 11421 | Ace | 0.746 | -0.423 | -3.311 |
| 226977 | Actr1b | 1.369 | 0.453 | 3.357 |
| 74117 | Actr3 | 1.393 | 0.478 | 3.380 |
| 11487 | Adam10 | 1.690 | 0.757 | 5.444 |
| 208936 | Adamts18 | 0.752 | -0.411 | -2.973 |
| 330119 | Adamts3 | 1.379 | 0.464 | 3.523 |
| 11518 | Add1 | 1.396 | 0.481 | 3.408 |
| 68465 | Adipor2 | 0.745 | -0.425 | -3.153 |
| 11554 | Adrb1 | 1.365 | 0.449 | 3.354 |
| 280662 | Afm | 0.702 | -0.510 | -3.493 |
| 67269 | Agtpbp1 | 1.397 | 0.482 | 3.311 |
| 226747 | Ahctf1 | 1.479 | 0.565 | 3.673 |
| 106877 | AI173486 | 1.376 | 0.460 | 3.398 |
| 233066 | AI428936 | 0.733 | -0.449 | -3.482 |
| 330941 | AI593442 | 1.393 | 0.478 | 3.448 |
| 277463 | AI790205 | 0.721 | -0.471 | -3.671 |
| 268354 | AI851790 | 1.377 | 0.462 | 3.348 |
| 101694 | AI854517 | 1.379 | 0.464 | 3.387 |
| 69113 | Alkbh3 | 1.363 | 0.447 | 3.356 |
| 11602 | Angpt4 | 0.730 | -0.455 | -2.962 |
| 68839 | Ankrd46 | 1.388 | 0.473 | 3.574 |
| 11768 | Ap1m2 | 0.726 | -0.461 | -3.101 |
| 11774 | Ap3b1 | 1.372 | 0.456 | 3.444 |
| 11792 | Apex1 | 1.499 | 0.584 | 3.820 |
| 226548 | Aph1a | 0.724 | -0.466 | -3.127 |
| 70497 | Arhgap17 | 1.386 | 0.471 | 3.508 |
| 74125 | Armc8 | 1.376 | 0.461 | 3.360 |
| 74840 | Armet | 0.699 | -0.516 | -3.159 |
| 68089 | Arpc4 | 1.350 | 0.433 | 3.338 |
| 215705 | Arrdc1 | 0.743 | -0.428 | -3.011 |
| 105171 | Arrdc3 | 1.397 | 0.482 | 3.309 |
| 108888 | Atad3a | 1.371 | 0.455 | 3.534 |
| 11931 | Atp1b1 | 1.412 | 0.498 | 3.365 |
| 11938 | Atp2a2 | 1.456 | 0.542 | 3.900 |
| 140494 | Atp6v0a4 | 0.762 | -0.392 | -2.972 |
| 242341 | Atp6v0d2 | 0.755 | -0.405 | -2.910 |
| 54667 | Atp8b2 | 1.369 | 0.453 | 3.374 |
| 11998 | Avp | 1.500 | 0.585 | 3.375 |
| 78658 | B130055D15Rik | 1.373 | 0.457 | 3.395 |
| 102941 | B630019K06Rik | 1.476 | 0.562 | 3.804 |
| 320085 | B830012L14Rik | 1.462 | 0.548 | 3.634 |
| 210933 | Bai3 | 1.378 | 0.463 | 3.343 |
| 108100 | Baiap2 | 0.760 | -0.396 | -2.853 |
| 70508 | Bbx | 0.691 | -0.534 | -3.237 |
| 545670 | BC013476 | 0.758 | -0.399 | -3.050 |
| 213056 | BC049806 | 0.732 | -0.450 | -2.911 |
| 29815 | Bcar3 | 0.766 | -0.385 | -3.066 |
| 503859 | BF642829 | 0.711 | -0.492 | -2.951 |
| 12122 | Bid | 1.381 | 0.466 | 3.765 |
| 75388 | Boll | 0.745 | -0.425 | -2.865 |
| 215387 | Brrn1 | 0.746 | -0.422 | -2.987 |
| 207425 | Brwd2 | 1.367 | 0.451 | 3.347 |
| 67832 | Bxdc2 | 1.481 | 0.567 | 3.658 |
| 109357 | C030004M05Rik | 1.353 | 0.436 | 3.338 |
| 77521 | C130038G02Rik | 0.736 | -0.442 | -3.168 |
| 328322 | C230069N13 | 1.431 | 0.517 | 3.468 |
| 399604 | C530014P21Rik | 0.742 | -0.430 | -3.385 |
| 104248 | Cabin1 | 1.411 | 0.497 | 3.749 |
| 12293 | Cacna2d1 | 1.460 | 0.546 | 4.086 |
| 12308 | Calb2 | 0.762 | -0.392 | -3.021 |
| 67488 | Calcoco1 | 1.434 | 0.520 | 3.383 |
| 12374 | Casr | 0.752 | -0.411 | -2.889 |
| 76380 | Ccdc46 | 0.769 | -0.379 | -2.882 |
| 442829 | Ccin | 0.747 | -0.421 | -3.215 |
| 20303 | Ccl4 | 0.755 | -0.405 | -2.995 |
| 12772 | Ccr2 | 0.648 | -0.625 | -3.824 |
| 54219 | Cd320 | 0.725 | -0.463 | -3.299 |
| 26367 | Ceacam2 | 0.757 | -0.401 | -2.939 |
| 12616 | Cenpb | 0.763 | -0.391 | -3.029 |
| 78618 | Centb2 | 1.411 | 0.497 | 3.325 |
| 12626 | Cetn3 | 1.436 | 0.522 | 3.499 |
| 71375 | Ches1 | 0.764 | -0.389 | -2.902 |
| 75608 | Chmp4b | 1.371 | 0.455 | 3.456 |
| 66917 | Chordc1 | 0.697 | -0.521 | -3.155 |
| 12696 | Cirbp | 1.811 | 0.857 | 4.427 |
| 12723 | Clcn1 | 0.733 | -0.448 | -3.612 |
| 12741 | Cldn5 | 0.724 | -0.465 | -3.397 |
| 94040 | Clmn | 0.747 | -0.420 | -2.944 |
| 64085 | Clstn2 | 1.414 | 0.500 | 3.515 |
| 12757 | Clta | 1.564 | 0.645 | 4.042 |
| 94220 | Cnnm4 | 0.735 | -0.445 | -3.317 |
| 66797 | Cntnap2 | 1.379 | 0.464 | 3.444 |
| 76332 | Cog2 | 1.403 | 0.489 | 3.501 |
| 233824 | Cog7 | 1.402 | 0.487 | 3.670 |
| 73368 | Col20a1 | 0.707 | -0.501 | -3.578 |
| 76501 | Commd9 | 1.399 | 0.484 | 3.463 |
| 23790 | Coro1c | 1.369 | 0.453 | 3.426 |
| 12868 | Cox8a | 0.776 | -0.366 | -2.966 |
| 67579 | Cpeb4 | 1.391 | 0.476 | 3.392 |
| 12890 | Cplx2 | 1.392 | 0.477 | 3.581 |
| 211232 | Cpne9 | 1.393 | 0.478 | 3.401 |
| 12955 | Cryab | 0.711 | -0.492 | -3.095 |
| 66609 | Cryzl1 | 0.774 | -0.369 | -2.941 |
| 12977 | Csf1 | 0.767 | -0.383 | -2.920 |
| 12978 | Csf1r | 0.765 | -0.386 | -2.946 |
| 13000 | Csnk2a2 | 1.384 | 0.469 | 3.367 |
| 30785 | Cttnbp2 | 1.426 | 0.512 | 3.534 |
| 71745 | Cul2 | 1.402 | 0.488 | 3.738 |
| 13047 | Cutl1 | 1.435 | 0.521 | 3.339 |
| 244672 | Cwf19l2 | 1.409 | 0.495 | 3.368 |
| 56066 | Cxcl11 | 0.760 | -0.395 | -2.953 |
| 20315 | Cxcl12 | 1.373 | 0.457 | 3.359 |
| 225912 | Cybasc3 | 0.746 | -0.422 | -3.136 |
| 546001 | D030022P06Rik | 0.753 | -0.409 | -2.922 |
| 228361 | D030051N19Rik | 1.311 | 0.391 | 3.296 |
| 52668 | D12Ertd647e | 0.747 | -0.420 | -3.248 |
| 71919 | D15Ertd682e | 1.381 | 0.466 | 3.835 |
| 51873 | D2Ertd127e | 0.750 | -0.415 | -3.031 |
| 51944 | D2Ertd750e | 0.744 | -0.426 | -3.225 |
| 320461 | D430033H22Rik | 1.475 | 0.561 | 3.810 |
| 52120 | D8Ertd354e | 1.347 | 0.430 | 3.359 |
| 56710 | Dbccr1 | 1.444 | 0.530 | 3.737 |
| 13170 | Dbp | 1.696 | 0.762 | 4.584 |
| 195208 | Dcdc2 | 0.690 | -0.536 | -3.198 |
| 212880 | Ddx46 | 1.402 | 0.487 | 3.566 |
| 13371 | Dio2 | 0.708 | -0.498 | -2.871 |
| 208666 | Diras1 | 0.754 | -0.408 | -2.889 |
| 68203 | Diras2 | 1.384 | 0.469 | 3.331 |
| 224997 | Dlgap1 | 1.417 | 0.503 | 3.720 |
| 13418 | Dnajc1 | 0.559 | -0.840 | -4.684 |
| 75563 | Dnali1 | 0.752 | -0.411 | -3.077 |
| 13429 | Dnm1 | 1.380 | 0.465 | 3.452 |
| 74006 | Dnm1l | 1.392 | 0.477 | 3.381 |
| 60425 | Doc2g | 0.711 | -0.492 | -3.258 |
| 67299 | Dock7 | 1.322 | 0.403 | 3.321 |
| 76088 | Dock8 | 0.741 | -0.433 | -2.881 |
| 244745 | Dpy19l1 | 1.421 | 0.507 | 3.675 |
| 13527 | Dtna | 1.338 | 0.420 | 3.294 |
| 319689 | E030031F02Rik | 1.381 | 0.466 | 3.318 |
| 99358 | E130013N09Rik | 1.654 | 0.726 | 4.143 |
| 623474 | E130016E03Rik | 0.705 | -0.505 | -3.764 |
| 13647 | Egfbp2 | 0.643 | -0.638 | -4.649 |
| 15568 | Elavl1 | 1.401 | 0.486 | 3.451 |
| 13731 | Emp2 | 0.728 | -0.458 | -3.370 |
| 13805 | Eng | 0.762 | -0.393 | -3.149 |
| 13813 | Eomes | 0.704 | -0.506 | -3.121 |
| 226352 | Epb4.1l5 | 0.736 | -0.443 | -3.225 |
| 53413 | Exoc7 | 1.345 | 0.428 | 3.317 |
| 14073 | Faah | 1.344 | 0.427 | 3.386 |
| 66930 | Fank1 | 0.702 | -0.511 | -3.138 |
| 14114 | Fbln1 | 0.718 | -0.478 | -2.985 |
| 214931 | Fbxl16 | 1.419 | 0.505 | 3.292 |
| 269514 | Fbxl4 | 1.363 | 0.447 | 3.511 |
| 14128 | Fcer2a | 0.677 | -0.563 | -3.796 |
| 58249 | Fibp | 1.371 | 0.455 | 3.526 |
| 94244 | Fkbp6 | 0.739 | -0.436 | -3.138 |
| 55990 | Fmo2 | 0.753 | -0.410 | -3.037 |
| 72007 | Fndc3b | 0.737 | -0.440 | -3.510 |
| 60611 | Foxj2 | 0.746 | -0.423 | -2.931 |
| 320365 | Fry | 1.391 | 0.476 | 3.437 |
| 320267 | Fubp3 | 1.365 | 0.449 | 3.359 |
| 233908 | Fus | 1.429 | 0.515 | 3.404 |
| 14344 | Fut2 | 0.778 | -0.363 | -2.859 |
| 14349 | Fv1 | 0.728 | -0.457 | -3.298 |
| 93739 | Gabarapl2 | 1.395 | 0.480 | 3.411 |
| 23882 | Gadd45g | 1.371 | 0.455 | 3.534 |
| 14423 | Galnt1 | 1.426 | 0.512 | 3.361 |
| 108148 | Galnt2 | 1.364 | 0.448 | 3.428 |
| 99326 | Garnl3 | 1.378 | 0.463 | 3.705 |
| 80909 | Gats | 0.775 | -0.367 | -3.037 |
| 328232 | Gfod1 | 0.738 | -0.438 | -3.397 |
| 207182 | Ggtl3 | 1.452 | 0.538 | 4.118 |
| 170772 | Glcci1 | 1.442 | 0.528 | 3.838 |
| 93683 | Glce | 1.462 | 0.548 | 3.526 |
| 74412 | Gle1l | 1.385 | 0.470 | 3.389 |
| 229599 | Gm129 | 1.495 | 0.580 | 3.860 |
| 195209 | Gm22 | 0.721 | -0.471 | -3.226 |
| 210573 | Gm323 | 0.759 | -0.398 | -2.990 |
| 14706 | Gng4 | 0.733 | -0.448 | -2.914 |
| 230737 | Gnl2 | 1.381 | 0.466 | 3.448 |
| 436090 | Gpr62 | 1.406 | 0.492 | 3.747 |
| 67298 | Gprasp1 | 1.393 | 0.478 | 3.344 |
| 107022 | Gramd3 | 0.694 | -0.528 | -3.698 |
| 17444 | Grap2 | 0.768 | -0.380 | -3.077 |
| 268527 | Greb1 | 0.742 | -0.431 | -2.966 |
| 195733 | Grhl1 | 1.410 | 0.496 | 3.470 |
| 14852 | Gspt1 | 1.354 | 0.437 | 3.303 |
| 14872 | Gstt2 | 1.351 | 0.434 | 3.399 |
| 17263 | Gtl2 | 1.671 | 0.741 | 4.190 |
| 14943 | Gzmf | 0.745 | -0.424 | -3.031 |
| 67552 | H2afy3 | 0.704 | -0.507 | -3.933 |
| 14968 | H2-Ea | 0.703 | -0.509 | -3.642 |
| 56185 | Hao3 | 0.761 | -0.394 | -2.890 |
| 66044 | Hars2 | 1.375 | 0.459 | 3.501 |
| 73389 | Hbp1 | 1.380 | 0.465 | 3.401 |
| 56016 | Hebp2 | 0.709 | -0.496 | -3.444 |
| 238023 | Hexdc | 1.369 | 0.453 | 3.422 |
| 15248 | Hic1 | 0.771 | -0.376 | -2.935 |
| 215114 | Hip1 | 0.772 | -0.373 | -2.971 |
| 15257 | Hipk1 | 0.726 | -0.461 | -3.122 |
| 227399 | Hisppd1 | 1.389 | 0.474 | 3.298 |
| 50926 | Hnrpdl | 1.440 | 0.526 | 3.465 |
| 15387 | Hnrpk | 1.392 | 0.477 | 3.640 |
| 15412 | Hoxb4 | 0.762 | -0.393 | -3.054 |
| 15441 | Hp1bp3 | 1.375 | 0.459 | 3.683 |
| 15452 | Hprt1 | 1.375 | 0.459 | 3.326 |
| 94175 | Hrg | 0.751 | -0.414 | -3.092 |
| 99296 | Hrh3 | 1.435 | 0.521 | 3.684 |
| 15487 | Hsd17b3 | 0.719 | -0.476 | -3.594 |
| 15560 | Htr2c | 1.382 | 0.467 | 3.482 |
| 16017 | Igh-4 | 1.354 | 0.437 | 3.377 |
| 215257 | Il1f9 | 0.706 | -0.503 | -3.808 |
| 16177 | Il1r1 | 0.733 | -0.448 | -3.380 |
| 16190 | Il4ra | 0.745 | -0.424 | -3.307 |
| 16323 | Inhba | 0.744 | -0.426 | -2.892 |
| 16325 | Inhbc | 0.715 | -0.483 | -3.508 |
| 101490 | Inpp5f | 1.405 | 0.491 | 3.433 |
| 231070 | Insig1 | 1.532 | 0.615 | 4.477 |
| 23920 | Insrr | 0.725 | -0.464 | -3.167 |
| 16179 | Irak1 | 1.431 | 0.517 | 3.661 |
| 71927 | Itfg1 | 1.389 | 0.474 | 3.400 |
| 69564 | Itgb1bp3 | 0.720 | -0.473 | -3.143 |
| 67733 | Itgb3bp | 0.730 | -0.454 | -3.256 |
| 16468 | Jarid2 | 1.325 | 0.406 | 3.349 |
| 216850 | Jmjd3 | 0.747 | -0.420 | -2.866 |
| 194952 | Jmjd4 | 1.342 | 0.424 | 3.302 |
| 77035 | Jmjd5 | 0.694 | -0.527 | -3.039 |
| 268345 | Kcnc2 | 1.358 | 0.441 | 3.450 |
| 16549 | Khsrp | 0.777 | -0.364 | -3.044 |
| 110033 | Kif22 | 1.574 | 0.654 | 4.418 |
| 16581 | Kifc2 | 1.388 | 0.473 | 3.442 |
| 16616 | Klk1b21 | 0.714 | -0.487 | -3.711 |
| 80782 | Klrb1d | 0.735 | -0.444 | -3.340 |
| 16593 | Kns2 | 1.353 | 0.436 | 3.319 |
| 79264 | Krit1 | 1.371 | 0.455 | 3.313 |
| 16706 | Ksr1 | 0.775 | -0.368 | -2.877 |
| 16776 | Lama5 | 0.755 | -0.406 | -2.990 |
| 14768 | Lancl1 | 1.335 | 0.417 | 3.335 |
| 102436 | Lars2 | 0.735 | -0.444 | -3.308 |
| 244864 | Layn | 0.742 | -0.431 | -2.879 |
| 320429 | Lba1 | 1.486 | 0.571 | 3.404 |
| 16818 | Lck | 0.698 | -0.519 | -3.204 |
| 232798 | Leng8 | 1.582 | 0.662 | 4.270 |
| 14160 | Lgr5 | 0.772 | -0.374 | -2.908 |
| 22342 | Lin7b | 0.741 | -0.432 | -3.159 |
| 380928 | Lmo7 | 1.389 | 0.474 | 3.298 |
| 232875 | LOC232875 | 1.413 | 0.499 | 3.849 |
| 235580 | LOC235580 | 0.704 | -0.506 | -3.649 |
| 432971 | LOC432971 | 1.518 | 0.602 | 3.611 |
| 435285 | LOC435285 | 0.753 | -0.409 | -2.872 |
| 544864 | LOC544864 | 0.655 | -0.611 | -3.302 |
| 545261 | LOC545261 | 0.739 | -0.437 | -3.459 |
| 77413 | LOC77413 | 1.458 | 0.544 | 3.512 |
| 217366 | Lrrc45 | 1.400 | 0.485 | 3.307 |
| 16987 | Lss | 1.443 | 0.529 | 3.738 |
| 16993 | Lta4h | 0.733 | -0.449 | -3.262 |
| 16998 | Ltbp3 | 1.461 | 0.547 | 3.470 |
| 110454 | Ly6a | 0.749 | -0.416 | -2.985 |
| 94275 | Maged1 | 1.389 | 0.474 | 3.520 |
| 67729 | Mansc1 | 1.421 | 0.507 | 3.628 |
| 208158 | Map6d1 | 1.527 | 0.611 | 4.214 |
| 277010 | Marveld1 | 0.736 | -0.443 | -3.038 |
| 73608 | Marveld3 | 0.683 | -0.551 | -3.782 |
| 217588 | Mbip | 1.524 | 0.608 | 4.353 |
| 270669 | Mbtps2 | 1.404 | 0.490 | 3.331 |
| 17248 | Mdm4 | 1.377 | 0.462 | 3.303 |
| 67943 | Mesdc2 | 0.694 | -0.527 | -3.668 |
| 73490 | Mipol1 | 0.719 | -0.476 | -3.414 |
| 59030 | Mkks | 0.723 | -0.468 | -3.503 |
| 67027 | Mkrn2 | 1.389 | 0.474 | 3.511 |
| 210719 | Mkx | 1.420 | 0.506 | 3.754 |
| 17389 | Mmp16 | 0.714 | -0.487 | -3.431 |
| 24030 | Mrps12 | 0.753 | -0.410 | -3.203 |
| 76784 | Mtif2 | 1.357 | 0.440 | 3.395 |
| 210376 | Mtmr9 | 1.371 | 0.455 | 3.395 |
| 14489 | Mtpn | 1.397 | 0.482 | 3.301 |
| 17830 | Muc10 | 0.758 | -0.400 | -3.160 |
| 17063 | Muc13 | 0.731 | -0.452 | -3.314 |
| 17121 | Mxd3 | 0.761 | -0.394 | -2.995 |
| 228785 | Mylk2 | 0.721 | -0.471 | -3.391 |
| 17910 | Myo15 | 0.753 | -0.410 | -2.883 |
| 17933 | Myt1l | 1.387 | 0.472 | 3.302 |
| 17957 | Napb | 1.427 | 0.513 | 3.378 |
| 103172 | Ndg2 | 0.771 | -0.376 | -2.931 |
| 29812 | Ndrg3 | 1.376 | 0.460 | 3.361 |
| 338352 | Nell1 | 1.410 | 0.496 | 3.339 |
| 29861 | Neud4 | 0.759 | -0.397 | -3.014 |
| 18028 | Nfib | 1.420 | 0.506 | 3.364 |
| 74164 | Nfx1 | 1.404 | 0.490 | 3.882 |
| 75570 | Nhej1 | 0.731 | -0.453 | -3.328 |
| 18087 | Nktr | 1.418 | 0.504 | 3.348 |
| 18092 | Nkx2-6 | 0.694 | -0.526 | -3.570 |
| 216856 | Nlgn2 | 1.380 | 0.465 | 3.418 |
| 18125 | Nos1 | 0.741 | -0.432 | -3.040 |
| 18134 | Nova1 | 1.385 | 0.470 | 3.755 |
| 230103 | Npr2 | 1.422 | 0.508 | 3.417 |
| 217166 | Nr1d1 | 1.445 | 0.531 | 3.799 |
| 21907 | Nr2e1 | 0.702 | -0.511 | -3.426 |
| 14536 | Nr6a1 | 0.736 | -0.443 | -3.368 |
| 98415 | Nucks1 | 1.445 | 0.531 | 3.767 |
| 67725 | Nudt13 | 0.732 | -0.450 | -3.171 |
| 57270 | Olfr1508 | 0.735 | -0.444 | -3.162 |
| 18378 | Omp | 0.756 | -0.403 | -2.860 |
| 18390 | Oprm1 | 0.725 | -0.463 | -3.042 |
| 75735 | Pank1 | 0.667 | -0.584 | -3.659 |
| 170677 | Pcdh21 | 0.614 | -0.703 | -3.902 |
| 18536 | Pcm1 | 1.395 | 0.480 | 3.446 |
| 18549 | Pcsk2 | 1.456 | 0.542 | 3.704 |
| 14827 | Pdia3 | 0.745 | -0.424 | -2.866 |
| 12304 | Pdia4 | 0.703 | -0.508 | -3.207 |
| 230809 | Pdik1l | 0.746 | -0.422 | -3.334 |
| 224824 | Pex6 | 0.753 | -0.409 | -2.897 |
| 223775 | Pim3 | 1.368 | 0.452 | 3.338 |
| 18717 | Pip5k1c | 1.394 | 0.479 | 3.356 |
| 18772 | Pkp1 | 0.694 | -0.528 | -3.801 |
| 56460 | Pkp3 | 0.751 | -0.413 | -2.861 |
| 18805 | Pld1 | 0.743 | -0.429 | -3.052 |
| 102075 | Plekhg4 | 0.700 | -0.514 | -4.009 |
| 211945 | Plekhh1 | 1.370 | 0.454 | 3.337 |
| 67801 | Pllp | 1.404 | 0.490 | 3.437 |
| 18821 | Pln | 0.741 | -0.432 | -3.181 |
| 80905 | Polh | 0.768 | -0.380 | -2.856 |
| 26939 | Polr3e | 1.372 | 0.456 | 3.301 |
| 18987 | Pou2f2 | 0.732 | -0.450 | -3.363 |
| 18992 | Pou3f2 | 0.730 | -0.455 | -3.611 |
| 19014 | Pparbp | 0.748 | -0.419 | -3.030 |
| 19041 | Ppl | 0.756 | -0.404 | -3.193 |
| 76497 | Ppp1r11 | 0.777 | -0.364 | -2.968 |
| 333654 | Ppp1r13l | 0.733 | -0.449 | -3.361 |
| 52432 | Ppp2r2d | 1.387 | 0.472 | 3.782 |
| 233406 | Prc1 | 1.346 | 0.429 | 3.293 |
| 50907 | Preb | 1.489 | 0.574 | 3.773 |
| 74182 | Prei4 | 1.431 | 0.517 | 3.939 |
| 213760 | Prepl | 1.361 | 0.445 | 3.332 |
| 228880 | Prkcbp1 | 1.392 | 0.477 | 3.501 |
| 210673 | Prrt3 | 0.760 | -0.396 | -2.956 |
| 18933 | Prrx1 | 0.752 | -0.411 | -2.927 |
| 70835 | Prss22 | 0.732 | -0.451 | -2.992 |
| 69077 | Psmd11 | 0.745 | -0.425 | -3.331 |
| 228769 | Psmf1 | 1.350 | 0.433 | 3.307 |
| 19207 | Ptch2 | 0.684 | -0.548 | -4.070 |
| 64292 | Ptges | 0.742 | -0.430 | -3.408 |
| 19280 | Ptprs | 1.415 | 0.501 | 3.659 |
| 58998 | Pvrl3 | 1.349 | 0.432 | 3.302 |
| 110078 | Pygb | 1.498 | 0.583 | 4.002 |
| 70536 | Qpct | 1.415 | 0.501 | 3.337 |
| 74998 | Rab11fip2 | 1.391 | 0.476 | 3.442 |
| 19346 | Rab6 | 1.389 | 0.474 | 3.363 |
| 54189 | Rabep1 | 1.377 | 0.462 | 3.307 |
| 67657 | Rabl3 | 1.360 | 0.444 | 3.511 |
| 114714 | Rad51c | 0.775 | -0.368 | -2.858 |
| 231724 | Rad9b | 0.685 | -0.546 | -4.053 |
| 241308 | Ralgps1 | 1.347 | 0.430 | 3.455 |
| 52202 | Rbm34 | 1.405 | 0.491 | 3.611 |
| 19653 | Rbm4 | 0.732 | -0.451 | -3.290 |
| 67010 | Rbm7 | 1.417 | 0.503 | 3.479 |
| 27632 | Rdbp | 1.431 | 0.517 | 3.718 |
| 17252 | Rdh11 | 1.374 | 0.458 | 3.317 |
| 19683 | Rdh16 | 0.747 | -0.421 | -3.138 |
| 110596 | Rgnef | 0.758 | -0.400 | -3.164 |
| 19734 | Rgs16 | 0.749 | -0.416 | -3.214 |
| 212541 | Rho | 0.762 | -0.393 | -3.078 |
| 104384 | Rhox9 | 0.748 | -0.418 | -3.173 |
| 237422 | Ric8b | 1.349 | 0.432 | 3.473 |
| 67504 | Rnf151 | 0.757 | -0.401 | -3.000 |
| 108660 | Rnf187 | 1.564 | 0.645 | 4.038 |
| 19877 | Rock1 | 1.402 | 0.487 | 3.841 |
| 19893 | Rpgr | 1.361 | 0.445 | 3.332 |
| 267019 | Rps15a | 1.358 | 0.441 | 3.383 |
| 12394 | Runx1 | 0.745 | -0.424 | -2.914 |
| 12393 | Runx2 | 0.771 | -0.375 | -2.876 |
| 52850 | Rutbc2 | 0.760 | -0.396 | -3.239 |
| 20184 | Rxrip110 | 1.398 | 0.483 | 3.506 |
| 20199 | S100a5 | 0.578 | -0.792 | -4.089 |
| 20208 | Saa1 | 0.739 | -0.437 | -3.361 |
| 19018 | Scand1 | 0.720 | -0.474 | -3.061 |
| 110876 | Scn2a1 | 1.393 | 0.478 | 3.500 |
| 72821 | Scn2b | 0.755 | -0.405 | -3.176 |
| 74616 | Scrn3 | 0.635 | -0.656 | -3.942 |
| 64136 | Sdf2l1 | 0.693 | -0.529 | -3.523 |
| 237979 | Sdk2 | 0.721 | -0.471 | -3.303 |
| 20343 | Sell | 0.714 | -0.487 | -3.269 |
| 20349 | Sema3e | 0.696 | -0.523 | -3.985 |
| 378702 | Serf2 | 0.769 | -0.379 | -3.037 |
| 71907 | Serpina9 | 0.765 | -0.387 | -2.947 |
| 170742 | Sertad3 | 0.760 | -0.395 | -3.034 |
| 233878 | Sez6l2 | 1.380 | 0.465 | 3.294 |
| 53609 | Sfrs16 | 1.426 | 0.512 | 3.730 |
| 20382 | Sfrs2 | 1.341 | 0.423 | 3.312 |
| 108735 | Sft2d2 | 0.763 | -0.390 | -3.129 |
| 58234 | Shank3 | 0.769 | -0.379 | -2.946 |
| 209011 | Sirt7 | 1.354 | 0.437 | 3.305 |
| 18174 | Slc11a2 | 1.372 | 0.456 | 3.629 |
| 20497 | Slc12a3 | 0.701 | -0.512 | -3.385 |
| 27411 | Slc14a2 | 0.754 | -0.408 | -3.119 |
| 20511 | Slc1a2 | 0.771 | -0.376 | -2.888 |
| 70840 | Slc22a16 | 0.748 | -0.419 | -3.043 |
| 20528 | Slc2a4 | 0.713 | -0.489 | -3.807 |
| 67760 | Slc38a2 | 0.744 | -0.427 | -3.269 |
| 213053 | Slc39a14 | 0.747 | -0.420 | -3.168 |
| 68682 | Slc44a2 | 1.518 | 0.602 | 3.608 |
| 22293 | Slc45a2 | 0.731 | -0.453 | -3.211 |
| 103098 | Slc6a15 | 1.483 | 0.569 | 3.773 |
| 20538 | Slc6a2 | 0.699 | -0.517 | -3.355 |
| 50934 | Slc7a8 | 1.447 | 0.533 | 3.523 |
| 13990 | Smarcad1 | 0.735 | -0.444 | -3.254 |
| 12180 | Smyd1 | 0.749 | -0.416 | -3.154 |
| 102607 | Snx19 | 1.360 | 0.444 | 3.408 |
| 50817 | Solh | 0.762 | -0.392 | -3.154 |
| 20658 | Son | 1.393 | 0.478 | 3.371 |
| 20666 | Sox11 | 0.720 | -0.474 | -3.672 |
| 20682 | Sox9 | 0.754 | -0.408 | -3.089 |
| 70834 | Spag9 | 1.354 | 0.437 | 3.399 |
| 71026 | Speer3 | 0.682 | -0.553 | -3.787 |
| 69611 | Sprrl7 | 0.728 | -0.457 | -3.685 |
| 24066 | Spry4 | 0.738 | -0.438 | -3.045 |
| 268656 | Sptlc1 | 0.733 | -0.449 | -3.158 |
| 109267 | Srcrb4d | 0.650 | -0.621 | -4.390 |
| 217337 | Srp68 | 1.366 | 0.450 | 3.306 |
| 20832 | Ssr4 | 1.370 | 0.454 | 3.495 |
| 70356 | St13 | 0.718 | -0.478 | -3.463 |
| 20867 | Stip1 | 0.711 | -0.492 | -3.421 |
| 106504 | Stk38 | 1.408 | 0.494 | 3.409 |
| 67727 | Stx17 | 1.358 | 0.441 | 3.463 |
| 67955 | Sugt1 | 0.728 | -0.457 | -3.171 |
| 20610 | Sumo3 | 1.399 | 0.484 | 3.344 |
| 96935 | Susd4 | 1.430 | 0.516 | 3.466 |
| 20977 | Syp | 1.448 | 0.534 | 3.608 |
| 20979 | Syt1 | 1.397 | 0.482 | 3.587 |
| 83671 | Sytl2 | 1.439 | 0.525 | 3.787 |
| 21340 | Taf1b | 1.348 | 0.431 | 3.345 |
| 75812 | Tasp1 | 1.382 | 0.467 | 3.355 |
| 66826 | Taz | 1.402 | 0.487 | 3.639 |
| 67046 | Tbc1d7 | 1.326 | 0.407 | 3.290 |
| 70430 | Tbce | 1.345 | 0.428 | 3.370 |
| 21415 | Tcf3 | 0.713 | -0.488 | -3.662 |
| 21678 | Tead3 | 0.710 | -0.494 | -3.797 |
| 21823 | Th | 0.761 | -0.394 | -2.993 |
| 21838 | Thy1 | 1.434 | 0.520 | 3.904 |
| 66131 | Tipin | 1.374 | 0.458 | 3.489 |
| 67623 | Tm7sf3 | 1.384 | 0.469 | 3.417 |
| 226115 | Tmem10 | 1.556 | 0.638 | 3.675 |
| 380967 | Tmem106c | 1.414 | 0.500 | 3.521 |
| 212070 | Tmem12 | 0.741 | -0.432 | -3.167 |
| 69470 | Tmem127 | 1.402 | 0.487 | 3.763 |
| 243339 | Tmem130 | 1.383 | 0.468 | 3.295 |
| 105847 | Tmem153 | 0.739 | -0.437 | -3.325 |
| 22004 | Tpm2 | 0.774 | -0.370 | -2.971 |
| 328833 | Treml2 | 0.724 | -0.465 | -3.489 |
| 56771 | Trfp | 0.689 | -0.537 | -3.862 |
| 56631 | Trim17 | 0.758 | -0.399 | -2.952 |
| 94090 | Trim9 | 0.716 | -0.481 | -2.963 |
| 66745 | Trpd52l3 | 0.747 | -0.421 | -3.349 |
| 21807 | Tsc22d1 | 1.386 | 0.471 | 3.357 |
| 22099 | Tsn | 1.359 | 0.443 | 3.291 |
| 21912 | Tspan7 | 1.384 | 0.469 | 3.688 |
| 52808 | Tspyl2 | 1.370 | 0.454 | 3.476 |
| 319953 | Ttll1 | 1.409 | 0.495 | 3.626 |
| 330010 | Ttll10 | 0.697 | -0.520 | -3.171 |
| 22138 | Ttn | 0.707 | -0.500 | -3.397 |
| 53857 | Tuba8 | 0.753 | -0.410 | -3.105 |
| 28071 | Twistnb | 1.376 | 0.460 | 3.336 |
| 56338 | Txnip | 1.378 | 0.463 | 3.332 |
| 54609 | Ubqln2 | 1.436 | 0.522 | 3.292 |
| 224111 | Ubxd7 | 0.732 | -0.450 | -3.544 |
| 22230 | Ufd1l | 1.355 | 0.438 | 3.317 |
| 243537 | Uroc1 | 0.741 | -0.433 | -3.096 |
| 236733 | Usp11 | 1.356 | 0.439 | 3.326 |
| 59025 | Usp14 | 1.383 | 0.468 | 3.342 |
| 53376 | Usp2 | 1.433 | 0.519 | 3.545 |
| 329908 | Usp24 | 1.375 | 0.459 | 3.301 |
| 57775 | Usp29 | 1.388 | 0.473 | 3.559 |
| 76179 | Usp31 | 0.751 | -0.413 | -3.184 |
| 237898 | Usp32 | 1.358 | 0.442 | 3.344 |
| 76800 | Usp42 | 1.433 | 0.519 | 3.686 |
| 72554 | Utp14a | 0.762 | -0.393 | -2.966 |
| 22320 | Vamp8 | 0.723 | -0.467 | -3.444 |
| 338370 | Vgcnl1 | 1.395 | 0.480 | 3.436 |
| 116733 | Vps4a | 1.341 | 0.423 | 3.444 |
| 72145 | Wdfy3 | 1.465 | 0.551 | 3.388 |
| 244484 | Wdr17 | 1.402 | 0.488 | 3.588 |
| 83669 | Wdr6 | 1.450 | 0.536 | 3.702 |
| 78889 | Wsb1 | 1.544 | 0.627 | 4.298 |
| 16963 | Xcl1 | 0.749 | -0.416 | -3.003 |
| 102680 | Xtrp3s1 | 1.402 | 0.488 | 3.485 |
| 56531 | Ylpm1 | 1.380 | 0.465 | 3.542 |
| 229096 | Ythdf3 | 1.400 | 0.485 | 3.506 |
| 58203 | Zbp1 | 0.753 | -0.409 | -2.963 |
| 229055 | Zbtb10 | 0.760 | -0.395 | -3.160 |
| 109929 | Zbtb25 | 1.390 | 0.475 | 3.736 |
| 67917 | Zcchc3 | 1.394 | 0.479 | 3.386 |
| 22673 | Zfp185 | 0.734 | -0.446 | -3.295 |
| 22694 | Zfp35 | 0.742 | -0.430 | -3.226 |
| 22700 | Zfp40 | 1.373 | 0.457 | 3.343 |
| 98403 | Zfp451 | 1.335 | 0.417 | 3.312 |
| 68036 | Zfp706 | 1.456 | 0.542 | 3.420 |
| 360216 | Zranb1 | 1.392 | 0.477 | 3.371 |
| 71861 | Zswim2 | 0.730 | -0.454 | -3.229 |

Table S5 Commonly regulated genes through ADAM10 overexpression in mono- and double transgenic mice (ADAM10 versus FVB/N (355 genes) compared to ADAM10/APP_[V717I]_ versus APP_[V717I]_ (592 genes)

| **Table S5**  **Gene ID** | **Gene Symbol** | **Gene description** |
| --- | --- | --- |
| 16419 | Itgb5 | INTEGRIN BETA 5 |
| 67684 | 3300001P08Rik | RIKEN CDNA 3300001P08 GENE |
| 72567 | Bclaf1 | RIKEN CDNA 2810454G14 GENE |
| 58238 | A830059I20Rik | RIKEN CDNA A830059I20 GENE |
| 106585 | Ankrd12 | ANKYRIN REPEAT DOMAIN 12-LIKE |
| 68144 | 5031426D15Rik | RIKEN CDNA 5031426D15 GENE |
| 67246 | 2810474O19Rik | RIKEN CDNA 2810474O19 GENE |
| 382985 | Rrm2b | RIBONUCLEOTIDE REDUCTASE M2 B (TP53 INDUCIBLE) |
| 114716 | Spred2 | SPROUTY-RELATED, EVH1 DOMAIN CONTAINING 2 |
| 20823 | Ssb | SJOGREN SYNDROME ANTIGEN B |
| 16998 | Ltbp3 | LATENT TRANSFORMING GROWTH FACTOR BETA BINDING PROTEIN 3 |
| 192167 | Nlgn1 | RIKEN CDNA 6330415N05 GENE |
| 98660 | Atp1a2 | ATPASE, NA+/K+ TRANSPORTING, ALPHA 2 POLYPEPTIDE |
| 319340 | C130065N10Rik | RIKEN CDNA C130065N10 GENE |
| 70432 | Rufy2 | DIFFERENTIALLY EXPRESSED IN NORMAL AND NEOPLASTIC CELLS |
| 14580 | Gfap | GLIAL FIBRILLARY ACIDIC PROTEIN |
| 108079 | Prkaa2 | PROTEIN KINASE, AMP-ACTIVATED, ALPHA 2 CATALYTIC SUBUNIT |
| 16573 | Kif5b | KINESIN FAMILY MEMBER 5B |
| 59057 | Zfp191 | ZINC FINGER PROTEIN 191 |
| 50490 | Nox4 | NADPH OXIDASE 4 |
| 235461 | B230380D07Rik | RIKEN CDNA B230380D07 GENE |
| 21838 | Thy1 | THYMUS CELL ANTIGEN 1, THETA |
| 11487 | Adam10 | A DISINTEGRIN AND METALLOPEPTIDASE DOMAIN 10 |
| 214663 | Slc25a29 | SOLUTE CARRIER FAMILY 25 |
| 70579 | Zc3h11a | ZINC FINGER CCCH TYPE CONTAINING 11A |
| 56376 | Pdlim5 | PDZ AND LIM DOMAIN 5 |
| 68465 | Adipor2 | EST AI115388 |
| 23965 | Odz3 | ODD OZ/TEN-M HOMOLOG 3 (DROSOPHILA) |
| 230257 | Rod1 | ROD1 REGULATOR OF DIFFERENTIATION 1 (S. POMBE) |

Table S6 Commonly regulated genes through dnADAM10 overexpression in mono- and double transgenic mice (dnADAM10 versus FVB/N (143 genes) compared to dnADAM10/APP_[V717I]_ versus APP_[V717I]_ (600 genes))

| **Table S6**  **Gene ID** | **Gene Symbol** | **Gene description** |
| --- | --- | --- |
| 66930 | Fank1 | FIBRONECTIN TYPE 3 AND ANKYRIN REPEAT DOMAINS 1 |
| 16017 | Igh-4 | IMMUNOGLOBULIN HEAVY CHAIN 4 (SERUM IGG1) |
| 20538 | Slc6a2 | SOLUTE CARRIER FAMILY 6 |
| 21838 | Thy1 | THYMUS CELL ANTIGEN 1, THETA |
| 11487 | Adam10 | A DISINTEGRIN AND METALLOPEPTIDASE DOMAIN 10 |
| 17444 | Grap2 | GRB2-RELATED ADAPTOR PROTEIN 2 |
| 99296 | Hrh3 | HISTAMINE RECEPTOR H 3 |
| 12772 | Ccr2 | CHEMOKINE (C-C MOTIF) RECEPTOR 2 |

**Table S7** 934 Alzheimer disease genes by GeneCards
(Weizmann Institute of Science, Version 2.36)

| **Table 7**  **Gene symbol** | **Description** |
| --- | --- |
| A2M | alpha-2-macroglobulin |
| AATF | apoptosis antagonizing transcription factor |
| ABAT | 4-aminobutyrate aminotransferase |
| ABCA1 | ATP-binding cassette, sub-family A (ABC1), member 1 |
| ABCA2 | ATP-binding cassette, sub-family A (ABC1), member 2 |
| ABCB1 | ATP-binding cassette, sub-family B (MDR/TAP), member 1 |
| ABI1 | abl-interactor 1 |
| ABL1 | v-abl Abelson murine leukemia viral oncogene homolog 1 |
| ABT1 | activator of basal transcription 1 |
| ACE | angiotensin I converting enzyme (peptidyl-dipeptidase A) 1 |
| ACHE | acetylcholinesterase (Yt blood group) |
| ACO2 | aconitase 2, mitochondrial |
| ACOT2 | acyl-CoA thioesterase 2 |
| ACOT4 | acyl-CoA thioesterase 4 |
| ACTA1 | actin, alpha 1, skeletal muscle |
| ACTA2 | actin, alpha 2, smooth muscle, aorta |
| ACTB | actin, beta |
| ACTC1 | actin, alpha, cardiac muscle 1 |
| ACTG1 | actin, gamma 1 |
| ACTG2 | actin, gamma 2, smooth muscle, enteric |
| AD10 | Alzheimer disease-10 |
| AD2 | Alzheimer disease 2 (APOE*E4-associated, late onset) |
| AD5 | Alzheimer disease 5 |
| AD6 | Alzheimer disease 6 |
| AD7 | Alzheimer disease 7 |
| AD7C-NTP | neuronal thread protein AD7c-NTP |
| AD8 | Alzheimer disease 8 |
| AD9 | Alzheimer disease 9 |
| ADAM10 | ADAM metallopeptidase domain 10 |
| ADAM12 | ADAM metallopeptidase domain 12 (meltrin alpha) |
| ADAM15 | ADAM metallopeptidase domain 15 (metargidin) |
| ADAM19 | ADAM metallopeptidase domain 19 (meltrin beta) |
| ADAMTS1 | ADAM metallopeptidase with thrombospondin type 1 motif, 1 |
| ADAMTS4 | ADAM metallopeptidase with thrombospondin type 1 motif, 4 |
| ADAMTS5 | ADAM metallopeptidase with thrombospondin type 1 motif, 5 (aggrecanase-2) |
| ADAMTSL1 | ADAMTS-like 1 |
| ADC | arginine decarboxylase |
| ADCY1 | adenylate cyclase 1 (brain) |
| ADCYAP1R1 | adenylate cyclase activating polypeptide 1 (pituitary) receptor type I |
| ADORA1 | adenosine A1 receptor |
| ADRA2C | adrenergic, alpha-2C-, receptor |
| ADRB1 | adrenergic, beta-1-, receptor |
| ADRBK1 | adrenergic, beta, receptor kinase 1 |
| AGER | advanced glycosylation end product-specific receptor |
| AGPAT1 | 1-acylglycerol-3-phosphate O-acyltransferase 1 (lysophosphatidic acid acyltransferase, alpha) |
| AGPS | alkylglycerone phosphate synthase |
| AGRIN | agrin |
| AGT | angiotensinogen (serpin peptidase inhibitor, clade A, member 8) |
| AHSG | alpha-2-HS-glycoprotein |
| AIFM1 | apoptosis-inducing factor, mitochondrion-associated, 1 |
| AKAP5 | A kinase (PRKA) anchor protein 5 |
| AKR1A1 | aldo-keto reductase family 1, member A1 (aldehyde reductase) |
| AKR1B1 | aldo-keto reductase family 1, member B1 (aldose reductase) |
| AKT1 | v-akt murine thymoma viral oncogene homolog 1 |
| ALDOA | aldolase A, fructose-bisphosphate |
| ALOX12 | arachidonate 12-lipoxygenase |
| ALOX15 | arachidonate 15-lipoxygenase |
| ALOX5AP | arachidonate 5-lipoxygenase-activating protein |
| AMD1 | adenosylmethionine decarboxylase 1 |
| AMPD1 | adenosine monophosphate deaminase 1 (isoform M) |
| AMPD3 | adenosine monophosphate deaminase (isoform E) |
| ANP32A | acidic (leucine-rich) nuclear phosphoprotein 32 family, member A |
| AOC3 | amine oxidase, copper containing 3 (vascular adhesion protein 1) |
| AP2B1 | adaptor-related protein complex 2, beta 1 subunit |
| APBA1 | amyloid beta (A4) precursor protein-binding, family A, member 1 (X11) |
| APBA2 | amyloid beta (A4) precursor protein-binding, family A, member 2 (X11-like) |
| APBA2BP | amyloid beta (A4) precursor protein-binding, family A, member 2 binding protein |
| APBA3 | amyloid beta (A4) precursor protein-binding, family A, member 3 (X11-like 2) |
| APBB1 | amyloid beta (A4) precursor protein-binding, family B, member 1 (Fe65) |
| APBB2 | amyloid beta (A4) precursor protein-binding, family B, member 2 (Fe65-like) |
| APBB3 | amyloid beta (A4) precursor protein-binding, family B, member 3 |
| APCS | amyloid P component, serum |
| APEX1 | APEX nuclease (multifunctional DNA repair enzyme) 1 |
| APH1A | anterior pharynx defective 1 homolog A (C. elegans) |
| APLP1 | amyloid beta (A4) precursor-like protein 1 |
| APLP2 | amyloid beta (A4) precursor-like protein 2 |
| APOA1 | apolipoprotein A-I |
| APOC1 | apolipoprotein C-I |
| APOC2 | apolipoprotein C-II |
| APOC3 | apolipoprotein C-III |
| APOD | apolipoprotein D |
| APOE | apolipoprotein E |
| APOF | apolipoprotein F |
| APOL1 | apolipoprotein L, 1 |
| APOM | apolipoprotein M |
| APP | amyloid beta (A4) precursor protein (peptidase nexin-II, Alzheimer disease) |
| APPBP1 | amyloid beta precursor protein binding protein 1 |
| 37347 | apoptosis related protein |
| ARPP-19 | cyclic AMP phosphoprotein, 19 kD |
| ARSA | arylsulfatase A |
| ASAH1 | N-acylsphingosine amidohydrolase (acid ceramidase) 1 |
| ASAH2 | N-acylsphingosine amidohydrolase (non-lysosomal ceramidase) 2 |
| ASAHL | N-acylsphingosine amidohydrolase (acid ceramidase)-like |
| ASS1 | argininosuccinate synthetase 1 |
| ATF2 | activating transcription factor 2 |
| ATG5 | ATG5 autophagy related 5 homolog (S. cerevisiae) |
| ATP12A | ATPase, H+/K+ transporting, nongastric, alpha polypeptide |
| ATP1A1 | ATPase, Na+/K+ transporting, alpha 1 polypeptide |
| ATP5J2 | ATP synthase, H+ transporting, mitochondrial F0 complex, subunit F2 |
| ATP7A | ATPase, Cu++ transporting, alpha polypeptide (Menkes syndrome) |
| AVEN | apoptosis, caspase activation inhibitor |
| AZU1 | azurocidin 1 (cationic antimicrobial protein 37) |
| BACE1 | beta-site APP-cleaving enzyme 1 |
| BACE2 | beta-site APP-cleaving enzyme 2 |
| BACH1 | BTB and CNC homology 1, basic leucine zipper transcription factor 1 |
| BAD | BCL2-antagonist of cell death |
| BAP1 | BRCA1 associated protein-1 (ubiquitin carboxy-terminal hydrolase) |
| BAX | BCL2-associated X protein |
| BCHE | butyrylcholinesterase |
| BCL2 | B-cell CLL/lymphoma 2 |
| BCL2A1 | BCL2-related protein A1 |
| BCL2L1 | BCL2-like 1 |
| BCL2L2 | BCL2-like 2 |
| BDKRB2 | bradykinin receptor B2 |
| BDNF | brain-derived neurotrophic factor |
| BGN | biglycan |
| BICC1 | bicaudal C homolog 1 (Drosophila) |
| BLMH | bleomycin hydrolase |
| BLVRB | biliverdin reductase B (flavin reductase (NADPH)) |
| BPTF | bromodomain PHD finger transcription factor |
| BSG | basigin (Ok blood group) |
| C1R | complement component 1, r subcomponent |
| C1S | complement component 1, s subcomponent |
| C20orf23 | chromosome 20 open reading frame 23 |
| C21orf33 | chromosome 21 open reading frame 33 |
| C3 | complement component 3 |
| C4BPA | complement component 4 binding protein, alpha |
| C5AR1 | complement component 5a receptor 1 |
| C6orf25 | chromosome 6 open reading frame 25 |
| C9 | complement component 9 |
| CA1 | carbonic anhydrase I |
| CA2 | carbonic anhydrase II |
| CALB1 | calbindin 1, 28kDa |
| CALB2 | calbindin 2, 29kDa (calretinin) |
| CAMK2A | calcium/calmodulin-dependent protein kinase (CaM kinase) II alpha |
| CAMK2G | calcium/calmodulin-dependent protein kinase (CaM kinase) II gamma |
| CAPN1 | calpain 1, (mu/I) large subunit |
| CAPN10 | calpain 10 |
| CAPN2 | calpain 2, (m/II) large subunit |
| CAPNS1 | calpain, small subunit 1 |
| CASP1 | caspase 1, apoptosis-related cysteine peptidase (interleukin 1, beta, convertase) |
| CASP14 | caspase 14, apoptosis-related cysteine peptidase |
| CASP2 | caspase 2, apoptosis-related cysteine peptidase (neural precursor cell expressed, developmentally down-regulated 2) |
| CASP3 | caspase 3, apoptosis-related cysteine peptidase |
| CASP4 | caspase 4, apoptosis-related cysteine peptidase |
| CASP6 | caspase 6, apoptosis-related cysteine peptidase |
| CASP7 | caspase 7, apoptosis-related cysteine peptidase |
| CASP8 | caspase 8, apoptosis-related cysteine peptidase |
| CASP9 | caspase 9, apoptosis-related cysteine peptidase |
| CAST | calpastatin |
| CAT | catalase |
| CAV1 | caveolin 1, caveolae protein, 22kDa |
| CAV2 | caveolin 2 |
| CAV3 | caveolin 3 |
| CBR1 | carbonyl reductase 1 |
| CBS | cystathionine-beta-synthase |
| CCBL1 | cysteine conjugate-beta lyase; cytoplasmic (glutamine transaminase K, kyneurenine aminotransferase) |
| CCL2 | chemokine (C-C motif) ligand 2 |
| CCL4 | chemokine (C-C motif) ligand 4 |
| CCL5 | chemokine (C-C motif) ligand 5 |
| CCNC | cyclin C |
| CCNG1 | cyclin G1 |
| CCR1 | chemokine (C-C motif) receptor 1 |
| CCR2 | chemokine (C-C motif) receptor 2 |
| CCR5 | chemokine (C-C motif) receptor 5 |
| CCT5 | chaperonin containing TCP1, subunit 5 (epsilon) |
| CD14 | CD14 molecule |
| CD200 | CD200 molecule |
| CD36 | CD36 molecule (thrombospondin receptor) |
| CD40 | CD40 molecule, TNF receptor superfamily member 5 |
| CD59 | CD59 molecule, complement regulatory protein |
| CD68 | CD68 molecule |
| CD93 | CD93 molecule |
| CD99 | CD99 molecule |
| CDC2 | cell division cycle 2, G1 to S and G2 to M |
| CDC25A | cell division cycle 25 homolog A (S. pombe) |
| CDK4 | cyclin-dependent kinase 4 |
| CDK5 | cyclin-dependent kinase 5 |
| CDK5R1 | cyclin-dependent kinase 5, regulatory subunit 1 (p35) |
| CDK5R2 | cyclin-dependent kinase 5, regulatory subunit 2 (p39) |
| CDKL1 | cyclin-dependent kinase-like 1 (CDC2-related kinase) |
| CDKN1B | cyclin-dependent kinase inhibitor 1B (p27, Kip1) |
| CDR1 | cerebellar degeneration-related protein 1, 34kDa |
| CEBPD | CCAAT/enhancer binding protein (C/EBP), delta |
| CENTA1 | centaurin, alpha 1 |
| CES1 | carboxylesterase 1 (monocyte/macrophage serine esterase 1) |
| CETP | cholesteryl ester transfer protein, plasma |
| CFDP1 | craniofacial development protein 1 |
| CFH | complement factor H |
| CFLAR | CASP8 and FADD-like apoptosis regulator |
| CFTR | cystic fibrosis transmembrane conductance regulator (ATP-binding cassette sub-family C, member 7) |
| CGI-38 | brain specific protein |
| CH25H | cholesterol 25-hydroxylase |
| CHAT | choline acetyltransferase |
| CHGB | chromogranin B (secretogranin 1) |
| CHIT1 | chitinase 1 (chitotriosidase) |
| CHKB | choline kinase beta |
| CHRM1 | cholinergic receptor, muscarinic 1 |
| CHRM2 | cholinergic receptor, muscarinic 2 |
| CHRM4 | cholinergic receptor, muscarinic 4 |
| CHRNA3 | cholinergic receptor, nicotinic, alpha 3 |
| CHRNA4 | cholinergic receptor, nicotinic, alpha 4 |
| CHRNA7 | cholinergic receptor, nicotinic, alpha 7 |
| CHRNB2 | cholinergic receptor, nicotinic, beta 2 (neuronal) |
| CIB1 | calcium and integrin binding 1 (calmyrin) |
| CKAP4 | cytoskeleton-associated protein 4 |
| CKB | creatine kinase, brain |
| CLCA2 | chloride channel, calcium activated, family member 2 |
| CLEC4A | C-type lectin domain family 4, member A |
| CLU | clusterin |
| CMPK | cytidylate kinase |
| CNP | 2',3'-cyclic nucleotide 3' phosphodiesterase |
| CNR1 | cannabinoid receptor 1 (brain) |
| CNR2 | cannabinoid receptor 2 (macrophage) |
| CNTF | ciliary neurotrophic factor |
| COL18A1 | collagen, type XVIII, alpha 1 |
| COL25A1 | collagen, type XXV, alpha 1 |
| COL4A1 | collagen, type IV, alpha 1 |
| COL4A2 | collagen, type IV, alpha 2 |
| COL4A3 | collagen, type IV, alpha 3 (Goodpasture antigen) |
| COL4A4 | collagen, type IV, alpha 4 |
| COL4A5 | collagen, type IV, alpha 5 (Alport syndrome) |
| COL4A6 | collagen, type IV, alpha 6 |
| COMT | catechol-O-methyltransferase |
| COX10 | COX10 homolog, cytochrome c oxidase assembly protein, heme A: farnesyltransferase (yeast) |
| COX4I1 | cytochrome c oxidase subunit IV isoform 1 |
| COX5A | cytochrome c oxidase subunit Va |
| COX6C | cytochrome c oxidase subunit VIc |
| CP | ceruloplasmin (ferroxidase) |
| CP20 | Lymphocyte cytosolic protein, molecular weight 20kD |
| CPA6 | carboxypeptidase A6 |
| CRADD | CASP2 and RIPK1 domain containing adaptor with death domain |
| CRAT | carnitine acetyltransferase |
| CRH | corticotropin releasing hormone |
| CRHBP | corticotropin releasing hormone binding protein |
| CROT | carnitine O-octanoyltransferase |
| CRP | C-reactive protein, pentraxin-related |
| CRX | cone-rod homeobox |
| CRYAB | crystallin, alpha B |
| CSNK1A1 | casein kinase 1, alpha 1 |
| CSNK1A1L | casein kinase 1, alpha 1-like |
| CSNK1D | casein kinase 1, delta |
| CSNK2A1 | casein kinase 2, alpha 1 polypeptide |
| CST3 | cystatin C (amyloid angiopathy and cerebral hemorrhage) |
| CSTA | cystatin A (stefin A) |
| CTCF | CCCTC-binding factor (zinc finger protein) |
| CTGF | connective tissue growth factor |
| CTNNA3 | catenin (cadherin-associated protein), alpha 3 |
| CTNNB1 | catenin (cadherin-associated protein), beta 1, 88kDa |
| CTRL | chymotrypsin-like |
| CTSB | cathepsin B |
| CTSD | cathepsin D |
| CTSE | cathepsin E |
| CTSG | cathepsin G |
| CTSH | cathepsin H |
| CTSS | cathepsin S |
| CXCR3 | chemokine (C-X-C motif) receptor 3 |
| CYCS | cytochrome c, somatic |
| CYP17A1 | cytochrome P450, family 17, subfamily A, polypeptide 1 |
| CYP19A1 | cytochrome P450, family 19, subfamily A, polypeptide 1 |
| CYP1A1 | cytochrome P450, family 1, subfamily A, polypeptide 1 |
| CYP27A1 | cytochrome P450, family 27, subfamily A, polypeptide 1 |
| CYP2D6 | cytochrome P450, family 2, subfamily D, polypeptide 6 |
| CYP46A1 | cytochrome P450, family 46, subfamily A, polypeptide 1 |
| CYP7B1 | cytochrome P450, family 7, subfamily B, polypeptide 1 |
| DAD-R | DAD1-related gene |
| DAPK1 | death-associated protein kinase 1 |
| DBN1 | drebrin 1 |
| DCX | doublecortex; lissencephaly, X-linked (doublecortin) |
| DFFA | DNA fragmentation factor, 45kDa, alpha polypeptide |
| DHCR24 | 24-dehydrocholesterol reductase |
| DHRS2 | dehydrogenase/reductase (SDR family) member 2 |
| DKK1 | dickkopf homolog 1 (Xenopus laevis) |
| DLD | dihydrolipoamide dehydrogenase |
| DLG1 | discs, large homolog 1 (Drosophila) |
| DLST | dihydrolipoamide S-succinyltransferase (E2 component of 2-oxo-glutarate complex) |
| DNTT | deoxynucleotidyltransferase, terminal |
| DOCK3 | dedicator of cytokinesis 3 |
| DPP6 | dipeptidyl-peptidase 6 |
| DPP7 | dipeptidyl-peptidase 7 |
| DPYSL2 | dihydropyrimidinase-like 2 |
| DRD1 | dopamine receptor D1 |
| DRD2 | dopamine receptor D2 |
| DRD3 | dopamine receptor D3 |
| DSCR1 | Down syndrome critical region gene 1 |
| DSTN | destrin (actin depolymerizing factor) |
| DUSP19 | dual specificity phosphatase 19 |
| DUSP5 | dual specificity phosphatase 5 |
| DVL1 | dishevelled, dsh homolog 1 (Drosophila) |
| DYRK1A | dual-specificity tyrosine-(Y)-phosphorylation regulated kinase 1A |
| DYSF | dysferlin, limb girdle muscular dystrophy 2B (autosomal recessive) |
| EBNA1BP2 | EBNA1 binding protein 2 |
| ECE1 | endothelin converting enzyme 1 |
| ECE2 | endothelin converting enzyme 2 |
| EEF2 | eukaryotic translation elongation factor 2 |
| EEF2K | eukaryotic elongation factor-2 kinase |
| EFCBP1 | EF-hand calcium binding protein 1 |
| EIF2AK2 | eukaryotic translation initiation factor 2-alpha kinase 2 |
| EIF2AK3 | eukaryotic translation initiation factor 2-alpha kinase 3 |
| EIF2B2 | eukaryotic translation initiation factor 2B, subunit 2 beta, 39kDa |
| EIF2S1 | eukaryotic translation initiation factor 2, subunit 1 alpha, 35kDa |
| EIF4E | eukaryotic translation initiation factor 4E |
| EIF4EBP1 | eukaryotic translation initiation factor 4E binding protein 1 |
| EIF4G2 | eukaryotic translation initiation factor 4 gamma, 2 |
| EMD | emerin (Emery-Dreifuss muscular dystrophy) |
| ENO1 | enolase 1, (alpha) |
| ENPP2 | ectonucleotide pyrophosphatase/phosphodiesterase 2 (autotaxin) |
| ENTPD5 | ectonucleoside triphosphate diphosphohydrolase 5 |
| EPHX1 | epoxide hydrolase 1, microsomal (xenobiotic) |
| ERBB4 | v-erb-a erythroblastic leukemia viral oncogene homolog 4 (avian) |
| ERCC2 | excision repair cross-complementing rodent repair deficiency, complementation group 2 (xeroderma pigmentosum D) |
| ERCC4 | excision repair cross-complementing rodent repair deficiency, complementation group 4 |
| ERG | v-ets erythroblastosis virus E26 oncogene homolog (avian) |
| ERN1 | endoplasmic reticulum to nucleus signalling 1 |
| ESR1 | estrogen receptor 1 |
| ESR2 | estrogen receptor 2 (ER beta) |
| F12 | coagulation factor XII (Hageman factor) |
| F13A1 | coagulation factor XIII, A1 polypeptide |
| F2 | coagulation factor II (thrombin) |
| FAAH | fatty acid amide hydrolase |
| FABP3 | fatty acid binding protein 3, muscle and heart (mammary-derived growth inhibitor) |
| FABP5 | fatty acid binding protein 5 (psoriasis-associated) |
| FABP7 | fatty acid binding protein 7, brain |
| FANCD2 | Fanconi anemia, complementation group D2 |
| FAS | Fas (TNF receptor superfamily, member 6) |
| FASLG | Fas ligand (TNF superfamily, member 6) |
| FBP2 | fructose-1,6-bisphosphatase 2 |
| FGF1 | fibroblast growth factor 1 (acidic) |
| FGFR1 | fibroblast growth factor receptor 1 (fms-related tyrosine kinase 2, Pfeiffer syndrome) |
| FHL2 | four and a half LIM domains 2 |
| FIBP | fibroblast growth factor (acidic) intracellular binding protein |
| FLNB | filamin B, beta (actin binding protein 278) |
| FLOT1 | flotillin 1 |
| FOLH1 | folate hydrolase (prostate-specific membrane antigen) 1 |
| FOS | v-fos FBJ murine osteosarcoma viral oncogene homolog |
| FPRL1 | formyl peptide receptor-like 1 |
| FRAP1 | FK506 binding protein 12-rapamycin associated protein 1 |
| FRK | fyn-related kinase |
| FTH1 | ferritin, heavy polypeptide 1 |
| FTL | ferritin, light polypeptide |
| FTMT | ferritin mitochondrial |
| FURIN | furin (paired basic amino acid cleaving enzyme) |
| FYN | FYN oncogene related to SRC, FGR, YES |
| GAB2 | GRB2-associated binding protein 2 |
| GADD45A | growth arrest and DNA-damage-inducible, alpha |
| GAL | galanin |
| GALR2 | galanin receptor 2 |
| GALR3 | galanin receptor 3 |
| GAP43 | growth associated protein 43 |
| GAPDH | glyceraldehyde-3-phosphate dehydrogenase |
| GART | phosphoribosylglycinamide formyltransferase, phosphoribosylglycinamide synthetase, phosphoribosylaminoimidazole synthetase |
| GBL | G protein beta subunit-like |
| GDI1 | GDP dissociation inhibitor 1 |
| GER | Gastroesophageal reflux |
| GFAP | glial fibrillary acidic protein |
| GGT2 | gamma-glutamyltransferase 2 |
| GLO1 | glyoxalase I |
| GLP1R | glucagon-like peptide 1 receptor |
| GLS | glutaminase |
| GLUL | glutamate-ammonia ligase (glutamine synthetase) |
| GNAS | GNAS complex locus |
| GNB2L1 | guanine nucleotide binding protein (G protein), beta polypeptide 2-like 1 |
| GNB3 | guanine nucleotide binding protein (G protein), beta polypeptide 3 |
| GPC1 | glypican 1 |
| GPHA2 | glycoprotein hormone alpha 2 |
| GPX3 | glutathione peroxidase 3 (plasma) |
| GRB2 | growth factor receptor-bound protein 2 |
| GRIA1 | glutamate receptor, ionotropic, AMPA 1 |
| GRIA2 | glutamate receptor, ionotropic, AMPA 2 |
| GRIA3 | glutamate receptor, ionotrophic, AMPA 3 |
| GRIN1 | glutamate receptor, ionotropic, N-methyl D-aspartate 1 |
| GRIN2A | glutamate receptor, ionotropic, N-methyl D-aspartate 2A |
| GRIN2B | glutamate receptor, ionotropic, N-methyl D-aspartate 2B |
| GRM1 | glutamate receptor, metabotropic 1 |
| GRM2 | glutamate receptor, metabotropic 2 |
| GRM3 | glutamate receptor, metabotropic 3 |
| GSK3A | glycogen synthase kinase 3 alpha |
| GSK3B | glycogen synthase kinase 3 beta |
| GSS | glutathione synthetase |
| GSTM1 | glutathione S-transferase M1 |
| GSTM3 | glutathione S-transferase M3 (brain) |
| GSTO1 | glutathione S-transferase omega 1 |
| GSTO2 | glutathione S-transferase omega 2 |
| GSTT1 | glutathione S-transferase theta 1 |
| HADHA | hydroxyacyl-Coenzyme A dehydrogenase/3-ketoacyl-Coenzyme A thiolase/enoyl-Coenzyme A hydratase (trifunctional protein), alpha subunit |
| HADHB | hydroxyacyl-Coenzyme A dehydrogenase/3-ketoacyl-Coenzyme A thiolase/enoyl-Coenzyme A hydratase (trifunctional protein), beta subunit |
| HAGH | hydroxyacylglutathione hydrolase |
| HAPP | huntingtin-associated protein 1, pseudogene |
| HCCA2 | HCCA2 protein |
| HCLS1 | hematopoietic cell-specific Lyn substrate 1 |
| HERPUD1 | homocysteine-inducible, endoplasmic reticulum stress-inducible, ubiquitin-like domain member 1 |
| HFE | hemochromatosis |
| HHEX | homeobox, hematopoietically expressed |
| HIP2 | huntingtin interacting protein 2 |
| HIST1H1B | histone cluster 1, H1b |
| HIST4H4 | histone cluster 4, H4 |
| HLA-16 | HLA-16 pseudogene |
| HLA-21 | HLA-21 pseudogene |
| HLA-75 | HLA-75 pseudogene |
| HLA-80 | HLA-80 pseudogene |
| HLA-90 | HLA-90 pseudogene |
| HLA-A | major histocompatibility complex, class I, A |
| HLA-B | major histocompatibility complex, class I, B |
| HLA-C | major histocompatibility complex, class I, C |
| HLA-DMA | major histocompatibility complex, class II, DM alpha |
| HLA-DMB | major histocompatibility complex, class II, DM beta |
| HLA-DOA | major histocompatibility complex, class II, DO alpha |
| HLA-DOB | major histocompatibility complex, class II, DO beta |
| HLA-DPA1 | major histocompatibility complex, class II, DP alpha 1 |
| HLA-DPA2 | major histocompatibility complex, class II, DP alpha 2 (pseudogene) |
| HLA-DPA3 | major histocompatibility complex, class II, DP alpha 3 (pseudogene) |
| HLA-DPB1 | major histocompatibility complex, class II, DP beta 1 |
| HLA-DPB2 | major histocompatibility complex, class II, DP beta 2 (pseudogene) |
| HLA-DQA1 | major histocompatibility complex, class II, DQ alpha 1 |
| HLA-DQA2 | major histocompatibility complex, class II, DQ alpha 2 |
| HLA-DQB2 | major histocompatibility complex, class II, DQ beta 2 |
| HLA-DQB3 | major histocompatibility complex, class II, DQ beta 3 |
| HLA-DRA | major histocompatibility complex, class II, DR alpha |
| HLA-DRB1 | major histocompatibility complex, class II, DR beta 1 |
| HLA-DRB2 | major histocompatibility complex, class II, DR beta 2 |
| HLA-DRB3 | major histocompatibility complex, class II, DR beta 3 |
| HLA-DRB4 | major histocompatibility complex, class II, DR beta 4 |
| HLA-DRB5 | major histocompatibility complex, class II, DR beta 5 |
| HLA-DRB6 | major histocompatibility complex, class II, DR beta 6 (pseudogene) |
| HLA-DRB7 | major histocompatibility complex, class II, DR beta 7 (pseudogene) |
| HLA-DRB8 | major histocompatibility complex, class II, DR beta 8 (pseudogene) |
| HLA-DRB9 | major histocompatibility complex, class II, DR beta 9 (pseudogene) |
| HLA-E | major histocompatibility complex, class I, E |
| HLA-F | major histocompatibility complex, class I, F |
| HLA-G | HLA-G histocompatibility antigen, class I, G |
| HLA-H | major histocompatibility complex, class I, H (pseudogene) |
| HLA-J | major histocompatibility complex, class I, J (pseudogene) |
| HLA-K | major histocompatibility complex, class I, K |
| HLA-L | major histocompatibility complex, class I, L |
| HLA-N | major histocompatibility complex, class I, N (pseudogene) |
| HLA-S | major histocompatibility complex, class I, S (pseudogene) |
| HLA-X | major histocompatibility complex, class I, X (pseudogene) |
| HLA-Z | major histocompatibility complex, class I, Z (pseudogene) |
| HMGA1 | high mobility group AT-hook 1 |
| HMGCR | 3-hydroxy-3-methylglutaryl-Coenzyme A reductase |
| HMI | hypomelanosis of Ito |
| HMOX1 | heme oxygenase (decycling) 1 |
| HMOX2 | heme oxygenase (decycling) 2 |
| HNMT | histamine N-methyltransferase |
| HNRPA2B1 | heterogeneous nuclear ribonucleoprotein A2/B1 |
| HNRPDL | heterogeneous nuclear ribonucleoprotein D-like |
| HPCAL1 | hippocalcin-like 1 |
| HPX | hemopexin |
| HRAS | v-Ha-ras Harvey rat sarcoma viral oncogene homolog |
| HRH2 | histamine receptor H2 |
| HSD11B1 | hydroxysteroid (11-beta) dehydrogenase 1 |
| HSD11B1L | hydroxysteroid (11-beta) dehydrogenase 1-like |
| HSD17B10 | hydroxysteroid (17-beta) dehydrogenase 10 |
| HSF1 | heat shock transcription factor 1 |
| HSPA1B | heat shock 70kDa protein 1B |
| HSPA2 | heat shock 70kDa protein 2 |
| HSPA5 | heat shock 70kDa protein 5 (glucose-regulated protein, 78kDa) |
| HSPA9 | heat shock 70kDa protein 9 (mortalin) |
| HSPB2 | heat shock 27kDa protein 2 |
| HSPB6 | heat shock protein, alpha-crystallin-related, B6 |
| HSPB8 | heat shock 22kDa protein 8 |
| HSPG2 | heparan sulfate proteoglycan 2 (perlecan) |
| HTR1A | 5-hydroxytryptamine (serotonin) receptor 1A |
| HTR2A | 5-hydroxytryptamine (serotonin) receptor 2A |
| HTR2C | 5-hydroxytryptamine (serotonin) receptor 2C |
| HTR6 | 5-hydroxytryptamine (serotonin) receptor 6 |
| HTRA1 | HtrA serine peptidase 1 |
| HTRA2 | HtrA serine peptidase 2 |
| IAPP | islet amyloid polypeptide |
| ICAM1 | intercellular adhesion molecule 1 (CD54), human rhinovirus receptor |
| ICAM2 | intercellular adhesion molecule 2 |
| ICAM5 | intercellular adhesion molecule 5, telencephalin |
| ICMT | isoprenylcysteine carboxyl methyltransferase |
| IDE | insulin-degrading enzyme |
| IFNG | interferon, gamma |
| IGF1 | insulin-like growth factor 1 (somatomedin C) |
| IGF2 | insulin-like growth factor 2 (somatomedin A) |
| IGF2R | insulin-like growth factor 2 receptor |
| IGFBP3 | insulin-like growth factor binding protein 3 |
| IGHM | immunoglobulin heavy constant mu |
| IKZF1 | IKAROS family zinc finger 1 (Ikaros) |
| IL10 | interleukin 10 |
| IL1A | interleukin 1, alpha |
| IL1B | interleukin 1, beta |
| IL1R1 | interleukin 1 receptor, type I |
| IL1R2 | interleukin 1 receptor, type II |
| IL1RN | interleukin 1 receptor antagonist |
| IL28RA | interleukin 28 receptor, alpha (interferon, lambda receptor) |
| IL6 | interleukin 6 (interferon, beta 2) |
| IL8 | interleukin 8 |
| IMPA1 | inositol(myo)-1(or 4)-monophosphatase 1 |
| INA | internexin neuronal intermediate filament protein, alpha |
| INHA | inhibin, alpha |
| INHBA | inhibin, beta A (activin A, activin AB alpha polypeptide) |
| INHBB | inhibin, beta B (activin AB beta polypeptide) |
| INS | insulin |
| INSRL | insulin receptor-like |
| ISG20 | interferon stimulated exonuclease gene 20kDa |
| ITGAM | integrin, alpha M (complement component 3 receptor 3 subunit) |
| ITM2B | integral membrane protein 2B |
| ITPKB | inositol 1,4,5-trisphosphate 3-kinase B |
| ITPR3 | inositol 1,4,5-triphosphate receptor, type 3 |
| ITSN1 | intersectin 1 (SH3 domain protein) |
| K-ALPHA-1 | alpha tubulin |
| KCNA2 | potassium voltage-gated channel, shaker-related subfamily, member 2 |
| KCNA4 | potassium voltage-gated channel, shaker-related subfamily, member 4 |
| KCNC4 | potassium voltage-gated channel, Shaw-related subfamily, member 4 |
| KCNIP3 | Kv channel interacting protein 3, calsenilin |
| KCNN1 | potassium intermediate/small conductance calcium-activated channel, subfamily N, member 1 |
| KCNN4 | potassium intermediate/small conductance calcium-activated channel, subfamily N, member 4 |
| KHSRP | KH-type splicing regulatory protein (FUSE binding protein 2) |
| KIF11 | kinesin family member 11 |
| KIFAP3 | kinesin-associated protein 3 |
| KLK10 | kallikrein-related peptidase 10 |
| KLK11 | kallikrein-related peptidase 11 |
| KLK15 | kallikrein-related peptidase 15 |
| KLK6 | kallikrein-related peptidase 6 |
| KLK7 | kallikrein-related peptidase 7 |
| KLK8 | kallikrein-related peptidase 8 |
| LAMA1 | laminin, alpha 1 |
| LAMC1 | laminin, gamma 1 (formerly LAMB2) |
| LAMP1 | lysosomal-associated membrane protein 1 |
| LDLR | low density lipoprotein receptor (familial hypercholesterolemia) |
| LHCGR | luteinizing hormone/choriogonadotropin receptor |
| LIMK1 | LIM domain kinase 1 |
| LIPA | lipase A, lysosomal acid, cholesterol esterase (Wolman disease) |
| LMAN1 | lectin, mannose-binding, 1 |
| LOC642043 | similar to HLA class II histocompatibility antigen, DP alpha chain precursor (HLA-SB alpha chain) (MHC class II DP3-alpha) (DP(W3)) (DP(W4)) |
| LOC644264 | similar to fetal Alzheimer antigen isoform 2 |
| LOC652907 | similar to Lysosome-associated membrane glycoprotein 1 precursor (LAMP-1) (CD107a antigen) |
| LONP1 | lon peptidase 1, mitochondrial |
| LPA | lipoprotein, Lp(a) |
| LPL | lipoprotein lipase |
| LRP1 | low density lipoprotein-related protein 1 (alpha-2-macroglobulin receptor) |
| LRP8 | low density lipoprotein receptor-related protein 8, apolipoprotein e receptor |
| LRPAP1 | low density lipoprotein receptor-related protein associated protein 1 |
| LRRK2 | leucine-rich repeat kinase 2 |
| M6PR | mannose-6-phosphate receptor (cation dependent) |
| MADD | MAP-kinase activating death domain |
| MAOA | monoamine oxidase A |
| MAOB | monoamine oxidase B |
| MAP1B | microtubule-associated protein 1B |
| MAP2 | microtubule-associated protein 2 |
| MAP2K1 | mitogen-activated protein kinase kinase 1 |
| MAP2K2 | mitogen-activated protein kinase kinase 2 |
| MAP2K6 | mitogen-activated protein kinase kinase 6 |
| MAPK1 | mitogen-activated protein kinase 1 |
| MAPK14 | mitogen-activated protein kinase 14 |
| MAPK3 | mitogen-activated protein kinase 3 |
| MAPK8IP1 | mitogen-activated protein kinase 8 interacting protein 1 |
| MAPKAPK2 | mitogen-activated protein kinase-activated protein kinase 2 |
| MAPT | microtubule-associated protein tau |
| MARCKS | myristoylated alanine-rich protein kinase C substrate |
| MARK1 | MAP/microtubule affinity-regulating kinase 1 |
| MAT1A | methionine adenosyltransferase I, alpha |
| MAZ | MYC-associated zinc finger protein (purine-binding transcription factor) |
| MBL2 | mannose-binding lectin (protein C) 2, soluble (opsonic defect) |
| MCM2 | MCM2 minichromosome maintenance deficient 2, mitotin (S. cerevisiae) |
| MDH1 | malate dehydrogenase 1, NAD (soluble) |
| MEF2A | MADS box transcription enhancer factor 2, polypeptide A (myocyte enhancer factor 2A) |
| MEOX2 | mesenchyme homeobox 2 |
| MFI2 | antigen p97 (melanoma associated) identified by monoclonal antibodies 133.2 and 96.5 |
| MIF | macrophage migration inhibitory factor (glycosylation-inhibiting factor) |
| MIXL1 | Mix1 homeobox-like 1 (Xenopus laevis) |
| MLH3 | mutL homolog 3 (E. coli) |
| MME | membrane metallo-endopeptidase (neutral endopeptidase, enkephalinase) |
| MMP1 | matrix metallopeptidase 1 (interstitial collagenase) |
| MMP3 | matrix metallopeptidase 3 (stromelysin 1, progelatinase) |
| MMP9 | matrix metallopeptidase 9 (gelatinase B, 92kDa gelatinase, 92kDa type IV collagenase) |
| MPO | myeloperoxidase |
| MPP1 | membrane protein, palmitoylated 1, 55kDa |
| MRE11A | MRE11 meiotic recombination 11 homolog A (S. cerevisiae) |
| MS | multiple sclerosis |
| MSI1 | musashi homolog 1 (Drosophila) |
| MSR1 | macrophage scavenger receptor 1 |
| MSRA | methionine sulfoxide reductase A |
| MT-CO1 | mitochondrially encoded cytochrome c oxidase I |
| MT-ND1 | mitochondrially encoded NADH dehydrogenase 1 |
| MT-ND2 | mitochondrially encoded NADH dehydrogenase 2 |
| MT-ND4 | mitochondrially encoded NADH dehydrogenase 4 |
| MT-ND5 | mitochondrially encoded NADH dehydrogenase 5 |
| MT-RNR2 | mitochondrially encoded 16S RNA |
| MT-TG | mitochondrially encoded tRNA glycine |
| MT1A | metallothionein 1A (functional) |
| MT2A | metallothionein 2A |
| MT3 | metallothionein 3 |
| MTF2 | metal response element binding transcription factor 2 |
| MTHFD1 | methylenetetrahydrofolate dehydrogenase (NADP+ dependent) 1, methenyltetrahydrofolate cyclohydrolase, formyltetrahydrofolate synthetase |
| MTHFR | 5,10-methylenetetrahydrofolate reductase (NADPH) |
| MTNR1A | melatonin receptor 1A |
| MTR | 5-methyltetrahydrofolate-homocysteine methyltransferase |
| MTRR | 5-methyltetrahydrofolate-homocysteine methyltransferase reductase |
| MX1 | myxovirus (influenza virus) resistance 1, interferon-inducible protein p78 (mouse) |
| MYOCD | myocardin |
| NACA | nascent-polypeptide-associated complex alpha polypeptide |
| NAPG | N-ethylmaleimide-sensitive factor attachment protein, gamma |
| NAPSA | napsin A aspartic peptidase |
| NAPSB | napsin B aspartic peptidase pseudogene |
| NAT1 | N-acetyltransferase 1 (arylamine N-acetyltransferase) |
| NCAM1 | neural cell adhesion molecule 1 |
| NCK1 | NCK adaptor protein 1 |
| NCKAP1 | NCK-associated protein 1 |
| NCKIPSD | NCK interacting protein with SH3 domain |
| NCL | nucleolin |
| NCSTN | nicastrin |
| NDRG2 | NDRG family member 2 |
| NEDD8 | neural precursor cell expressed, developmentally down-regulated 8 |
| NEDD9 | neural precursor cell expressed, developmentally down-regulated 9 |
| NEFH | neurofilament, heavy polypeptide 200kDa |
| NEFL | neurofilament, light polypeptide 68kDa |
| NEFM | neurofilament, medium polypeptide 150kDa |
| NEWENTRY | Record to support submission of GeneRIFs for a gene not in Entrez Gene (human; man). |
| NGFB | nerve growth factor, beta polypeptide |
| NGFR | nerve growth factor receptor (TNFR superfamily, member 16) |
| NID1 | nidogen 1 |
| NISCH | nischarin |
| NME1 | non-metastatic cells 1, protein (NM23A) expressed in |
| NME2 | non-metastatic cells 2, protein (NM23B) expressed in |
| NMUR1 | neuromedin U receptor 1 |
| NOS1 | nitric oxide synthase 1 (neuronal) |
| NOS2A | nitric oxide synthase 2A (inducible, hepatocytes) |
| NOS3 | nitric oxide synthase 3 (endothelial cell) |
| NOTCH1 | Notch homolog 1, translocation-associated (Drosophila) |
| NOTCH2 | Notch homolog 2 (Drosophila) |
| NOTCH4 | Notch homolog 4 (Drosophila) |
| NOX1 | NADPH oxidase 1 |
| NPC1 | Niemann-Pick disease, type C1 |
| NPFFR2 | neuropeptide FF receptor 2 |
| NPTX1 | neuronal pentraxin I |
| NPY | neuropeptide Y |
| NQO1 | NAD(P)H dehydrogenase, quinone 1 |
| NR1H3 | nuclear receptor subfamily 1, group H, member 3 |
| NR4A1 | nuclear receptor subfamily 4, group A, member 1 |
| NRG1 | neuregulin 1 |
| NRGN | neurogranin (protein kinase C substrate, RC3) |
| NRIP1 | nuclear receptor interacting protein 1 |
| NRM | nurim (nuclear envelope membrane protein) |
| NT5C | 5', 3'-nucleotidase, cytosolic |
| NTF3 | neurotrophin 3 |
| NTRK1 | neurotrophic tyrosine kinase, receptor, type 1 |
| NTRK2 | neurotrophic tyrosine kinase, receptor, type 2 |
| NTRK3 | neurotrophic tyrosine kinase, receptor, type 3 |
| NUMB | numb homolog (Drosophila) |
| NUTF2 | nuclear transport factor 2 |
| OGDH | oxoglutarate (alpha-ketoglutarate) dehydrogenase (lipoamide) |
| OGG1 | 8-oxoguanine DNA glycosylase |
| OLFR@ | olfactory receptor cluster |
| OLR1 | oxidized low density lipoprotein (lectin-like) receptor 1 |
| OPRK1 | opioid receptor, kappa 1 |
| OPRL1 | opiate receptor-like 1 |
| P18SRP | P18SRP protein |
| P2RY1 | purinergic receptor P2Y, G-protein coupled, 1 |
| P4HB | procollagen-proline, 2-oxoglutarate 4-dioxygenase (proline 4-hydroxylase), beta polypeptide |
| PADI2 | peptidyl arginine deiminase, type II |
| PAK3 | p21 (CDKN1A)-activated kinase 3 |
| PARK2 | Parkinson disease (autosomal recessive, juvenile) 2, parkin |
| PARK7 | Parkinson disease (autosomal recessive, early onset) 7 |
| PAWR | PRKC, apoptosis, WT1, regulator |
| PCMT1 | protein-L-isoaspartate (D-aspartate) O-methyltransferase |
| PCSK1 | proprotein convertase subtilisin/kexin type 1 |
| PCSK1N | proprotein convertase subtilisin/kexin type 1 inhibitor |
| PCSK2 | proprotein convertase subtilisin/kexin type 2 |
| PCSK7 | proprotein convertase subtilisin/kexin type 7 |
| PCSK9 | proprotein convertase subtilisin/kexin type 9 |
| PCTK3 | PCTAIRE protein kinase 3 |
| PCYOX1 | prenylcysteine oxidase 1 |
| PDCD6 | programmed cell death 6 |
| PDE7A | phosphodiesterase 7A |
| PDE7B | phosphodiesterase 7B |
| PDE8A | phosphodiesterase 8A |
| PDE8B | phosphodiesterase 8B |
| PDGFA | platelet-derived growth factor alpha polypeptide |
| PEBP1 | phosphatidylethanolamine binding protein 1 |
| PEMT | phosphatidylethanolamine N-methyltransferase |
| PFKP | phosphofructokinase, platelet |
| PGAM1 | phosphoglycerate mutase 1 (brain) |
| PGCP | plasma glutamate carboxypeptidase |
| PHKG1 | phosphorylase kinase, gamma 1 (muscle) |
| PHYH | phytanoyl-CoA 2-hydroxylase |
| PIK3R1 | phosphoinositide-3-kinase, regulatory subunit 1 (p85 alpha) |
| PIN1 | protein (peptidylprolyl cis/trans isomerase) NIMA-interacting 1 |
| PIP5KL1 | phosphatidylinositol-4-phosphate 5-kinase-like 1 |
| PITRM1 | pitrilysin metallopeptidase 1 |
| PKM2 | pyruvate kinase, muscle |
| PKN1 | protein kinase N1 |
| PKP4 | plakophilin 4 |
| PLA2G4A | phospholipase A2, group IVA (cytosolic, calcium-dependent) |
| PLA2G6 | phospholipase A2, group VI (cytosolic, calcium-independent) |
| PLAU | plasminogen activator, urokinase |
| PLCB1 | phospholipase C, beta 1 (phosphoinositide-specific) |
| PLCD1 | phospholipase C, delta 1 |
| PLD1 | phospholipase D1, phosphatidylcholine-specific |
| PLG | plasminogen |
| PLTP | phospholipid transfer protein |
| PLXNA1 | plexin A1 |
| PNMT | phenylethanolamine N-methyltransferase |
| PON1 | paraoxonase 1 |
| PON2 | paraoxonase 2 |
| PON3 | paraoxonase 3 |
| POU2F1 | POU domain, class 2, transcription factor 1 |
| PPARA | peroxisome proliferator-activated receptor alpha |
| PPARBP | PPAR binding protein |
| PPARG | peroxisome proliferator-activated receptor gamma |
| PPBP | pro-platelet basic protein (chemokine (C-X-C motif) ligand 7) |
| PPM2C | protein phosphatase 2C, magnesium-dependent, catalytic subunit |
| PPP1R10 | protein phosphatase 1, regulatory (inhibitor) subunit 10 |
| PPP1R3A | protein phosphatase 1, regulatory (inhibitor) subunit 3A (glycogen and sarcoplasmic reticulum binding subunit, skeletal muscle) |
| PPP2CA | protein phosphatase 2 (formerly 2A), catalytic subunit, alpha isoform |
| PPP2CB | protein phosphatase 2 (formerly 2A), catalytic subunit, beta isoform |
| PPP2R2A | protein phosphatase 2 (formerly 2A), regulatory subunit B, alpha isoform |
| PPP3CA | protein phosphatase 3 (formerly 2B), catalytic subunit, alpha isoform |
| PPP5C | protein phosphatase 5, catalytic subunit |
| PPYR1 | pancreatic polypeptide receptor 1 |
| PRDX1 | peroxiredoxin 1 |
| PRDX2 | peroxiredoxin 2 |
| PRDX3 | peroxiredoxin 3 |
| PRDX5 | peroxiredoxin 5 |
| PRDX6 | peroxiredoxin 6 |
| PREP | prolyl endopeptidase |
| PRKACA | protein kinase, cAMP-dependent, catalytic, alpha |
| PRKAR2B | protein kinase, cAMP-dependent, regulatory, type II, beta |
| PRKCA | protein kinase C, alpha |
| PRKCD | protein kinase C, delta |
| PRKCG | protein kinase C, gamma |
| PRKCZ | protein kinase C, zeta |
| PRKG1 | protein kinase, cGMP-dependent, type I |
| PRND | prion protein 2 (dublet) |
| PRNP | prion protein (p27-30) (Creutzfeldt-Jakob disease, Gerstmann-Strausler-Scheinker syndrome, fatal familial insomnia) |
| PRSS1 | protease, serine, 1 (trypsin 1) |
| PRSS7 | protease, serine, 7 (enterokinase) |
| PSEN1 | presenilin 1 (Alzheimer disease 3) |
| PSEN2 | presenilin 2 (Alzheimer disease 4) |
| PSENEN | presenilin enhancer 2 homolog (C. elegans) |
| PSMB6 | proteasome (prosome, macropain) subunit, beta type, 6 |
| PSMB9 | proteasome (prosome, macropain) subunit, beta type, 9 (large multifunctional peptidase 2) |
| PSMC1 | proteasome (prosome, macropain) 26S subunit, ATPase, 1 |
| PSMC2 | proteasome (prosome, macropain) 26S subunit, ATPase, 2 |
| PSMC6 | proteasome (prosome, macropain) 26S subunit, ATPase, 6 |
| PSMD2 | proteasome (prosome, macropain) 26S subunit, non-ATPase, 2 |
| PTGER2 | prostaglandin E receptor 2 (subtype EP2), 53kDa |
| PTGS1 | prostaglandin-endoperoxide synthase 1 (prostaglandin G/H synthase and cyclooxygenase) |
| PTGS2 | prostaglandin-endoperoxide synthase 2 (prostaglandin G/H synthase and cyclooxygenase) |
| PTN | pleiotrophin (heparin binding growth factor 8, neurite growth-promoting factor 1) |
| PTPRA | protein tyrosine phosphatase, receptor type, A |
| PVALB | parvalbumin |
| RAB3A | RAB3A, member RAS oncogene family |
| RAB5A | RAB5A, member RAS oncogene family |
| RAB6A | RAB6A, member RAS oncogene family |
| RABEP2 | rabaptin, RAB GTPase binding effector protein 2 |
| RABGAP1L | RAB GTPase activating protein 1-like |
| RAF1 | v-raf-1 murine leukemia viral oncogene homolog 1 |
| RALA | v-ral simian leukemia viral oncogene homolog A (ras related) |
| RALB | v-ral simian leukemia viral oncogene homolog B (ras related; GTP binding protein) |
| RAPGEF3 | Rap guanine nucleotide exchange factor (GEF) 3 |
| RASA1 | RAS p21 protein activator (GTPase activating protein) 1 |
| RBM25 | RNA binding motif protein 25 |
| REG1A | regenerating islet-derived 1 alpha (pancreatic stone protein, pancreatic thread protein) |
| RELN | reelin |
| REN | renin |
| RGS4 | regulator of G-protein signalling 4 |
| RGS6 | regulator of G-protein signalling 6 |
| RHOA | ras homolog gene family, member A |
| RIPK2 | receptor-interacting serine-threonine kinase 2 |
| RNF146 | ring finger protein 146 |
| RPGR | retinitis pigmentosa GTPase regulator |
| RPS27A | ribosomal protein S27a |
| RPS3A | ribosomal protein S3A |
| RPS6KA3 | ribosomal protein S6 kinase, 90kDa, polypeptide 3 |
| RPS6KA5 | ribosomal protein S6 kinase, 90kDa, polypeptide 5 |
| RPS6KB1 | ribosomal protein S6 kinase, 70kDa, polypeptide 1 |
| RPS6KB2 | ribosomal protein S6 kinase, 70kDa, polypeptide 2 |
| RTKN | rhotekin |
| RTN4 | reticulon 4 |
| RTN4R | reticulon 4 receptor |
| RYR1 | ryanodine receptor 1 (skeletal) |
| S100A12 | S100 calcium binding protein A12 |
| S100A6 | S100 calcium binding protein A6 |
| S100A9 | S100 calcium binding protein A9 |
| S100B | S100 calcium binding protein B |
| S100G | S100 calcium binding protein G |
| SAA4 | serum amyloid A4, constitutive |
| SCARB1 | scavenger receptor class B, member 1 |
| SCG2 | secretogranin II (chromogranin C) |
| SCO1 | SCO cytochrome oxidase deficient homolog 1 (yeast) |
| SDC2 | syndecan 2 (heparan sulfate proteoglycan 1, cell surface-associated, fibroglycan) |
| SDHB | succinate dehydrogenase complex, subunit B, iron sulfur (Ip) |
| SEL1L | sel-1 suppressor of lin-12-like (C. elegans) |
| SELM | selenoprotein M |
| SEMA3A | sema domain, immunoglobulin domain (Ig), short basic domain, secreted, (semaphorin) 3A |
| 37135 | septin 1 |
| 37500 | septin 2 |
| 37865 | septin 3 |
| 38231 | septin 4 |
| SEPX1 | selenoprotein X, 1 |
| SERPINA3 | serpin peptidase inhibitor, clade A (alpha-1 antiproteinase, antitrypsin), member 3 |
| SERPINB2 | serpin peptidase inhibitor, clade B (ovalbumin), member 2 |
| SERPINE2 | serpin peptidase inhibitor, clade E (nexin, plasminogen activator inhibitor type 1), member 2 |
| SERPINF1 | serpin peptidase inhibitor, clade F (alpha-2 antiplasmin, pigment epithelium derived factor), member 1 |
| SERPING1 | serpin peptidase inhibitor, clade G (C1 inhibitor), member 1, (angioedema, hereditary) |
| SERPINI1 | serpin peptidase inhibitor, clade I (neuroserpin), member 1 |
| SET | SET translocation (myeloid leukemia-associated) |
| SEZ6L2 | seizure related 6 homolog (mouse)-like 2 |
| SFRS10 | splicing factor, arginine/serine-rich 10 (transformer 2 homolog, Drosophila) |
| SFRS12 | splicing factor, arginine/serine-rich 12 |
| SGCB | sarcoglycan, beta (43kDa dystrophin-associated glycoprotein) |
| SHC1 | SHC (Src homology 2 domain containing) transforming protein 1 |
| SIRT1 | sirtuin (silent mating type information regulation 2 homolog) 1 (S. cerevisiae) |
| SLC11A1 | solute carrier family 11 (proton-coupled divalent metal ion transporters), member 1 |
| SLC11A2 | solute carrier family 11 (proton-coupled divalent metal ion transporters), member 2 |
| SLC17A7 | solute carrier family 17 (sodium-dependent inorganic phosphate cotransporter), member 7 |
| SLC18A2 | solute carrier family 18 (vesicular monoamine), member 2 |
| SLC18A3 | solute carrier family 18 (vesicular acetylcholine), member 3 |
| SLC1A1 | solute carrier family 1 (neuronal/epithelial high affinity glutamate transporter, system Xag), member 1 |
| SLC1A2 | solute carrier family 1 (glial high affinity glutamate transporter), member 2 |
| SLC1A3 | solute carrier family 1 (glial high affinity glutamate transporter), member 3 |
| SLC2A1 | solute carrier family 2 (facilitated glucose transporter), member 1 |
| SLC2A3 | solute carrier family 2 (facilitated glucose transporter), member 3 |
| SLC30A1 | solute carrier family 30 (zinc transporter), member 1 |
| SLC30A4 | solute carrier family 30 (zinc transporter), member 4 |
| SLC30A6 | solute carrier family 30 (zinc transporter), member 6 |
| SLC5A7 | solute carrier family 5 (choline transporter), member 7 |
| SLC6A1 | solute carrier family 6 (neurotransmitter transporter, GABA), member 1 |
| SLC6A2 | solute carrier family 6 (neurotransmitter transporter, noradrenalin), member 2 |
| SLC6A3 | solute carrier family 6 (neurotransmitter transporter, dopamine), member 3 |
| SLC6A4 | solute carrier family 6 (neurotransmitter transporter, serotonin), member 4 |
| SLC8A1 | solute carrier family 8 (sodium/calcium exchanger), member 1 |
| SMAD3 | SMAD family member 3 |
| SMAD4 | SMAD family member 4 |
| SMPD2 | sphingomyelin phosphodiesterase 2, neutral membrane (neutral sphingomyelinase) |
| SNAP91 | synaptosomal-associated protein, 91kDa homolog (mouse) |
| SNCA | synuclein, alpha (non A4 component of amyloid precursor) |
| SNCB | synuclein, beta |
| SNCG | synuclein, gamma (breast cancer-specific protein 1) |
| SOAT1 | sterol O-acyltransferase (acyl-Coenzyme A: cholesterol acyltransferase) 1 |
| SOAT2 | sterol O-acyltransferase 2 |
| SOD2 | superoxide dismutase 2, mitochondrial |
| SORL1 | sortilin-related receptor, L(DLR class) A repeats-containing |
| SP1 | Sp1 transcription factor |
| SPN | sialophorin (leukosialin, CD43) |
| SPTAN1 | spectrin, alpha, non-erythrocytic 1 (alpha-fodrin) |
| SPTB | spectrin, beta, erythrocytic (includes spherocytosis, clinical type I) |
| SQSTM1 | sequestosome 1 |
| SREBF1 | sterol regulatory element binding transcription factor 1 |
| SRR | serine racemase |
| SST | somatostatin |
| SSTR4 | somatostatin receptor 4 |
| ST3GAL4 | ST3 beta-galactoside alpha-2,3-sialyltransferase 4 |
| ST6GAL1 | ST6 beta-galactosamide alpha-2,6-sialyltranferase 1 |
| STH | saitohin |
| STK16 | serine/threonine kinase 16 |
| STMN2 | stathmin-like 2 |
| STX8 | syntaxin 8 |
| STXBP1 | syntaxin binding protein 1 |
| SV2A | synaptic vesicle glycoprotein 2A |
| SYN1 | synapsin I |
| SYNPO | synaptopodin |
| SYP | synaptophysin |
| SYT1 | synaptotagmin I |
| TAOK1 | TAO kinase 1 |
| TBP | TATA box binding protein |
| TCN1 | transcobalamin I (vitamin B12 binding protein, R binder family) |
| TCN2 | transcobalamin II; macrocytic anemia |
| TCP1 | t-complex 1 |
| TF | transferrin |
| TFAM | transcription factor A, mitochondrial |
| TFCP2 | transcription factor CP2 |
| TFPI | tissue factor pathway inhibitor (lipoprotein-associated coagulation inhibitor) |
| TGFB1 | transforming growth factor, beta 1 (Camurati-Engelmann disease) |
| TGFB2 | transforming growth factor, beta 2 |
| TGM1 | transglutaminase 1 (K polypeptide epidermal type I, protein-glutamine-gamma-glutamyltransferase) |
| TGM2 | transglutaminase 2 (C polypeptide, protein-glutamine-gamma-glutamyltransferase) |
| TGM3 | transglutaminase 3 (E polypeptide, protein-glutamine-gamma-glutamyltransferase) |
| TH | tyrosine hydroxylase |
| THOP1 | thimet oligopeptidase 1 |
| THTPA | thiamine triphosphatase |
| TIAL1 | TIA1 cytotoxic granule-associated RNA binding protein-like 1 |
| TIMP1 | TIMP metallopeptidase inhibitor 1 |
| TKT | transketolase (Wernicke-Korsakoff syndrome) |
| TLR4 | toll-like receptor 4 |
| TMED10 | transmembrane emp24-like trafficking protein 10 (yeast) |
| TMEM147 | transmembrane protein 147 |
| TNF | tumor necrosis factor (TNF superfamily, member 2) |
| TNFAIP1 | tumor necrosis factor, alpha-induced protein 1 (endothelial) |
| TNFRSF1B | tumor necrosis factor receptor superfamily, member 1B |
| TNFSF10 | tumor necrosis factor (ligand) superfamily, member 10 |
| TNNC1 | troponin C type 1 (slow) |
| TNXA | tenascin XA pseudogene |
| TP53 | tumor protein p53 (Li-Fraumeni syndrome) |
| TP73 | tumor protein p73 |
| TPH1 | tryptophan hydroxylase 1 (tryptophan 5-monooxygenase) |
| TPK1 | thiamin pyrophosphokinase 1 |
| TPT1 | tumor protein, translationally-controlled 1 |
| TRADD | TNFRSF1A-associated via death domain |
| TREM2 | triggering receptor expressed on myeloid cells 2 |
| TRIM11 | tripartite motif-containing 11 |
| TRPC6 | transient receptor potential cation channel, subfamily C, member 6 |
| TSC2 | tuberous sclerosis 2 |
| TSC22D3 | TSC22 domain family, member 3 |
| TTR | transthyretin (prealbumin, amyloidosis type I) |
| TUBB3 | tubulin, beta 3 |
| TXN | thioredoxin |
| TXNRD1 | thioredoxin reductase 1 |
| TYRP1 | tyrosinase-related protein 1 |
| UBB | ubiquitin B |
| UBC | ubiquitin C |
| UBE2L1 | ubiquitin-conjugating enzyme E2L 1 |
| UBE2L3 | ubiquitin-conjugating enzyme E2L 3 |
| UBQLN1 | ubiquilin 1 |
| UCHL1 | ubiquitin carboxyl-terminal esterase L1 (ubiquitin thiolesterase) |
| UCHL3 | ubiquitin carboxyl-terminal esterase L3 (ubiquitin thiolesterase) |
| UQCRC1 | ubiquinol-cytochrome c reductase core protein I |
| USP1 | ubiquitin specific peptidase 1 |
| UTP11L | UTP11-like, U3 small nucleolar ribonucleoprotein, (yeast) |
| VDAC2 | voltage-dependent anion channel 2 |
| VDR | vitamin D (1,25- dihydroxyvitamin D3) receptor |
| VEGFA | vascular endothelial growth factor A |
| VGF | VGF nerve growth factor inducible |
| VLDLR | very low density lipoprotein receptor |
| VPS26A | vacuolar protein sorting 26 homolog A (S. pombe) |
| VPS35 | vacuolar protein sorting 35 homolog (S. cerevisiae) |
| VPS41 | vacuolar protein sorting 41 homolog (S. cerevisiae) |
| VSNL1 | visinin-like 1 |
| VTNR | vitronectin receptor |
| WARS | tryptophanyl-tRNA synthetase |
| WASF1 | WAS protein family, member 1 |
| WASL | Wiskott-Aldrich syndrome-like |
| WEE1 | WEE1 homolog (S. pombe) |
| WNK1 | WNK lysine deficient protein kinase 1 |
| WT1 | Wilms tumor 1 |
| WWOX | WW domain containing oxidoreductase |
| XYLT2 | xylosyltransferase II |
| YLPM1 | YLP motif containing 1 |
| YY1 | YY1 transcription factor |
| ZMPSTE24 | zinc metallopeptidase (STE24 homolog, S. cerevisiae) |
